# PARP inhibitor era in ovarian cancer treatment: a systematic review and meta-analysis of randomized controlled trials

**DOI:** 10.1186/s13048-024-01362-y

**Published:** 2024-02-26

**Authors:** István Baradács, Brigitta Teutsch, Alex Váradi, Alexandra Bilá, Ádám Vincze, Péter Hegyi, Tamás Fazekas, Balázs Komoróczy, Péter Nyirády, Nándor Ács, Ferenc Bánhidy, Balázs Lintner

**Affiliations:** 1 Department of Obstetrics and Gynecology, Semmelweis University, Üllői út 78/A, Budapest, H-1082 Hungary; 2 Centre for Translational Medicine, Semmelweis University, Budapest, Hungary; 3 Institute for Translational Medicine, Szentágothai Research Centre, Medical School, University of Pécs, Pécs, Hungary; 4 Division of Pancreatic Diseases, Heart and Vascular Center, Semmelweis University, Budapest, Hungary; 5 Department of Urology, Semmelweis University, Budapest, Hungary; 6 School of Medicine, Semmelweis University, Budapest, Hungary

**Keywords:** Olaparib, Niraparib, Rucaparib, HRD, Homologous recombination repair

## Abstract

**Background:**

Ovarian cancer is the eighth leading cause of cancer-related death among women, characterized by late diagnosis and a high relapse rate. In randomized controlled trials, we aimed to evaluate the efficacy and safety of PARP inhibitors (PARPi) in treating advanced ovarian cancer.

**Methods:**

This review was registered on PROSPERO (CRD42021283150), included all phase II and phase III randomized controlled trials (RCTs) assessing the effect of PARPi on ovarian cancer until the 13th of April, 2022. The main outcomes were progression- free survival (PFS), overall survival (OS), and adverse events (AEs). Pooled hazard ratios (HRs), and risk ratios (RRs) were calculated with 95% confidence intervals (95% CI). The random-effects model was applied in all analyses.

**Results:**

In the meta-analysis, 16 eligible RCTs were included, with a total of 5,815 patients. In recurrent ovarian cancer, PARPi maintenance therapy showed a significant PFS benefit over placebo in the total population (HR 0.34, CI 0.29–0.40), BRCA mutant (HR 0.24, CI 0.18–0.31), germline BRCA mutant (HR 0.23, CI 0.18–0.30), and BRCA wild-type cases (HR 0.50, CI 0.39–0.65). PARPi monotherapy also improved PFS (HR 0.62, CI 0.51–0.76) compared with chemotherapy in BRCAm patients with recurrent ovarian cancer. The use of PARPi maintenance therapy resulted in an improvement in PFS over placebo in newly-diagnosed cancers in the overall population (HR 0.46, CI 0.30–0.71) and the BRCAm population (HR 0.36, CI 0.29–0.44). Although the risk of severe AEs was increased by PARPi therapy compared to placebo in most settings investigated, these side effects were controllable with dose modification, and treatment discontinuation was required in the minority of cases.

**Conclusions:**

PARPis are an effective therapeutic option for newly-diagnosed and recurrent ovarian cancer. Despite a minor increase in the frequency of serious adverse effects, they are generally well tolerated.

**Supplementary Information:**

The online version contains supplementary material available at 10.1186/s13048-024-01362-y.

## Introduction

Ovarian cancer (OC) is the third most frequent gynecological malignancy and the eighth leading cause of cancer-related death among women. Globally, the number of new cases was 313,959, and 207,252 died from the disease in 2020 [1]. Epithelial ovarian cancers account for 90% of ovarian cancer cases, and approximately 75% of them are high-grade serous carcinoma (HGSC) [2]. The cellular site of origin of HGSC is now thought to be the fallopian tube fimbriae, and direct access to the peritoneum leads to rapid spread [3, 4]. Due to its asymptomatic nature and ineffective screening, the diagnostic challenge could lead to advanced cancer stage (stage III or IV) detection in nearly 75% of cases [5]. 

The initial treatment for advanced ovarian cancer involves cytoreductive surgery and systematic chemotherapy. Despite the high responsiveness, more than 70% of patients with advanced OC will experience recurrence and develop chemo-resistance over time [6, 7]. Cancer biology research highlighted the role of *BRCA1/2* mutations and homologous recombination deficiency (HRD), which opened the field for targeted therapies such as poly (ADP-ribose) polymerase (PARP) inhibitors (PARPi). The Cancer Genome Atlas shows that approximately 50% of HGSC patients have HRD, including germline and somatic *BRCA1/2* mutation in 11–15% and 7% of women, respectively [8]. The inhibition of PARP (a family of enzymes primarily involved in DNA repair) propagates single-strand DNA breaks and the accumulation of double-strand breaks, requiring repair by homologous recombination repair (HRR). However, the pathogenic variants of *BRCA1/2* or other forms of HRD make cancer cells particularly sensitive to PARPi, the mechanism is called synthetic lethality, and the concurrent loss of both repair pathways leads to cell death. Three PARPis are approved globally for the treatment of ovarian, fallopian tube, or primary peritoneal cancer (referred together as ovarian cancer): olaparib, rucaparib and niraparib [9, 10]. 

The study investigated PARPis in the most homogeneous patient groups possible. Compared to previous meta-analyses in which the different therapeutic settings, cancer types, or *BRCAm*, *BRCAw*, and HRD patients were pooled, our results provide a more transparent picture of the therapy characteristics [11–13]. 

This systematic review and meta-analysis was conducted to analyze the data of randomized controlled trials (RCTs) on the beneficial and harmful effects of PARPis in terms of survival and adverse events in different subgroups of patients.

## Methods

The study was reported according to the Preferred Reporting Items for Systematic Reviews and Meta-Analysis (PRISMA) 2020 Statement (see Table S1) [14]. The research was conducted according to the recommendations for Systematic Reviews of Intervention in the Cochrane Handbook [15]. The review protocol was registered on PROSPERO under the registration number of CRD42021283150.

### Search strategy

We performed a systematic literature search of electronic databases, including MEDLINE (via PubMed), EMBASE, and Cochrane Library, from inception to the 13th of April, 2022. Search terms included all types of PARPis and ovarian cancer-related terms (see Appendix S1). Reference lists of the eligible articles were also manually screened to capture all potentially relevant trials. We did not use language or other restrictions.

### Eligibility criteria, selection, and data collection

All RCTs were found eligible which: (1) investigated patients diagnosed with advanced cancer of the ovary, peritoneum, or fallopian tube; (2) provided data on newly-diagnosed or recurrent cases in terms of overall survival (OS), progression-free survival (PFS), or adverse events (AEs) (anaemia, thrombocytopenia, neutropenia, leukopenia, vomiting, fatigue, and nausea); (3) had used PARPi as an intervention in monotherapy, maintenance therapy, or as an addition to standard-of-care therapies. We excluded studies that were: (1) phase I RCTs; (2) conference abstracts without reliable data on study design; and (3) tested PARPis in combination with other targeted therapeutic drugs.

After removing duplicates, two authors (IB, and ÁV) made the selection by titles and abstracts, and full texts independently of each other while adhering to the eligibility criteria. Cohen’s kappa coefficient was calculated at each selection phase [16]. A third reviewer (BL) resolved the differences.

Study and outcome data were extracted into a pre-defined data collection form by the two authors (IB, and ÁV). A third researcher (BL) resolved disagreements. The following data were collected from each article: first author’s name, publication year, study design, trial name, number of patients, age, outcomes (PFS, OS, and AE) and the related raw data, risk ratios (RRs) or hazard ratios (HRs) with 95% confidence intervals (CI), therapy settings, number of different mutations, and the number of every recorded AEs and grade on a 5-level scale according to National Cancer Institute Common Terminology Criteria for Adverse Events (NCI CTCAE). For a given outcome, we only used data from a single article, the one that met our criteria and had the longest follow-up time. The statistical analysis was based only on published data. No data were requested from the authors directly.

### Risk of bias and study quality assessment

Two authors (IB, and ÁV) independently assessed the quality of all included studies using the Revised Cochrane risk-of-bias tool for RCTs (RoB 2) [17]. Bias was evaluated in five primary domains: randomization process, deviations from intended interventions, missing output data, outcome measurement, and selection of reported results. Disagreements between the evaluators were resolved through dialogue and, if necessary, the participation of a third reviewer (AB).

We used the Grading of Recommendations Assessment, Development, and Evaluation (GRADE) method to evaluate the reliability of the evidence [18]. Two independent review writers (IB, and ÁV) examined each assessment criterion for each outcome and comparison. A neutral arbitrator solved any controversy (BL).

### Statistical analysis

In the case of PFS and OS, we pooled hazard ratios (HRs) with 95% confidence intervals (CI) using random effects models with the inverse variance method based on estimates of log HRs and their standard errors. To estimate the between-study variance tau^2^, we applied restricted maximum-likelihood estimator [19]. For binary outcome data (adverse events), random effects estimates of risk ratios (RRs) with 95% CI were calculated using the exact Mantel-Haenszel method [20–22]; hence, we did not apply continuity correction to handle zero cell counts [23, 24]. By the recommendation of Veroniki et al. 2016 [25], the Paule-Mandel method [26] was used to estimate heterogeneity variance measure tau^2^. For the outcomes where the study number was over 5, a Hartung-Knapp adjustment [27, 28] was used. We did not apply any adjustment below five studies. Where applicable, we reported the prediction intervals (i.e., the expected range of effects of future studies) of results following the recommendations of IntHout et al. 2016 [29]. 

The statistical analysis of the data was conducted using the R programming language (R Core Team, 2021, Vienna, Austria, R version 4.1) using the meta [30] and dmetar [31] packages. The results of the meta-analysis were illustrated using forest plots, and aggregated-forest plots. The results were deemed statistically significant if the *p*-value was less than 0.05. Heterogeneity was evaluated using I² statistics and χ² tests, where a *p*-value less than 0.1 indicated significant heterogeneity [32]. 

For every outcome, a minimum of three studies were required to perform a meta-analysis.

To create study groups that were as homogeneous as possible, the two large groups of recurrent and newly-diagnosed tumors were further subdivided by therapy settings and BRCA mutation status.

## Results

### Search and selection

**Fig. 1:**
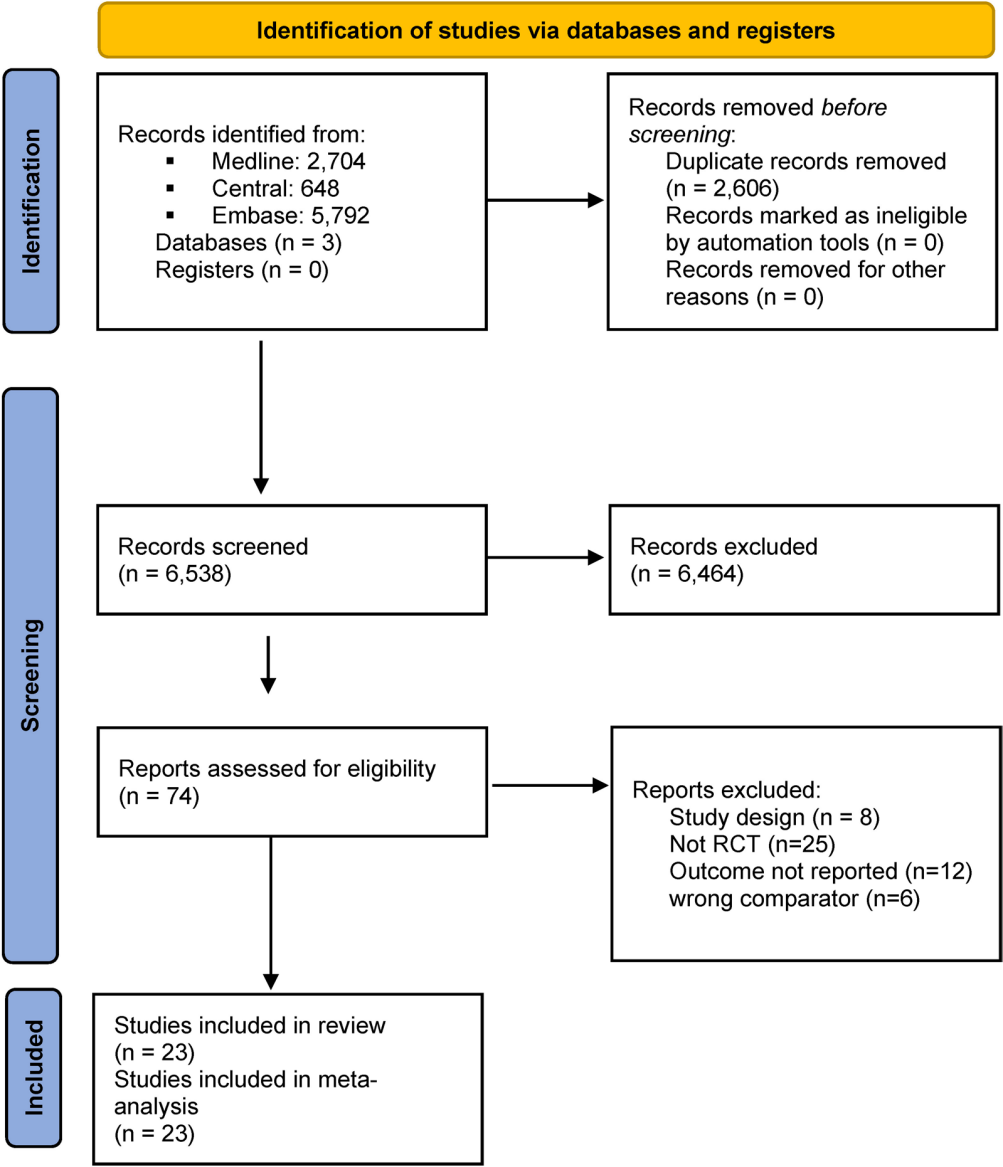
PRISMA 2020 flowchart representing the study selection process

Based on our search strategy, we identified 9,144 records. After rigorous selection, 23 articles reporting about 16 trials were found eligible for systematic review and meta-analysis. The PRISMA flowchart of the selection process is summarized in Fig. 1.

### Basic characteristics of included studies

In total, 5,815 patients were evaluated across all trials. The baseline characteristics of the selected studies are detailed in Table 1.

**Table 1:** Basic characteristics of included studies

No	Trial	Year	Article(s)	Phase	Study design	Population (Intervention/Control)	Disease state (newly-diagnosed/recurrent)	Intervention arm	Control arm
1.	VELIA	2019	Coleman et al. (2019) [33]	III	randomized, double-blind, placebo- controlled	1,140 (382/383/375)	Newly-diagnosed	PBC + Veliparib 150 orally twice a day + Veliparib 300–400 mg orally twice day as maintenance (382) or PBC + Veliparib 150 mg orally twice a day + placebo as maintenance	PBC + placebo daily + placebo as maintenance
2.	ARIEL 3	2017	Coleman et al. (2017) [34]; Ledermann et al. (2020) [35]	III	randomized, double-blind, placebo- controlled	564 (375/189)	Recurrent	Rucaparib 600 mg orally twice a day	Placebo
3.	Study 19	2012	Ledermann et al. (2012, 2014, 2016) [36–38]; Friedlander et al. (2018) [39]	II	randomized, double-blind, placebo- controlled	265 (136/129)	Recurrent	Olaparib 400 mg orally twice a day	Placebo
4.	PRIMA	2019	González et al. (2019) [40]	III	randomized, double-blind	733 (487/246)	Newly-diagnosed	Niraparib 300/200 mg orally twice a day	Placebo
5.	ICEBERG 3	2012	Kaye et al. (2012) [41]	II	randomized, open-label	65 (32/33)	Recurrent	Niraparib 400 mg orally twice a day	PLD
6.	NOVA	2016	Mirza et al. (2016, 2020) [10, 42, 43]	III	randomized, double-blind, placebo- controlled	553 (372/181)	Recurrent	Niraparib 300 mg orally daily	Placebo
7.	SOLO 1	2018	Moore et al. (2018) [44]; Banerjee et al. (2021) [45]	III	randomized, double-blind, placebo- controlled	391 (260/131)	Newly-diagnosed	Olaparib 300 mg orally twice a day	Placebo
8.	Oza 2015	2015	Oza et al. (2015) [46]	II	randomized, open-label	162 (81/81)	Recurrent	PBC + Olaparib 200 mg orally twice a day + Olaparib 400 mg orally twice a day as maintenance	PBC
9.	SOLO 3	2020	Penson et al. (2020) [47]	III	randomized, open-label	266 (178/88)	Recurrent	Olaparib 300 mg orally twice a day	Single agent chemotherapy
10.	SOLO 2	2020	Pujade-Lauraine et al. (2017) [48]; Poveda et al. (2021) [49]	III	randomized, double-blind	295 (196/99)	Recurrent	Olaparib 300 mg orally twice a day	Placebo
11.	SOLO 1 China	2021	Wu L, et al. (2021) [50]	III	randomized, double-blind, placebo- controlled	64 (44/20)	Newly-diagnosed	Olaparib 300 mg orally twice a day	Placebo
12.	NORA	2021	Wu X, et al. (2021) [51]	III	randomized, double-blind, placebo- controlled	265 (177/88)	Recurrent	Niraparib 100 mg three times a day	Placebo
13.	FZOCUS-2	2022	Ning Li et al. (2022) [52]	III	randomized, double-blind, placebo- controlled	252 (167/85)	Recurrent	Fuzuloparib 150 mg orally twice a day	Placebo
14.	ARIEL 4	2022	Kristeleit et al. (2022) [53]	III	randomized, open-label	349 (233/116)	Recurrent	Rucaparib 600 mg orally twice a day	Paclitaxel or PBC
15.	NRG-GY004	2022	Liu et al. (2022) [54]	III	randomized, open-label	376 (189/187)	Recurrent	Olaparib 300 mg orally twice a day	PBC
16.	Kummar 2015	2015	Kummar et al. (2015) [55]	II	randomized, open-label	75 (37/38)	Recurrent	Cyclophosphamide + Veliparib 60 mg orally tablets daily	Cyclophosphamide daily

*PBC* Platinum-based chemotherapy, *PLD* Pegylated liposomal doxorubicin

### Survival analysis

#### Recurrent ovarian cancer

Twelve studies assessed the effect of PARPi on recurrent ovarian cancer.

##### PARPi as maintenance vs. placebo (after chemotherapy)

**Fig. 2:**
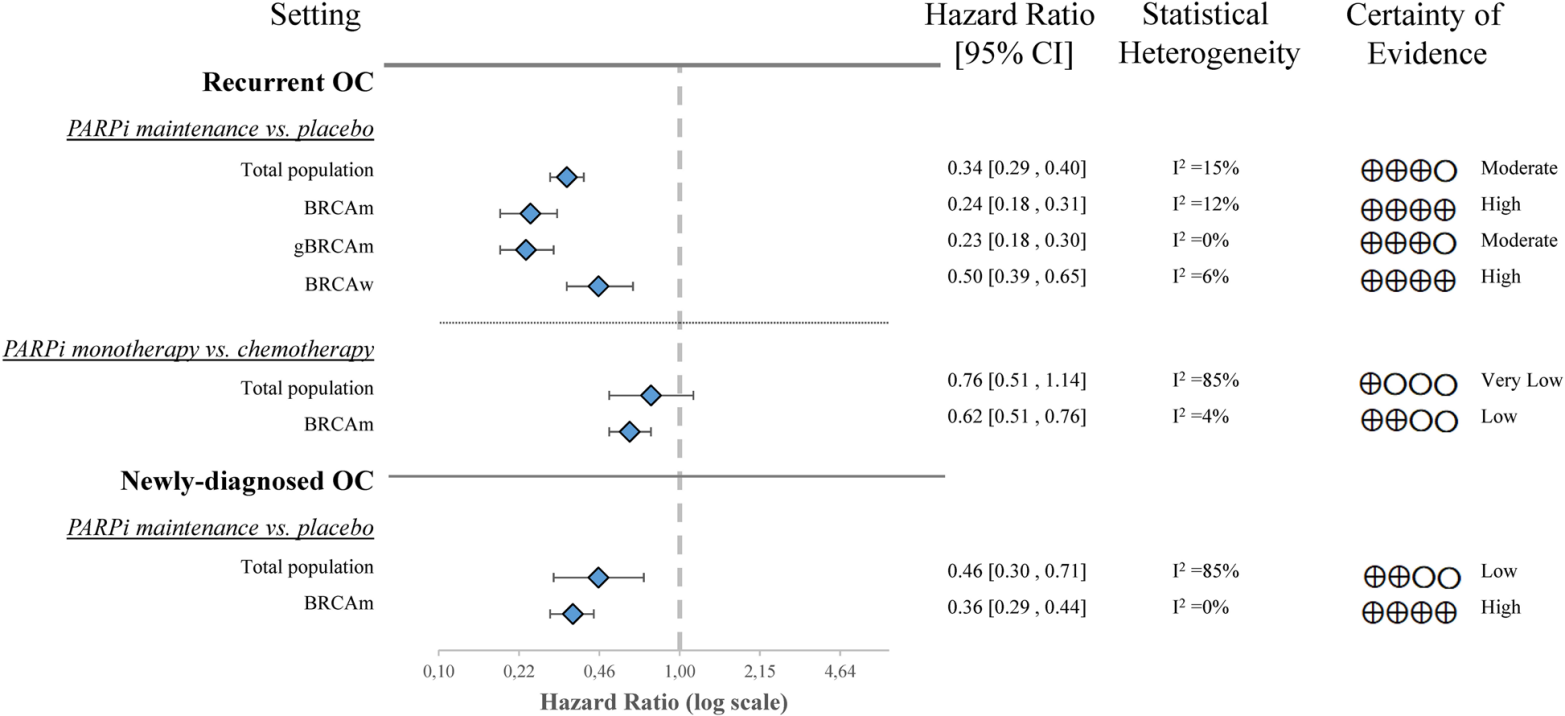
Forest plot of aggregated data representing the hazard ratios of disease progression in recurrent OC and newly-diagnosed OC. OC-ovarian cancer, PARPi-poly ADP ribose polymerase inhibitor, *BRCAm*-breast cancer gene mutation, *gBRCAm*-germline breast cancer gene mutation, *sBRCAm*-somatic breast cancer gene mutation, *BRCAw*- breast cancer gene wild-type, CI-confidential interval

 According to the results of seven clinical trials [18–29], PARPi significantly improved PFS compared to placebo in the total population (HR 0.34, CI 0.29–0.40). We observed statistically significant benefits of PARPi impact among the *BRCAm* (HR 0.24, CI 0.18–0.31), *gBRCAm* (HR 0.23, CI 0.18–0.30), and *BRCA* wild-type (HR 0.50, CI 0.39–0.65) subgroups [34–39, 42, 43, 48, 49, 51, 52] (Fig. 2; Figures S1-S4).

Only two articles reported information about the OS, finding better but not statistically significant results with PARPi: Study 19 (HR 0.73 CI 0.55–0.95) SOLO 2 (HR 0.74 CI 0.54-1.0) [38, 49]. 

##### PARPi monotherapy vs. chemotherapy

This setting comprised 1,056 patients in four separate RCTs. PARPi did not significantly increase the PFS in the total population (HR 0.76, CI 0.51–1.14). However, the impact of PARPi on the PFS of *BRCA*-mutated patients was more substantial (HR 0.6, CI 0.51–0.76) [41, 47, 53, 54] (Fig. 2; Figures S5-S6).

On the other hand, PARPi monotherapy, compared with chemotherapy, did not result in increased OS in ICEBERG 3 (HR 1.01, CI 0.44–2.27) and SOLO 3 (HR 1.07, CI 0.65–1.76) trials [41, 47]. 

##### PARPi with chemotherapy vs. chemotherapy alone

The addition of PARPi to chemotherapy versus chemotherapy alone was examined in one study, where the PFS was similar in the two groups (HR 1.02, CI 0.69–1.50) [55]. 

##### PARPi with chemotherapy plus PARPi maintenance vs. chemotherapy alone

In one study utilizing this therapeutic setting, PARPi treatment combined with chemotherapy and continued as maintenance treatment significantly increased PFS compared to chemotherapy (HR 0.51, CI 0.34–0.77). Among *BRCAm* patients, the benefit from the intervention was even more substantial for PFS (HR 0.21, CI 0.08–0.55) [46]. 

As for OS, neither the total population (HR 1.17, CI 0.79–1.73) nor *BRCAm* patients (HR 1.28, CI 0.39–4.18) showed a significant difference in favor of the intervention [46]. 

#### Newly-diagnosed ovarian cancer

Four trials assessed the efficacy of PARPi in newly diagnosed, advanced OC [40, 44, 45, 50]. 

##### PARPi as maintenance vs. placebo (after chemotherapy)

Front line maintenance setting of PARPi showed a significant PFS benefit in three RCTs over placebo in newly-diagnosed patients following chemotherapy (HR 0.46, CI 0.30–0.71). The benefit was even more pronounced in patients with *BRCAm* (HR 0.36, CI 0.29–0.44). The PRIMA trial examined 150 patients with *BRCA* wild-type cases and observed a significant PFS advantage in the intervention group (HR 0.50, CI 0.31–0.83) [40, 44, 45, 50] (Fig. 2; Figures S7-S8).

OS data were reported in two studies. Neither PRIMA (HR 0.7, CI 0.44–1.11) nor SOLO 1 (HR 0.95, CI 0.6–1.53) reported a significant OS benefit [40]. 

##### PARPi in combination with chemotherapy plus placebo maintenance vs. chemotherapy with placebo plus placebo maintenance

In the VELIA trial, patients were randomly assigned to three groups; the second and third groups compared PARPi plus chemotherapy combination to chemotherapy. In the intention-to-treat (ITT) (HR 1.07, CI 0.90–1.29), *BRCAm* (HR 1.22, CI 0.82–1.80), and *BRCAw* (HR 1.04, CI 0.84–1.29) populations, there were no differences in PFS [33]. 

##### PARPi in combination with chemotherapy plus PARPi maintenance vs. chemotherapy with placebo plus placebo maintenance

In the first and third arms of the VELIA trial, the combination of PARPi and chemotherapy was enhanced with PARPi maintenance therapy, resulting in a statistically significant PFS advantage in this group of patients, including ITT (HR 0.68, CI 0.56–0.83), *BRCAm* (HR 0.44, CI 0.28–0.68), and *BRCAw* (HR 0.80, CI 0.64–1.00) individuals [33]. 

### Severe adverse events

In compiling the safety part of our meta-analysis, we examined the total number of adverse events, the four most common hematologic, the three most common toxicity, and those leading to dose modification, dose interruption, treatment discontinuation, and aggregate incidence of myelodysplastic syndrome and acute myeloid leukemia (MDS/AML). Severe (grade 3 or 4) adverse events are detailed below. The total numbers of these toxicities, regardless of the grade are shown in Figures S9-S62 of the supplement.The risk of these adverse events was evaluated in three therapeutic settings of PARPi.

#### Grade 3 and 4 AEs: recurrent ovarian cancer

##### PARPi as maintenance vs. placebo (after chemotherapy)

**Fig. 3:**
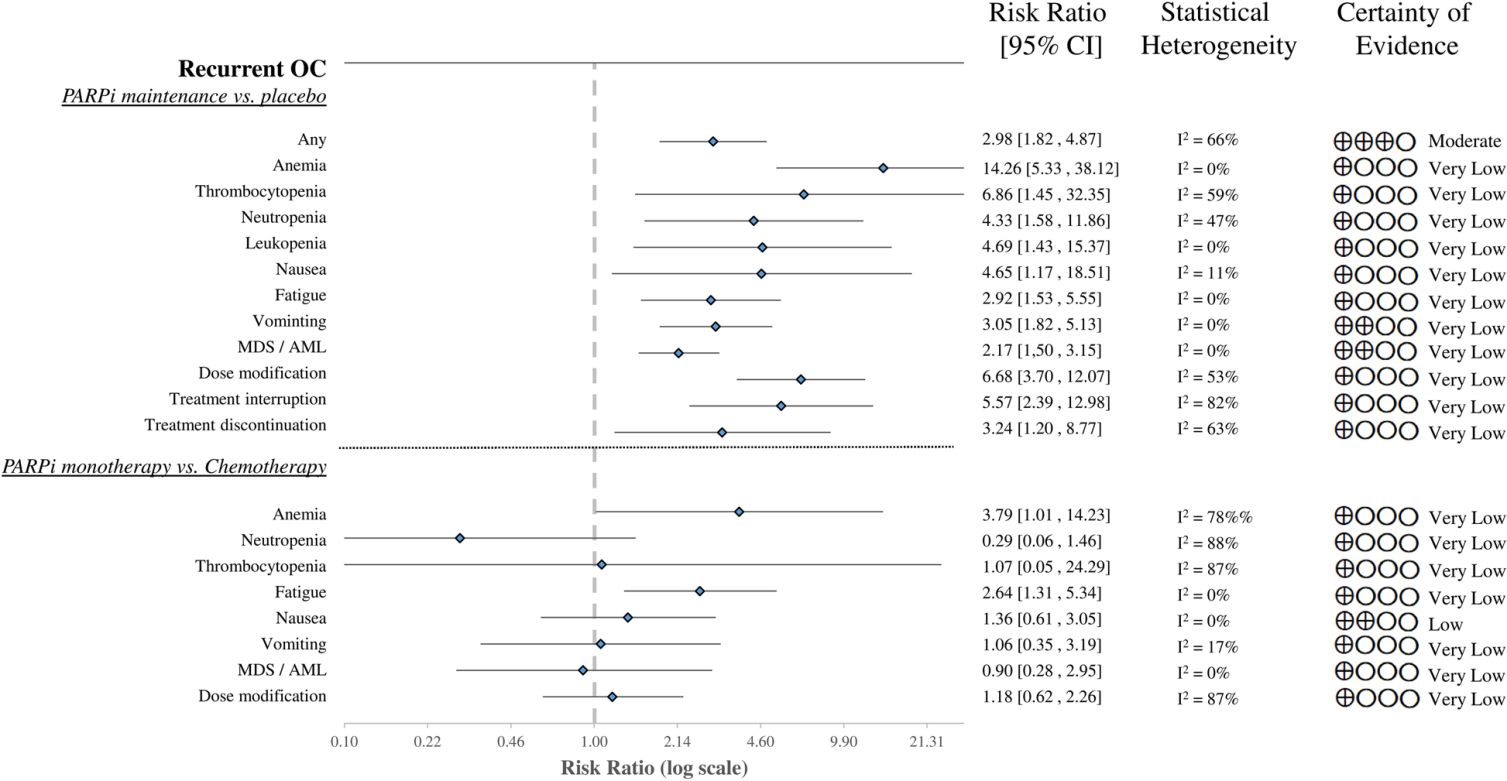
Forest plot of aggregated data representing the risk ratios of Grade 3 ≤ adverse events in recurrent OC. OC-ovarian cancer, PARPi-poly ADP ribose polymerase inhibitor, MDS / AML- myelodysplastic syndrome or acute myeloid leukemia, CI-confidential interval

 Maintenance treatment of PARPi following chemotherapy for recurrent ovarian carcinoma significantly increased the risk of severe adverse events compared to placebo (RR 2.98, CI 1.82–4.87). Each of the four examined hematological toxicities, anemia (RR 14.26, CI 5.33–38.12), thrombocytopenia (RR 6.86, CI 1.45–32.35), neutropenia (RR 4.33, CI 1.58–11.86), and leukopenia (RR 4.69; C: 1.43–15.37), developed with a higher risk on the intervention arm. The risks for the three most frequent adverse events, nausea (RR 4.65, CI 1.17–18.51), fatigue (RR 2.92, CI 1.53–5.55), vomiting (RR 3.05, CI 1.82–5.13), and the MDS/AML (RR 2.17, CI 1.50–3.15) were significantly elevated in the PARPi group. Furthermore, the risk of AEs leading to dose modification (RR 6.68, CI 3.70–12.07), treatment interruption (RR 5.57, CI 2.39–12.98), and treatment discontinuation (RR 3.24, CI 1.20–8.77) was also increased after the intervention [35, 39, 42, 49, 51, 52] (Fig. 3; Figures S9-S29).

##### PARPi Monotherapy vs. chemotherapy

In patients treated with PARPi monotherapy compared to chemotherapy, the risk of severe anemia (RR 3.79, CI 1.01–14.23) was considerably higher among those with hematological toxicities, whereas there was no significant difference in neutropenia (RR 0.29, CI 0.06–1.46) and thrombocytopenia (RR 1.07, CI 0.05–24.29). For the three common AEs, the risk of fatigue (RR 2.64, CI 1.31–5.34) increased significantly, but no significant difference was detected for nausea (RR 1.36, CI 0.61–3.05) and vomiting (RR 1.06, CI 0.35–3.19;). The intervention resulted in no difference in dose modification (RR 1.18, CI 0.62–2.26) compared to chemotherapy. The risk of developing MDS and AML was also similar in both groups (RR 0.90, CI 0.28–2.95) [41, 47, 53, 54] (Fig. 3; Figures S30-S43).

#### Grade 3 and 4 AEs: newly-diagnosed ovarian cancer

##### PARPi as maintenance vs. placebo (after chemotherapy)

**Fig. 4:**
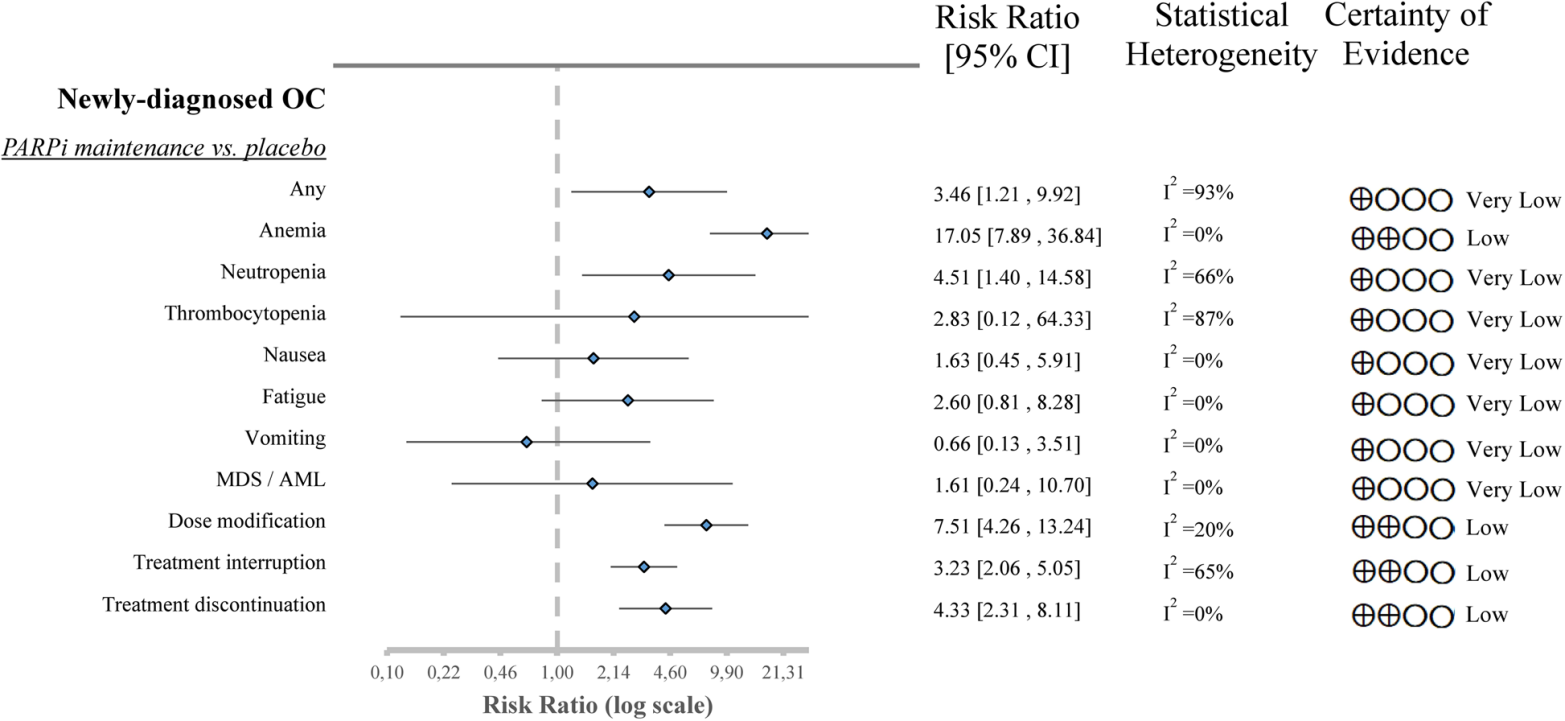
Forest plot of aggregated data representing the risk ratios of grade 3 ≤ adverse events in newly-diagnosed OC. OC-ovarian cancer, PARPi-poly ADP ribose polymerase inhibitor, MDS / AML- myelodysplastic syndrome or acute myeloid leukemia, CI-confidential interval

 First-line maintenance PARPi therapy increased the pooled risk ratio of grade 3 and grade 4 AEs (RR 3.46, CI 1.21–9.92) compared to placebo. In hematological toxicities, the risk of developing anemia (RR 17.05, CI 7.89–36.84) and neutropenia (RR 4.51, CI 1.40–14.58) was increased, although the risk of thrombocytopenia (RR 2.83, CI 0.12–64.33) did not significantly differ between the two groups. In this intervention group, PARPi did not significantly elevate nausea (RR 1.63, CI 0.45–5.91), fatigue (RR 2.60, CI 0.81–8.28), and vomiting (RR 0.66, CI 0.13–3.51). However, with PARPi, dose modification (RR 7.51, CI 4.26–13.24), treatment interruption (RR 3.23, CI 2.06–5.05), and treatment discontinuation (RR 4.33, CI 2.31–8.11) due to toxicities were substantially more likely to occur. MDS and AML (RR 1.61, CI 0.24–10.70) were relatively rare, and there was no difference between PARPi and placebo [40, 44, 45, 50] (Fig. 4; Figures S44-S62).

### Risk of bias assessment

The findings of the evaluation of the risk of bias are reported in Figure S63. The overall risk of bias was low, but we have to mention that there were six open-label trials.

### Heterogeneity and quality of evidence

The findings of heterogeneity are displayed in the figures corresponding to the evaluated outcomes (Figs. 2, 3 and 4; Figures S1-S62). We describe the quality of evidence in detail in Supplementary Tables S2-S7, and summarize the results for each outcome in the certainty of evidence column in Figs. 2, 3 and 4.

## Discussion

Our meta-analysis has demonstrated that PARPi therapy can provide significant PFS benefits over placebo in patients with recurrent and newly-diagnosed advanced OC when used as maintenance therapy. These advantages were no longer evident in the therapeutic settings when PARPi was compared to chemotherapy, although the improvement in PFS with maintenance therapy was accompanied by an increase in grade 3 and grade 4 adverse events. While PARPi did not appear to have a significantly worse toxicity profile than chemotherapy, the advantages for OS were no longer evident with PARPi in this therapeutic setting. Most AEs could be managed by dose modification, after which therapy could be continued, resulting in treatment discontinuation occurring only in a significantly smaller proportion of cases.

In patients receiving PARPi maintenance treatment for newly-diagnosed and recurrent malignancies, regardless of the presence or absence of *BRCA* mutations, disease progression occurs significantly later than in the placebo group. The most apparent evidence is in patients with germline *BRCA* mutations; nevertheless, we also observed a significant improvement in PFS in *BRCA* wild-type cases. However, these results may be inconclusive because a substantial proportion of the individuals evaluated could have a form of HRD, known to be susceptible to PARPi. We could not conduct a meta-analysis for the HRD subgroup due to the lack of at least three studies in any of the treatment settings. Only four trials provide data for the HRD group, corresponding to three fundamentally distinct therapy scenarios [33, 34, 40, 42]. Furthermore, there are significant differences in the composition of the HRD groups, in the NOVA [42] trial the *sBRCAm* plus those with non-*BRCA*-related HRD were considered the HRD population, in the ARIEL 3 [34] *BRCAm* plus *BRCAw* with high percentage of loss of heterozygosity (a type of mutation that causes the deletion of one copy of a DNA region) carcinomas. The ARIEL 3 study assessed the efficacy of rucaparib in patients with recurrent OC and HRD, and reported a median PFS of 13.6 months compared to 5.4 months in the placebo group [34]. In the PRIMA [40] the definition of HRD included the presence of a BRCA deleterious mutation, and a score of at least 42 on the my-Choice test, this specific cut-off value does not necessarily mean that anyone who falls outside of it will be homologous recombination proficient (HRP) and will not respond well to PARPi treatment. This assertion is supported by the fact that niraparib improved PFS (HR 0.68, CI 0.49–0.94) compared to placebo in HRP group of this research. The median PFS values for HRD group were 21.9 months and 10.4 months, respectively for the niraparib and placebo arm [40]. Examining this group may become a high priority in the future. Notably, we will need more evidence if future decisions on using PARPi treatment are made solely based on homologous recombination status.

Moreover, it would be crucial to have accurate, high-quality data for the OS as well. The primary outcome of the studies is PFS. However, it is uncertain whether the use of these medications genuinely provides more prolonged survival in different patient groups or can only delay the time of disease progression [56]. Due to short follow-up, data on actual survival time are immature. In this regard, the SOLO 1 [45] study is outstanding, with 48% of patients in the olaparib arm and 21% of patients in the placebo arm having no disease progression at the 5-year follow-up, and the median PFS is 56 months vs. 13.8 months. Testing for PFS is quicker and cheaper, and a smaller population is sufficient to achieve significant test results. We may not even get to know the OS values, at least in most cases, this certainly did not happen until the approval of the drugs.

The FDA- and EMA-approved PARPis in clinical use are olaparib, niraparib, and rucaparib. In recurrent ovarian cancer, the three PARPi are used as maintenance therapy in completely or partially platinum sensitive (objective response to prior platinum-based therapy for more than 6 months). The use of olaparib is not dependent on a biomarker test, as the results of Study 19 [36] demonstrated a substantial improvement in PFS (HR 0.35, CI 0.25–0.49) compared to placebo in the total study population (not just BRCAm). Niraparib could be utilized in gBRCAm cases based on the PFS improvement (HR 0.26, CI 0.17–0.41) published in the NOVA [42] trial. Rucaparib is an approved treatment in sBRCAm or gBRCAm based on the efficacy (HR 0.23, CI 0.16–0.34) seen in the ARIEL 3 [34] cohort of patients with the mutations.

For newly diagnosed ovarian cancer, olaparib, olaparib plus bevacizumab and niraparib are accepted as first-line maintenance therapy if patients have a partial or complete response to platinum-based chemotherapy. Olaparib could be used in the presence of sBRCAm or gBRCAm due to the PFS improvement (HR 0.30, CI 0.23–0.41) reported in SOLO 1 [44]. The combination of olaparib plus bevacizumab is used in HRD cases, with reference to the results of the PAOLA-1 [57] study where combination therapy significantly reduced the hazard of disease progression or death in HRD/BRCAm (HR 0.33, CI 0.25–0.45) and HRD/BRCAw (HR 0.43, CI 0.28–0.66) cases compared to placebo plus bevacizumab. Niraparib use as first-line maintenance therapy is based on platinum sensitivity, with no biomarker testing required, due to the PFS improvement demonstrated by the PRIMA [40] study in the overall population (HR 0.62, CI 0.50–0.76) and HRD population (HR 0.43, CI 0.31–0.59).

In the safety profile analysis, the most frequent AEs identified were anemia, neutropenia, fatigue, vomiting, and nausea, and most of the reviewed research concurred. In most cases, the severity of these toxicities ranged from mild to moderate. In general, our study found that PARPi therapy was well tolerated. High-grade AEs, regardless of the type of event, were also increased by the intervention compared to placebo in newly-diagnosed and recurrent cancers. However, little to no difference was identified between PARPi and chemotherapy for recurrent disease. With PARPi treatment, an elevated risk of hematological toxicities is known to exist [58]. The observed clinical picture is also supported by laboratory models illustrating that the PARP2 enzyme plays an essential role in hematopoiesis [59]. There is currently no specific method for predicting patients at high risk of developing toxicities. It is recommended that the blood counts of treatment participants be continuously monitored to avoid discontinuation of treatment. The risk of MDS and AML increased only in recurrent tumors compared to placebo. These events could occur in small numbers partly due to short follow-up periods. In the future, myelosuppression, along with secondary malignancies, needs to be further explored to understand better and prevent the development of these fatal conditions [60]. 

Our meta-analysis is outstanding in that it investigates the effect of PARPi in all potential clinical settings for ovarian cancer. In order to ensure that the results accurately reflect reality, the subgroups were intended to produce the most homogeneous populations possible. In the majority of published meta-analyses, the subgroups are combined, diminishing the evidence for the results.

As for the limitation of our study, clinical heterogeneity was present in the selected RCTs (different PARPi, previous treatments, and outcomes of surgical treatment). To reduce the potential effects of heterogeneity, we established homogeneous subgroups in terms of treatment setting and used a random effects model for calculations. Furthermore, the analysis was not based on the individual data of the patients but on those published by the investigator; therefore, we could not stratify the data based on these variables. Finally, the average duration of follow-up was relatively short, so we had a small amount of evaluable data on the OS.

One of the strengths of the study was that we only collected data from phase II and phase III RCTs. We were also able to perform statistical analyses in clinically important subgroups. Last but not least, the risk of bias was modest.

For future research, it is important to note that the populations in the studies published so far were significantly younger and fitter, and had fewer comorbidities than ovarian cancer patients in the real world setting. However, the population of the PRIMA [40] study included those who did not carry *BRCA* or HRD mutations and examined patients who represent an even “higher-risk” population compared to the participants of other studies; 35% and 36% of those selected had stage IV OC in the niraparib and placebo groups, and 67% received neo-adjuvant chemotherapy, yet a 38% reduction in hazard of recurrence or death in the overall population was achieved, and 57% in the HRD group. Therefore, the tolerability of therapy may be different in the average clinical setting from that observed in RCTs. Different PARPis may appear to produce different results based on efficacy as well as safety profile. Attempts have already been made [61] to compare the members of this class of medications, but additional research is necessary. Olaparib was used in seven of the clinical trials so far, niraparib in four, rucaparib in two, veliparib in two, and fuzuloparib in one. These data highlight the fact that previous meta-analyses have only been able to compare separate PARPi by pooling inhomogeneous data.

Maintenance PARPi therapy increased the incidence of AEs. Therefore, solutions need to be found to further reduce the number of patients requesting discontinuation of maintenance therapy due to toxicities. Alternatively, PARP therapy may be administered in individual doses, or predictive factors that are more likely to cause severe adverse events in a particular patient should be screened. In the field of toxicities, the long-term effects are also controversial. Concerns may exist regarding the development of secondary malignancies, with MDS or AML being the primary emphasis in this regard [60]. PARPis appear capable of extending PFS, but nevertheless, the majority of tumors will eventually progress. Mapping and comprehending the processes underlying PARPi resistance might be a significant scientific challenge. The repeatability of PARP treatment and the number of possible repetitions are also open questions, closely related to the prevention of resistance development. A deeper understanding of the mechanisms may enable us to supplement the PARPi maintenance with potentiated therapies and may prevent the development of resistance [62, 63]. 

In conclusion, PARPi therapy outperformed control groups in treating advanced OC in different settings in the PFS projection. However, the risk of occurrence of different high-grade AEs also appears to increase. The assessment of BRCA and HRD status may play an essential role in treating patients with PARPi. Maintenance treatment is associated with a significant benefit for the vast majority of patients in the presence of these deficiencies. However, no conclusions can be drawn regarding overall survival on the basis of the currently available data. Our meta-analysis could guide doctors in making day-to-day decisions, selecting patients, and establishing the most appropriate therapy regimen.

### Supplementary Information

**Additional file 1:** **Table S1. **PRISMA 2020 checklist. **Table S2.** Summary of findings & quality of evidence – PFS in recurrent OC: PARPi maintenance vs. placebo. **Table S3.** Summary of findings & quality of evidence – PFS in recurrent OC: PARPi monotherapy vs. chemotherapy. **Table S4.** Summary of findings & quality of evidence – PFS in newly-diagnosed OC: PARPi maintenance vs. placebo. **Table S5.** Summary of findings & quality of evidence – AEs in recurrent OC: PARPi maintenance vs. placebo. **Table S6.** Summary of findings & quality of evidence – AEs in recurrent OC: PARPi monotherapy vs. chemotherapy. **Table S7.** Summary of findings & quality of evidence – AEs in newly-diagnosed OC: PARPi maintenance vs. placebo. **Appendix S1. **The search terms applied in a systematic search. **Figure S1.** Forest plot representing that the PARPi maintenance therapy for recurrent ovarian cancer decrease the hazard ratio for disease progression or death versus placebo in the total population. **Figure S2.** Forest plot representing that the PARPi maintenance therapy for recurrent ovarian cancer decrease the hazard ratio for disease progression or death versus placebo in the BRCAm population. **Figure S3.** Forest plot representing that the PARPi maintenance therapy for recurrent ovarian cancer decrease the hazard ratio for disease progression or death versus placebo in the gBRCAm population. **Figure S4.** Forest plot representing that the PARPi maintenance therapy for recurrent ovarian cancer decrease the hazard ratio for disease progression or death versus placebo in the BRCAw population. **Figure S5.** Forest plot representing that the PARPi monotherapy for recurrent ovarian cancer decrease the hazard ratio for disease progression or death versus chemotherapy in the total population. **Figure S6.** Forest plot representing that the PARPi monotherapy for recurrent ovarian cancer decrease the hazard ratio for disease progression or death versus chemotherapy in the BRCAm population. **Figure S7.** Forest plot representing that the PARPi maintenance therapy for newly-diagnosed ovarian cancer decrease the hazard ratio for disease progression or death versus placebo in the total population. **Figure S8.** Forest plot representing that the PARPi maintenance therapy for newly-diagnosed ovarian cancer decrease the hazard ratio for disease progression or death versus placebo in the BRCAm population. **Figure S9.** Forest plot representing that the PARPi maintenance therapy for recurrent ovarian cancer has little to no effect on the risk of adverse events by any grade versus placebo. **Figure S10.** Forest plot representing that the PARPi maintenance therapy for recurrent ovarian cancer increase the risk of grade 3≤ adverse events versus placebo. **Figure S11.** Forest plot representing that the PARPi maintenance therapy for recurrent ovarian cancer increase the risk of serious adverse events versus placebo. **Figure S12.** Forest plot representing that the PARPi maintenance therapy for recurrent ovarian cancer increase the risk of anaemia by any grade versus placebo. **Figure S13.** Forest plot representing that the PARPi maintenance therapy for recurrent ovarian cancer increase the risk of grade 3≤ anaemia versus placebo. **Figure S14.** Forest plot representing that the PARPi maintenance therapy for recurrent ovarian cancer increase the risk of thrombocytopenia by any grade versus placebo. **Figure S15.** Forest plot representing that the PARPi maintenance therapy for recurrent ovarian cancer increase the risk of grade 3≤ thrombocytopenia versus placebo. **Figure S16.** Forest plot representing that the PARPi maintenance therapy for recurrent ovarian cancer has little to no effect on the risk of leukopenia by any grade versus placebo. **Figure S17.** Forest plot representing that the PARPi maintenance therapy for recurrent ovarian cancer increase the risk of grade 3≤ leukopenia versus placebo. **Figure S18.** Forest plot representing that the PARPi maintenance therapy for recurrent ovarian cancer increase the risk of neutropenia by any grade versus placebo. **Figure S19.** Forest plot representing that the PARPi maintenance therapy for recurrent ovarian cancer increase the risk of grade 3≤ neutropenia versus placebo. **Figure S20.** Forest plot representing that the PARPi maintenance therapy for recurrent ovarian cancer increase the risk of nausea by any grade versus placebo. **Figure S21.** Forest plot representing that the PARPi maintenance therapy for recurrent ovarian cancer increase the risk of grade 3≤ nausea versus placebo. **Figure S22.** Forest plot representing that the PARPi maintenance therapy for recurrent ovarian cancer increase the risk of fatigue by any grade versus placebo. **Figure S23.** Forest plot representing that the PARPi maintenance therapy for recurrent ovarian cancer increase the risk of grade 3≤ fatigue versus placebo. **Figure S24.** Forest plot representing that the PARPi maintenance therapy for recurrent ovarian cancer increase the risk of vomiting by any grade versus placebo. **Figure S25.** Forest plot representing that the PARPi maintenance therapy for recurrent ovarian cancer increase the risk of grade 3≤ vomiting versus placebo. **Figure S26.** Forest plot representing that the PARPi maintenance therapy for recurrent ovarian cancer increase the risk of dose modification versus placebo. **Figure S27.** Forest plot representing that the PARPi maintenance therapy for recurrent ovarian cancer increase the risk of treatment interruption versus placebo. **Figure S28.** Forest plot representing that the PARPi maintenance therapy for recurrent ovarian cancer increase the risk of treatment discontinuation versus placebo. **Figure S29.** Forest plot representing that the PARPi maintenance therapy for recurrent ovarian cancer increase the risk of MDS / AML versus placebo. **Figure S30.** Forest plot representing that the PARPi monotherapy for recurrent ovarian cancer has little to no effect on the risk of anaemia by any grade versus chemotherapy. **Figure S31.** Forest plot representing that the PARPi monotherapy for recurrent ovarian cancer increase the risk of grade 3≤ anaemia versus placebo. **Figure S32.** Forest plot representing that the PARPi monotherapy for recurrent ovarian cancer has little to no effect on the risk of thrombocytopenia by any grade versus chemotherapy. **Figure S33.** Forest plot representing that the PARPi monotherapy for recurrent ovarian cancer has little to no effect on the risk of grade 3≤ thrombocytopenia versus chemotherapy. **Figure S34.** Forest plot representing that the PARPi monotherapy for recurrent ovarian cancer decrease the risk of neutropenia by any grade versus chemotherapy. **Figure S35.** Forest plot representing that the PARPi monotherapy for recurrent ovarian cancer has little to no effect on the risk of grade 3≤ neutropenia versus chemotherapy. **Figure S36.** Forest plot representing that the PARPi monotherapy for recurrent ovarian cancer increase the risk of nausea by any grade versus chemotherapy. **Figure S37.** Forest plot representing that the PARPi monotherapy for recurrent ovarian cancer has little to no effect on the risk of grade 3≤ nausea versus chemotherapy. **Figure S38.** Forest plot representing that the PARPi monotherapy for recurrent ovarian cancer increase the risk of fatigue by any grade versus chemotherapy. **Figure S39.** Forest plot representing that the PARPi monotherapy for recurrent ovarian cancer increase the risk of grade 3≤ fatigue versus chemotherapy. **Figure S40.** Forest plot representing that the PARPi monotherapy for recurrent ovarian cancer increase the risk of vomiting by any grade versus chemotherapy. **Figure S41.** Forest plot representing that the PARPi monotherapy for recurrent ovarian cancer has little to no effect on the risk of grade 3≤ vomiting versus chemotherapy. **Figure S42.** Forest plot representing that the PARPi monotherapy for recurrent ovarian cancer has little to no effect on the risk of dose modification versus chemotherapy**. Figure S43.** Forest plot representing that the PARPi monotherapy for recurrent ovarian cancer has little to no effect on the risk of MDS / AML versus chemotherapy. **Figure S44.** Forest plot representing that the PARPi maintenance therapy for newly-diagnosed ovarian cancer has little to no effect on the risk of adverse event by any grade versus placebo. **Figure S45.** Forest plot representing that the PARPi maintenance therapy for newly-diagnosed ovarian cancer increase the risk of grade 3≤ adverse events versus placebo. **Figure S46.** Forest plot representing that the PARPi maintenance therapy for newly-diagnosed ovarian cancer increase the risk of serious adverse events versus placebo. **Figure S47.** Forest plot representing that the PARPi maintenance therapy for newly-diagnosed ovarian cancer increase the risk of anaemia by any grade versus placebo. **Figure S48.** Forest plot representing that the PARPi maintenance therapy for newly-diagnosed ovarian cancer increase the risk of grade 3≤ anaemia versus placebo. **Figure S49.** Forest plot representing that the PARPi maintenance therapy for newly-diagnosed ovarian cancer increase the risk of thrombocytopenia by any grade versus placebo. **Figure S50.** Forest plot representing that the PARPi maintenance therapy for newly-diagnosed ovarian cancer has little to no effect on the risk of grade 3≤ thrombocytopenia versus placebo. **Figure S51.** Forest plot representing that the PARPi maintenance therapy for newly-diagnosed ovarian cancer increase the risk of neutropenia by any grade versus placebo. **Figure S52.** Forest plot representing that the PARPi maintenance therapy for newly-diagnosed ovarian cancer increase the risk of grade 3≤ neutropenia versus placebo. **Figure S53.** Forest plot representing that the PARPi maintenance therapy for newly-diagnosed ovarian cancer increase the risk of nausea by any grade versus placebo. **Figure S54.** Forest plot representing that the PARPi maintenance therapy for newly-diagnosed ovarian cancer has little to no effect on the risk of grade 3≤ nausea versus placebo. **Figure S55.** Forest plot representing that the PARPi maintenance therapy for newly-diagnosed ovarian cancer increase the risk of fatigue by any grade versus placebo. **Figure S56.** Forest plot representing that the PARPi maintenance therapy for newly-diagnosed ovarian cancer has little to no effect on the risk of grade 3≤ fatigue versus placebo. **Figure S57.** Forest plot representing that the PARPi maintenance therapy for newly-diagnosed ovarian cancer increase the risk of vomiting by any grade versus placebo. **Figure S58.** Forest plot representing that the PARPi maintenance therapy for newly-diagnosed ovarian cancer has little to no effect on the risk of grade 3≤ vomiting versus placebo. **Figure S59.** Forest plot representing that the PARPi maintenance therapy for newly-diagnosed ovarian cancer increase the risk of dose modificaion versus placebo. **Figure S60.** Forest plot representing that the PARPi maintenance therapy for newly-diagnosed ovarian cancer increase the risk of treatmenet interruption versus placebo. **Figure S61.** Forest plot representing that the PARPi maintenance therapy for newly-diagnosed ovarian cancer increase the risk of treatmenet discontinuation versus placebo. **Figure S62.** Forest plot representing that the PARPi maintenance therapy for newly-diagnosed ovarian cancer has little to no effect on the risk of MDS / AML versus placebo. **Figure S63.** Risk of bias summary at study level: for each included trial.

## Data Availability

All of the data that was generated or analyzed during the course of the investigation is included in the published article and the supplementary material.
