# Supplementary material for: PARP inhibitor era in ovarian cancer treatment: a systematic review and meta-analysis of randomized controlled trials
Source: J Ovarian Res. 2024 Feb 26;17:53. doi: 10.1186/s13048-024-01362-y (PMC10895809; doi:10.1186/s13048-024-01362-y)
Supplement: Supplementary file 1 — Additional file 1: Table S1. PRISMA 2020 checklist. Table S2. Summary of findings & quality of evidence – PFS in recurrent OC: PARPi maintenance vs. placebo. Table S3. Summary of findings & quality of evidence – PFS in recurrent OC: PARPi monotherapy vs. chemotherapy. Table S4. Summary of findings & quality of evidence – PFS in newly-diagnosed OC: PARPi maintenance vs. placebo. Table S5. Summary of findings & quality of evidence – AEs in recurrent OC: PARPi maintenance vs. placebo. Table S6. Summary of findings & quality of evidence – AEs in recurrent OC: PARPi monotherapy vs. chemotherapy. Table S7. Summary of findings & quality of evidence – AEs in newly-diagnosed OC: PARPi maintenance vs. placebo. Appendix S1. The search terms applied in a systematic search. Figure S1. Forest plot representing that the PARPi maintenance therapy for recurrent ovarian cancer decrease the hazard ratio for disease progression or death versus placebo in the total population. Figure S2. Forest plot representing that the PARPi maintenance therapy for recurrent ovarian cancer decrease the hazard ratio for disease progression or death versus placebo in the BRCAm population. Figure S3. Forest plot representing that the PARPi maintenance therapy for recurrent ovarian cancer decrease the hazard ratio for disease progression or death versus placebo in the gBRCAm population. Figure S4. Forest plot representing that the PARPi maintenance therapy for recurrent ovarian cancer decrease the hazard ratio for disease progression or death versus placebo in the BRCAw population. Figure S5. Forest plot representing that the PARPi monotherapy for recurrent ovarian cancer decrease the hazard ratio for disease progression or death versus chemotherapy in the total population. Figure S6. Forest plot representing that the PARPi monotherapy for recurrent ovarian cancer decrease the hazard ratio for disease progression or death versus chemotherapy in the BRCAm population. Figure S7. Forest plot representing tha [file 13048_2024_1362_MOESM1_ESM.docx]

**PARP inhibitor era in ovarian cancer treatment: a systematic review and meta-analysis of randomized controlled trials**

**Authors**

István Baradács^1,2^, Brigitta Teutsch^2,3^, Alex Váradi^3^, Alexandra Bilá ^2,6^, Ádám Vincze^2,6^, Péter Hegyi^2,3,4^, Tamás Fazekas^2,5^, Balázs Komoróczy^1,2^, Péter Nyirády^2,5^, Nándor Ács^1,2^, Ferenc Bánhidy^1,2^, Balázs Lintner^1,2^

**Affiliations:**

1. Department of Obstetrics & Gynecology, Semmelweis University, Budapest
2. Centre for Translational Medicine, Semmelweis University, Budapest, Hungary
3. Institute for Translational Medicine, Szentágothai Research Centre, Medical School, University of Pécs, Pécs, Hungary
4. Division of Pancreatic Diseases, Heart and Vascular Center, Semmelweis University, Budapest, Hungary (Péter)
5. Department of Urology, Semmelweis University, Budapest, Hungary
6. School of Medicine, Semmelweis University, Budapest, Hungary

**Corresponding author**

Balázs Lintner MD, PhD

Department of Obstetrics and Gynecology

Semmelweis University School of Medicine

Postal address: H-1082 Budapest, Üllői út 78/A, Hungary

Tel.: +36 20 919 3312

E-mail address: [lintnerster@gmail.](mailto:lintnerster@gmail.)com

**Tables**

**Table S1** PRISMA 2020 checklist

**Table S2** Summary of findings & quality of evidence – PFS in recurrent OC: PARPi maintenance vs. placebo

**Table S3** Summary of findings & quality of evidence – PFS in recurrent OC: PARPi monotherapy vs. chemotherapy

**Table S4** Summary of findings & quality of evidence – PFS in newly-diagnosed OC: PARPi maintenance vs. placebo

**Table S5** Summary of findings & quality of evidence – AEs in recurrent OC: PARPi maintenance vs. placebo

**Table S6** Summary of findings & quality of evidence – AEs in recurrent OC: PARPi monotherapy vs. chemotherapy

**Table S7** Summary of findings & quality of evidence – AEs in newly-diagnosed OC: PARPi maintenance vs. placebo

**Appendix**

**Appendix S1** The search terms applied in a systematic search

**Figure legends**

**Figure S1** Forest plot representing that the PARPi maintenance therapy for recurrent ovarian cancer decrease the hazard ratio for disease progression or death versus placebo in the total population

**Figure S2** Forest plot representing that the PARPi maintenance therapy for recurrent ovarian cancer decrease the hazard ratio for disease progression or death versus placebo in the BRCAm population

**Figure S3** Forest plot representing that the PARPi maintenance therapy for recurrent ovarian cancer decrease the hazard ratio for disease progression or death versus placebo in the gBRCAm population

**Figure S4** Forest plot representing that the PARPi maintenance therapy for recurrent ovarian cancer decrease the hazard ratio for disease progression or death versus placebo in the BRCAw population

**Figure S5** Forest plot representing that the PARPi monotherapy for recurrent ovarian cancer decrease the hazard ratio for disease progression or death versus chemotherapy in the total population

**Figure S6** Forest plot representing that the PARPi monotherapy for recurrent ovarian cancer decrease the hazard ratio for disease progression or death versus chemotherapy in the BRCAm population

**Figure S7** Forest plot representing that the PARPi maintenance therapy for newly-diagnosed ovarian cancer decrease the hazard ratio for disease progression or death versus placebo in the total population

**Figure S8** Forest plot representing that the PARPi maintenance therapy for newly-diagnosed ovarian cancer decrease the hazard ratio for disease progression or death versus placebo in the BRCAm population

**Figure S9** Forest plot representing that the PARPi maintenance therapy for recurrent ovarian cancer has little to no effect on the risk of adverse events by any grade versus placebo

**Figure S10** Forest plot representing that the PARPi maintenance therapy for recurrent ovarian cancer increase the risk of grade 3≤ adverse events versus placebo

**Figure S11** Forest plot representing that the PARPi maintenance therapy for recurrent ovarian cancer increase the risk of serious adverse events versus placebo

**Figure S12** Forest plot representing that the PARPi maintenance therapy for recurrent ovarian cancer increase the risk of anaemia by any grade versus placebo

**Figure S13** Forest plot representing that the PARPi maintenance therapy for recurrent ovarian cancer increase the risk of grade 3≤ anaemia versus placebo

**Figure S14** Forest plot representing that the PARPi maintenance therapy for recurrent ovarian cancer increase the risk of thrombocytopenia by any grade versus placebo

**Figure S15** Forest plot representing that the PARPi maintenance therapy for recurrent ovarian cancer increase the risk of grade 3≤ thrombocytopenia versus placebo

**Figure S16** Forest plot representing that the PARPi maintenance therapy for recurrent ovarian cancer has little to no effect on the risk of leukopenia by any grade versus placebo

**Figure S17** Forest plot representing that the PARPi maintenance therapy for recurrent ovarian cancer increase the risk of grade 3≤ leukopenia versus placebo

**Figure S18** Forest plot representing that the PARPi maintenance therapy for recurrent ovarian cancer increase the risk of neutropenia by any grade versus placebo

**Figure S19** Forest plot representing that the PARPi maintenance therapy for recurrent ovarian cancer increase the risk of grade 3≤ neutropenia versus placebo

**Figure S20** Forest plot representing that the PARPi maintenance therapy for recurrent ovarian cancer increase the risk of nausea by any grade versus placebo

**Figure S21** Forest plot representing that the PARPi maintenance therapy for recurrent ovarian cancer increase the risk of grade 3≤ nausea versus placebo

**Figure S22** Forest plot representing that the PARPi maintenance therapy for recurrent ovarian cancer increase the risk of fatigue by any grade versus placebo

**Figure S23** Forest plot representing that the PARPi maintenance therapy for recurrent ovarian cancer increase the risk of grade 3≤ fatigue versus placebo

**Figure S24** Forest plot representing that the PARPi maintenance therapy for recurrent ovarian cancer increase the risk of vomiting by any grade versus placebo

**Figure S25** Forest plot representing that the PARPi maintenance therapy for recurrent ovarian cancer increase the risk of grade 3≤ vomiting versus placebo

**Figure S26** Forest plot representing that the PARPi maintenance therapy for recurrent ovarian cancer increase the risk of dose modification versus placebo

**Figure S27** Forest plot representing that the PARPi maintenance therapy for recurrent ovarian cancer increase the risk of treatment interruption versus placebo

**Figure S28** Forest plot representing that the PARPi maintenance therapy for recurrent ovarian cancer increase the risk of treatment discontinuation versus placebo

**Figure S29** Forest plot representing that the PARPi maintenance therapy for recurrent ovarian cancer increase the risk of MDS / AML versus placebo

**Figure S30** Forest plot representing that the PARPi monotherapy for recurrent ovarian cancer has little to no effect on the risk of anaemia by any grade versus chemotherapy

**Figure S31** Forest plot representing that the PARPi monotherapy for recurrent ovarian cancer increase the risk of grade 3≤ anaemia versus placebo

**Figure S32** Forest plot representing that the PARPi monotherapy for recurrent ovarian cancer has little to no effect on the risk of thrombocytopenia by any grade versus chemotherapy

**Figure S33** Forest plot representing that the PARPi monotherapy for recurrent ovarian cancer has little to no effect on the risk of grade 3≤ thrombocytopenia versus chemotherapy

**Figure S34** Forest plot representing that the PARPi monotherapy for recurrent ovarian cancer decrease the risk of neutropenia by any grade versus chemotherapy

**Figure S35** Forest plot representing that the PARPi monotherapy for recurrent ovarian cancer has little to no effect on the risk of grade 3≤ neutropenia versus chemotherapy

**Figure S36** Forest plot representing that the PARPi monotherapy for recurrent ovarian cancer increase the risk of nausea by any grade versus chemotherapy

**Figure S37** Forest plot representing that the PARPi monotherapy for recurrent ovarian cancer has little to no effect on the risk of grade 3≤ nausea versus chemotherapy

**Figure S38** Forest plot representing that the PARPi monotherapy for recurrent ovarian cancer increase the risk of fatigue by any grade versus chemotherapy

**Figure S39** Forest plot representing that the PARPi monotherapy for recurrent ovarian cancer increase the risk of grade 3≤ fatigue versus chemotherapy

**Figure S40** Forest plot representing that the PARPi monotherapy for recurrent ovarian cancer increase the risk of vomiting by any grade versus chemotherapy

**Figure S41** Forest plot representing that the PARPi monotherapy for recurrent ovarian cancer has little to no effect on the risk of grade 3≤ vomiting versus chemotherapy

**Figure S42** Forest plot representing that the PARPi monotherapy for recurrent ovarian cancer has little to no effect on the risk of dose modification versus chemotherapy

**Figure S43** Forest plot representing that the PARPi monotherapy for recurrent ovarian cancer has little to no effect on the risk of MDS / AML versus chemotherapy

**Figure S44** Forest plot representing that the PARPi maintenance therapy for newly-diagnosed ovarian cancer has little to no effect on the risk of adverse event by any grade versus placebo

**Figure S45** Forest plot representing that the PARPi maintenance therapy for newly-diagnosed ovarian cancer increase the risk of grade 3≤ adverse events versus placebo

**Figure S46** Forest plot representing that the PARPi maintenance therapy for newly-diagnosed ovarian cancer increase the risk of serious adverse events versus placebo

**Figure S47** Forest plot representing that the PARPi maintenance therapy for newly-diagnosed ovarian cancer increase the risk of anaemia by any grade versus placebo

**Figure S48** Forest plot representing that the PARPi maintenance therapy for newly-diagnosed ovarian cancer increase the risk of grade 3≤ anaemia versus placebo

**Figure S49** Forest plot representing that the PARPi maintenance therapy for newly-diagnosed ovarian cancer increase the risk of thrombocytopenia by any grade versus placebo

**Figure S50** Forest plot representing that the PARPi maintenance therapy for newly-diagnosed ovarian cancer has little to no effect on the risk of grade 3≤ thrombocytopenia versus placebo

**Figure S51** Forest plot representing that the PARPi maintenance therapy for newly-diagnosed ovarian cancer increase the risk of neutropenia by any grade versus placebo

**Figure S52** Forest plot representing that the PARPi maintenance therapy for newly-diagnosed ovarian cancer increase the risk of grade 3≤ neutropenia versus placebo

**Figure S53** Forest plot representing that the PARPi maintenance therapy for newly-diagnosed ovarian cancer increase the risk of nausea by any grade versus placebo  **Figure S54** Forest plot representing that the PARPi maintenance therapy for newly-diagnosed ovarian cancer has little to no effect on the risk of grade 3≤ nausea versus placebo

**Figure S55** Forest plot representing that the PARPi maintenance therapy for newly-diagnosed ovarian cancer increase the risk of fatigue by any grade versus placebo

**Figure S56** Forest plot representing that the PARPi maintenance therapy for newly-diagnosed ovarian cancer has little to no effect on the risk of grade 3≤ fatigue versus placebo

**Figure S57** Forest plot representing that the PARPi maintenance therapy for newly-diagnosed ovarian cancer increase the risk of vomiting by any grade versus placebo

**Figure S58** Forest plot representing that the PARPi maintenance therapy for newly-diagnosed ovarian cancer has little to no effect on the risk of grade 3≤ vomiting versus placebo

**Figure S59** Forest plot representing that the PARPi maintenance therapy for newly-diagnosed ovarian cancer increase the risk of dose modificaion versus placebo

**Figure S60** Forest plot representing that the PARPi maintenance therapy for newly-diagnosed ovarian cancer increase the risk of treatmenet interruption versus placebo

**Figure S61** Forest plot representing that the PARPi maintenance therapy for newly-diagnosed ovarian cancer increase the risk of treatmenet discontinuation versus placebo

**Figure S62** Forest plot representing that the PARPi maintenance therapy for newly-diagnosed ovarian cancer has little to no effect on the risk of MDS / AML versus placebo

**Figure S63** Risk of bias summary at study level: for each included trial

**Table S1** PRISMA 2020 checklist

| **Section and Topic** | **Item #** | **Checklist item** | **Location where item is reported** |
| --- | --- | --- | --- |
| **TITLE** | | |  |
| Title | 1 | Identify the report as a systematic review. | 1 |
| **ABSTRACT** | | |  |
| Abstract | 2 | See the PRISMA 2020 for Abstracts checklist. | 3 |
| **INTRODUCTION** | | |  |
| Rationale | 3 | Describe the rationale for the review in the context of existing knowledge. | 4 |
| Objectives | 4 | Provide an explicit statement of the objective(s) or question(s) the review addresses. | 4 |
| **METHODS** | | |  |
| Eligibility criteria | 5 | Specify the inclusion and exclusion criteria for the review and how studies were grouped for the syntheses. | 5 |
| Information sources | 6 | Specify all databases, registers, websites, organisations, reference lists and other sources searched or consulted to identify studies. Specify the date when each source was last searched or consulted. | 5 |
| Search strategy | 7 | Present the full search strategies for all databases, registers and websites, including any filters and limits used. | 10  Supplementary material |
| Selection process | 8 | Specify the methods used to decide whether a study met the inclusion criteria of the review, including how many reviewers screened each record and each report retrieved, whether they worked independently, and if applicable, details of automation tools used in the process. | 5 |
| Data collection process | 9 | Specify the methods used to collect data from reports, including how many reviewers collected data from each report, whether they worked independently, any processes for obtaining or confirming data from study investigators, and if applicable, details of automation tools used in the process. | 5 |
| Data items | 10a | List and define all outcomes for which data were sought. Specify whether all results that were compatible with each outcome domain in each study were sought (e.g. for all measures, time points, analyses), and if not, the methods used to decide which results to collect. | 5 |
|  | 10b | List and define all other variables for which data were sought (e.g. participant and intervention characteristics, funding sources). Describe any assumptions made about any missing or unclear information. | 5 |
| Study risk of bias assessment | 11 | Specify the methods used to assess risk of bias in the included studies, including details of the tool(s) used, how many reviewers assessed each study and whether they worked independently, and if applicable, details of automation tools used in the process. | 5-6 |
| Effect measures | 12 | Specify for each outcome the effect measure(s) (e.g. risk ratio, mean difference) used in the synthesis or presentation of results. | 5-6 |
| Synthesis methods | 13a | Describe the processes used to decide which studies were eligible for each synthesis (e.g. tabulating the study intervention characteristics and comparing against the planned groups for each synthesis (item #5)). | 5-6 |
|  | 13b | Describe any methods required to prepare the data for presentation or synthesis, such as handling of missing summary statistics, or data conversions. | 5-6 |
|  | 13c | Describe any methods used to tabulate or visually display results of individual studies and syntheses. | 5-6 |
|  | 13d | Describe any methods used to synthesize results and provide a rationale for the choice(s). If meta-analysis was performed, describe the model(s), method(s) to identify the presence and extent of statistical heterogeneity, and software package(s) used. | 5-6 |
|  | 13e | Describe any methods used to explore possible causes of heterogeneity among study results (e.g. subgroup analysis, meta-regression). | 5-6 |
|  | 13f | Describe any sensitivity analyses conducted to assess robustness of the synthesized results. | 5-6 |
| Reporting bias assessment | 14 | Describe any methods used to assess risk of bias due to missing results in a synthesis (arising from reporting biases). | 5-6 |
| Certainty assessment | 15 | Describe any methods used to assess certainty (or confidence) in the body of evidence for an outcome. | 5-6 |
| **RESULTS** | | |  |
| Study selection | 16a | Describe the results of the search and selection process, from the number of records identified in the search to the number of studies included in the review, ideally using a flow diagram. | 7 |
|  | 16b | Cite studies that might appear to meet the inclusion criteria, but which were excluded, and explain why they were excluded. | 7-8 |
| Study characteristics | 17 | Cite each included study and present its characteristics. | 18-20 |
| Risk of bias in studies | 18 | Present assessments of risk of bias for each included study. | 13; 32 Supplementary material |
| Results of individual studies | 19 | For all outcomes, present, for each study: (a) summary statistics for each group (where appropriate) and (b) an effect estimate and its precision (e.g. confidence/credible interval), ideally using structured tables or plots. | 9-13 |
| Results of syntheses | 20a | For each synthesis, briefly summarise the characteristics and risk of bias among contributing studies. | 9-13 |
|  | 20b | Present results of all statistical syntheses conducted. If meta-analysis was done, present for each the summary estimate and its precision (e.g. confidence/credible interval) and measures of statistical heterogeneity. If comparing groups, describe the direction of the effect. | 9-13 |
|  | 20c | Present results of all investigations of possible causes of heterogeneity among study results. | 9-13 |
|  | 20d | Present results of all sensitivity analyses conducted to assess the robustness of the synthesized results. | 9-13 |
| Reporting biases | 21 | Present assessments of risk of bias due to missing results (arising from reporting biases) for each synthesis assessed. | 32 Supplementary material |
| Certainty of evidence | 22 | Present assessments of certainty (or confidence) in the body of evidence for each outcome assessed. | 13; 33-46 Supplementary material |
| **DISCUSSION** | | |  |
| Discussion | 23a | Provide a general interpretation of the results in the context of other evidence. | 14-16 |
|  | 23b | Discuss any limitations of the evidence included in the review. | 14-16 |
|  | 23c | Discuss any limitations of the review processes used. | 14-16 |
|  | 23d | Discuss implications of the results for practice, policy, and future research. | 14-16 |
| **OTHER INFORMATION** | | |  |
| Registration and protocol | 24a | Provide registration information for the review, including register name and registration number, or state that the review was not registered. | 3 |
|  | 24b | Indicate where the review protocol can be accessed, or state that a protocol was not prepared. | 5 |
|  | 24c | Describe and explain any amendments to information provided at registration or in the protocol. | 5 |
| Support | 25 | Describe sources of financial or non-financial support for the review, and the role of the funders or sponsors in the review. | 1 |
| Competing interests | 26 | Declare any competing interests of review authors. | 1 |
| Availability of data, code and other materials | 27 | Report which of the following are publicly available and where they can be found: template data collection forms; data extracted from included studies; data used for all analyses; analytic code; any other materials used in the review. | 2 |

*From:*  Page MJ, McKenzie JE, Bossuyt PM, Boutron I, Hoffmann TC, Mulrow CD, et al. The PRISMA 2020 statement: an updated guideline for reporting systematic reviews. BMJ 2021;372:n71. doi: 10.1136/bmj.n71

For more information, visit: <http://www.prisma-statement.org/>

**Appendix S1** The search terms applied in a systematic search

**MEDLINE (via PubMed):**

(ovary cancer OR ovary neoplasm OR ovary carcinoma OR ovarian cancer OR ovarian carcinoma OR ovarian neoplasms OR ovary tumor OR tubal cancer OR peritoneal cancer) AND (poly(adp-ribose) polymerase inhibitors OR PARP Inhibitors OR PARP inhibitor OR "PARP inhibitor*" OR "Poly adp-ribose" OR Olaparib OR rucaparib OR niraparib OR veliparib OR talazoparib OR pamiparib OR fluzoparib)

**Cochrane Library (CENTRAL):**

(ovary cancer OR ovary neoplasm OR ovary carcinoma OR ovarian cancer OR ovarian carcinoma OR ovarian neoplasms OR ovary tumor OR tubal cancer OR peritoneal cancer) AND (poly(adp-ribose) polymerase inhibitors OR PARP Inhibitors OR PARP inhibitor OR "PARP inhibitor*" OR "Poly adp-ribose" OR Olaparib OR rucaparib OR niraparib OR veliparib OR talazoparib OR pamiparib OR fluzoparib)

**Embase:**

('ovary cancer' OR 'ovary neoplasm' OR 'ovary carcinoma' OR 'ovarian cancer' OR 'ovarian carcinoma' OR 'ovarian neoplasms' OR 'ovary tumor' OR 'tubal cancer' OR 'peritoneal cancer') AND ('poly(adp-ribose) polymerase inhibitors' OR 'PARP Inhibitors' OR 'PARP Inhibitor' OR 'PARP inhibitor*' OR 'Poly adp-ribose' OR Olaparib OR rucaparib OR niraparib OR veliparib OR talazoparib OR pamiparib OR fluzoparib)

**Figure S1** Forest plot representing that PARPi maintenance therapy for recurrent ovarian cancer increase PFS versus placebo in the total population


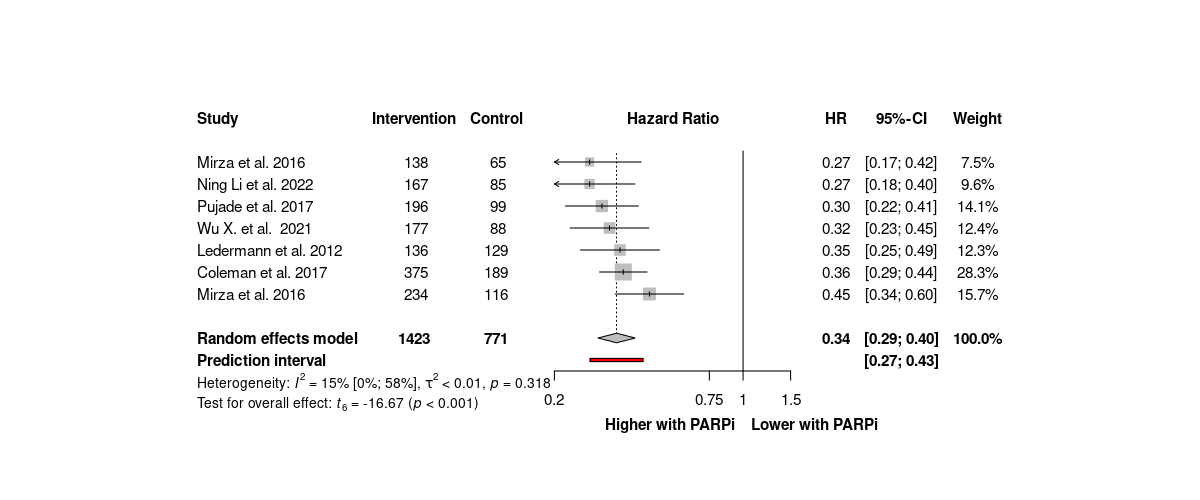


**Figure S2** Forest plot representing that PARPi maintenance therapy for recurrent ovarian cancer increase PFS versus placebo in the BRCAm population


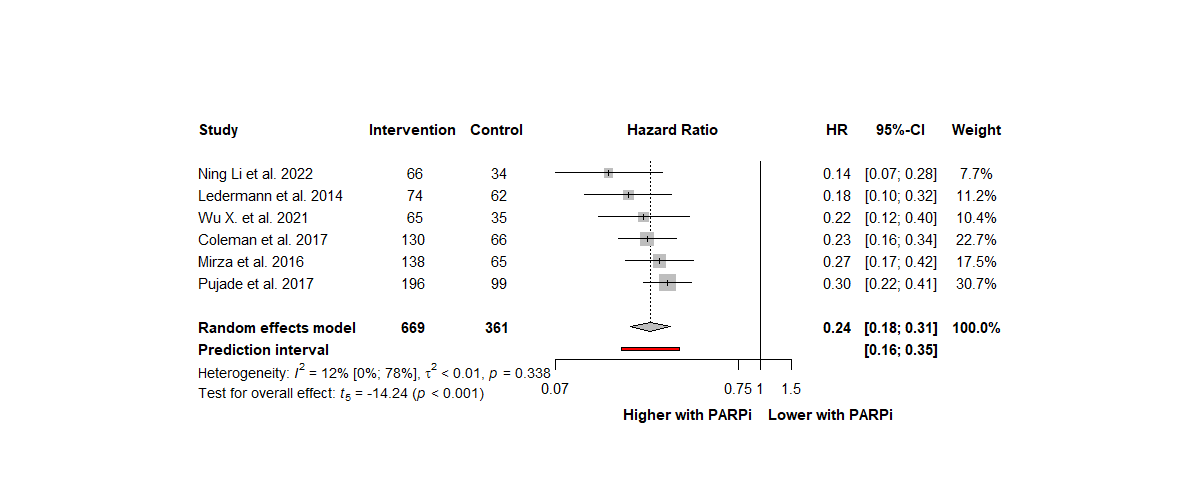


**Figure S3** Forest plot representing that PARPi maintenance therapy for recurrent ovarian cancer increase PFS versus placebo in the gBRCAm population


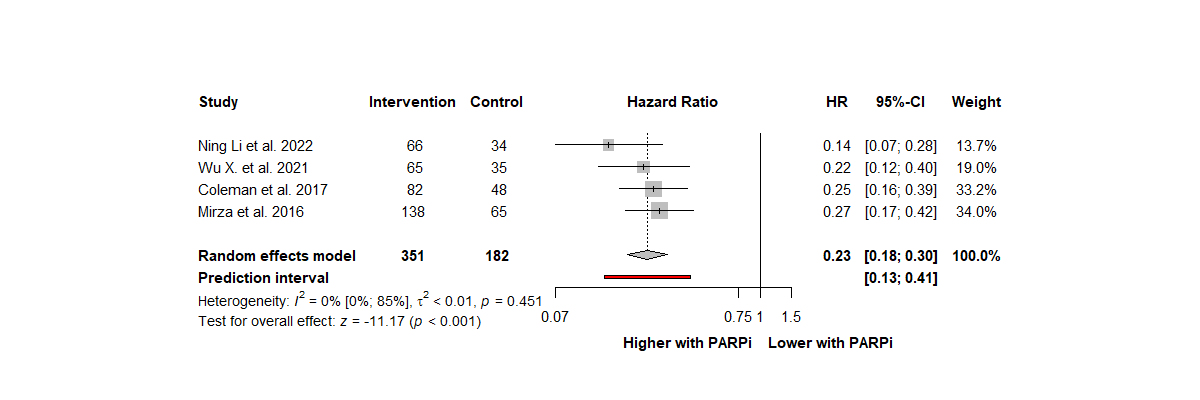


**Figure S4** Forest plot representing that PARPi maintenance therapy for recurrent ovarian cancer increase PFS versus placebo in the BRCAw population


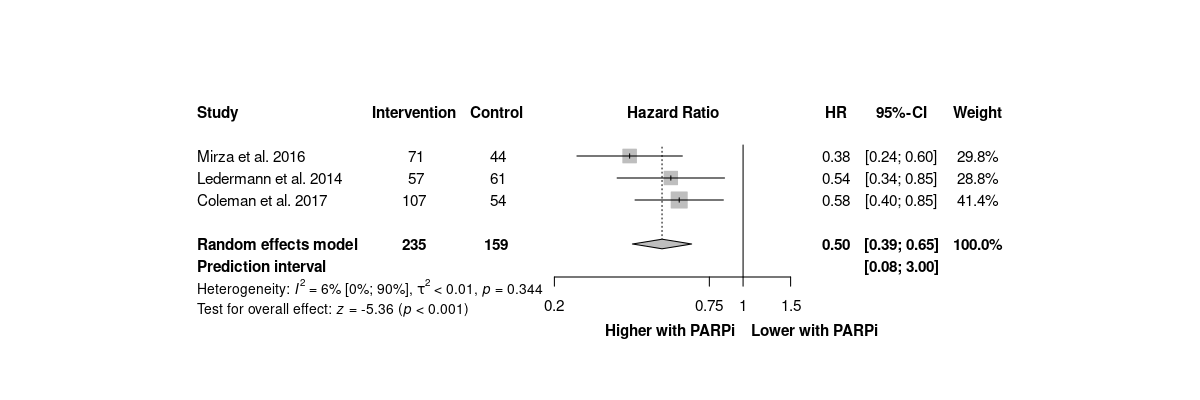


**Figure S5** Forest plot representing that the PARPi monotherapy for recurrent ovarian cancer do not significantly increase PFS versus chemotherapy in the total population


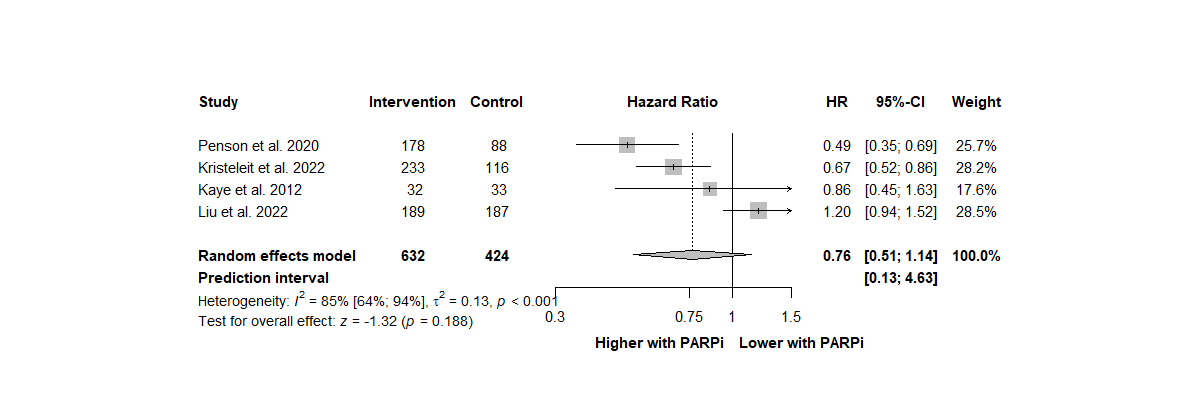


**Figure S6** Forest plot representing that the PARPi monotherapy for recurrent ovarian cancer increase PFS versus chemotherapy in the BRCAm population


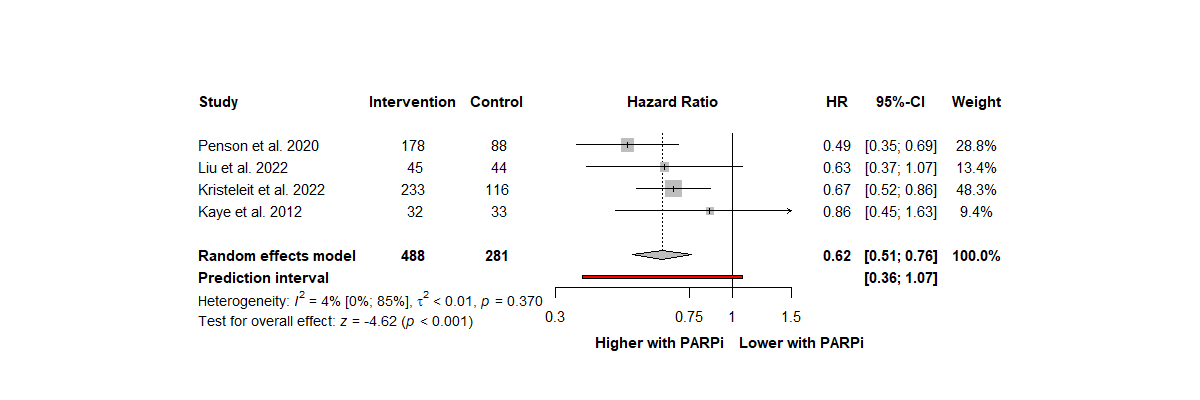


**Figure S7** Forest plot representing that the PARPi maintenance therapy for newly-diagnosed ovarian cancer increase PFS versus placebo in the total population


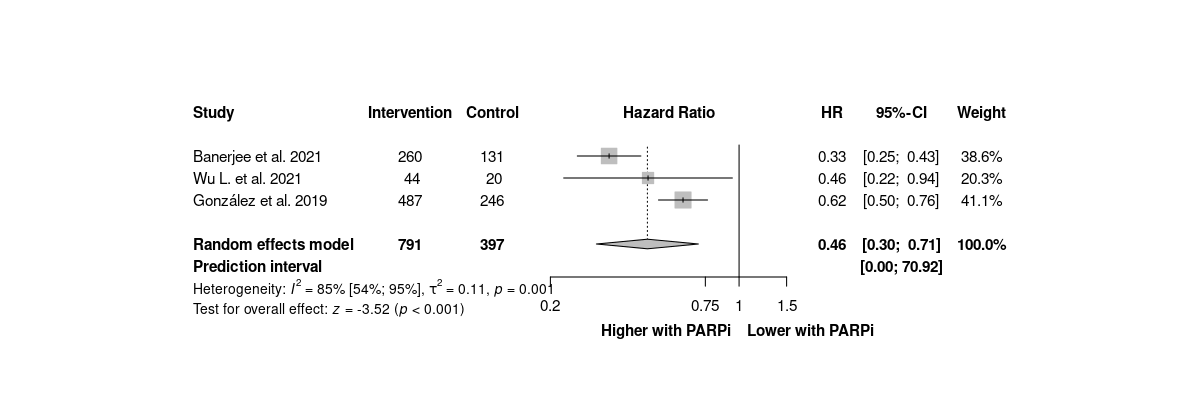


**Figure S8** Forest plot representing that the PARPi maintenance therapy for newly-diagnosed ovarian cancer increase PFS versus placebo in the BRCAm population


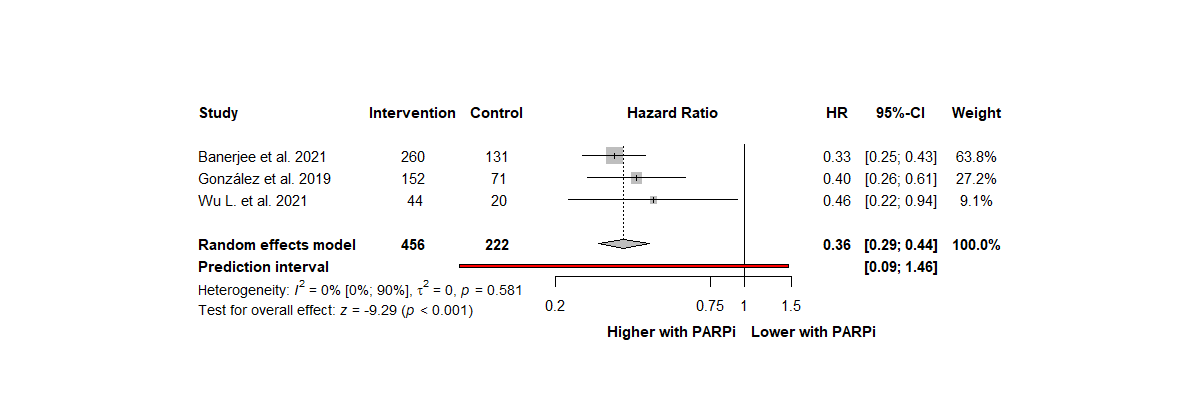


**Figure S9** Forest plot representing that the PARPi maintenance therapy for recurrent ovarian cancer has little to no effect on the risk of adverse event by any grade versus placebo


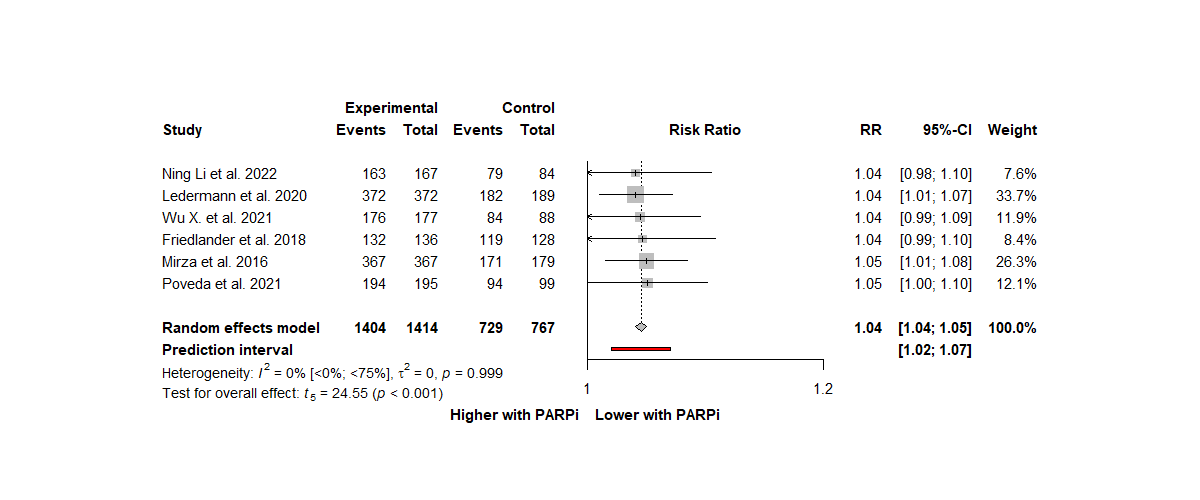


**Figure S10** Forest plot representing that the PARPi maintenance therapy for recurrent ovarian cancer increase the risk of grade 3≤ adverse events versus placebo


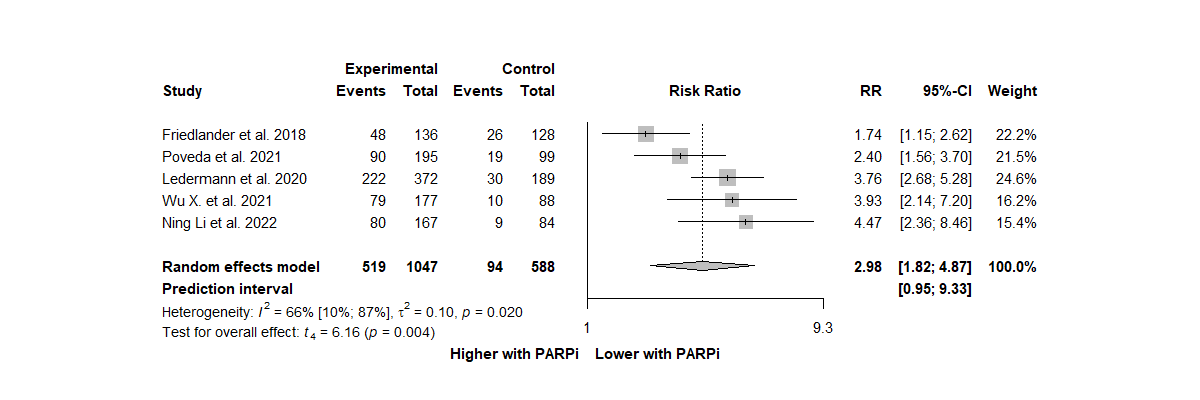


**Figure S11** Forest plot representing that the PARPi maintenance therapy for recurrent ovarian cancer increase the risk of serious adverse events versus placebo


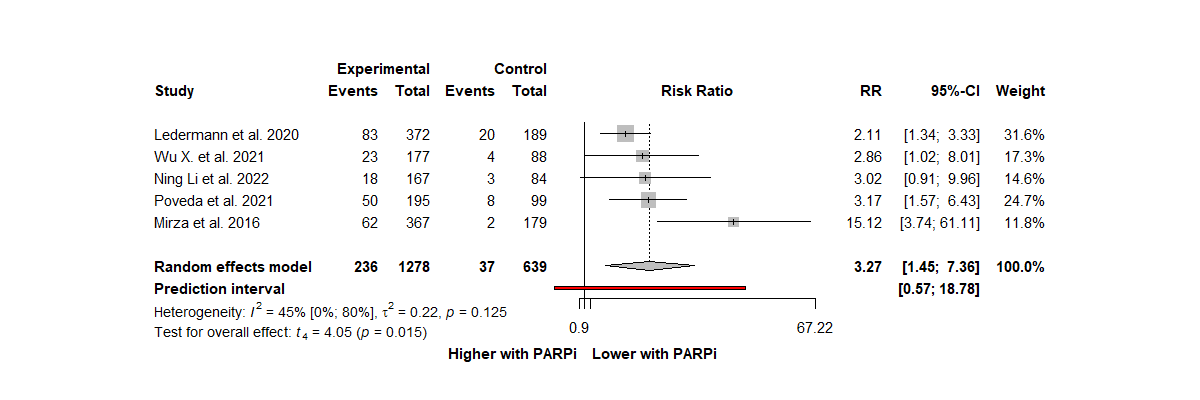


**Figure S12** Forest plot representing that the PARPi maintenance therapy for recurrent ovarian cancer increase the risk of anaemia by any grade versus placebo


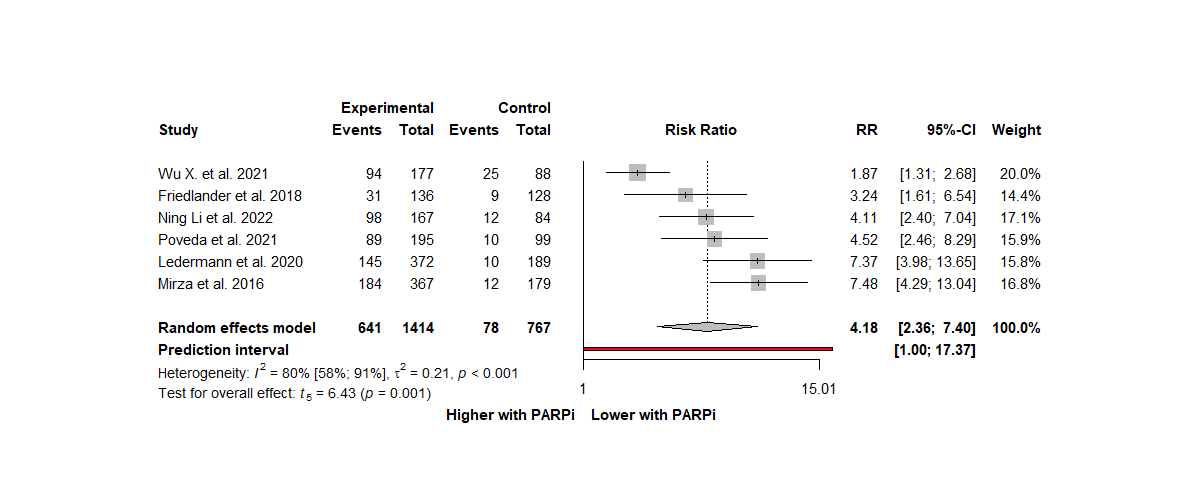


**Figure S13** Forest plot representing that the PARPi maintenance therapy for recurrent ovarian cancer increase the risk of grade 3≤ anaemia versus placebo


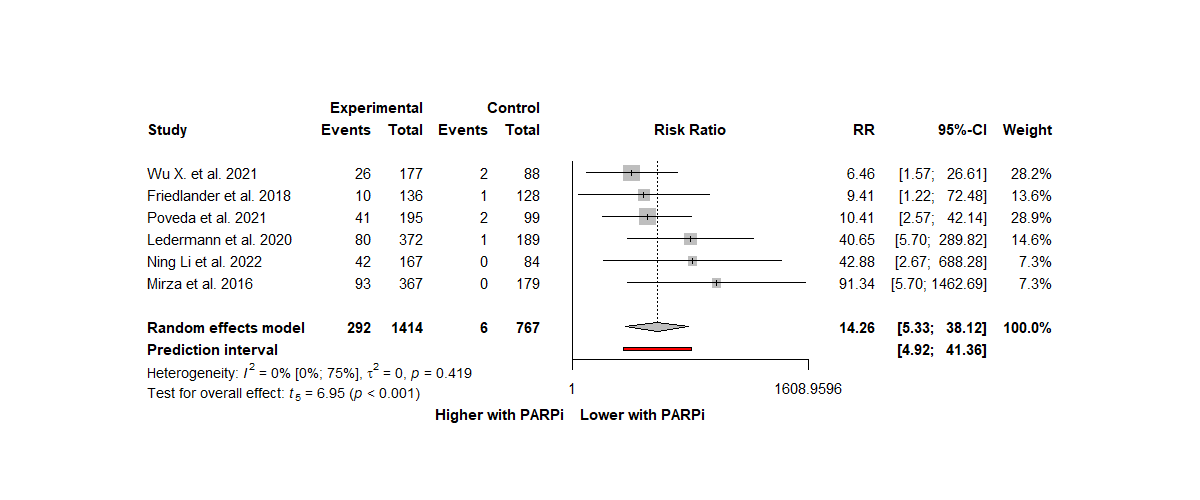


**Figure S14** Forest plot representing that the PARPi maintenance therapy for recurrent ovarian cancer increase the risk of thrombocytopenia by any grade versus placebo


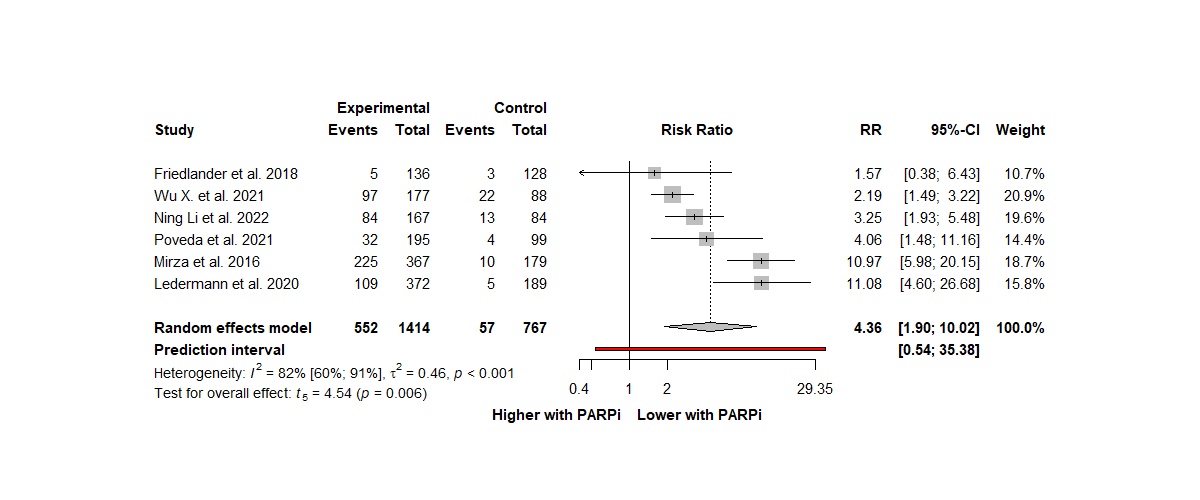


**Figure S15** Forest plot representing that the PARPi maintenance therapy for recurrent ovarian cancer increase the risk of grade 3≤ thrombocytopenia versus placebo


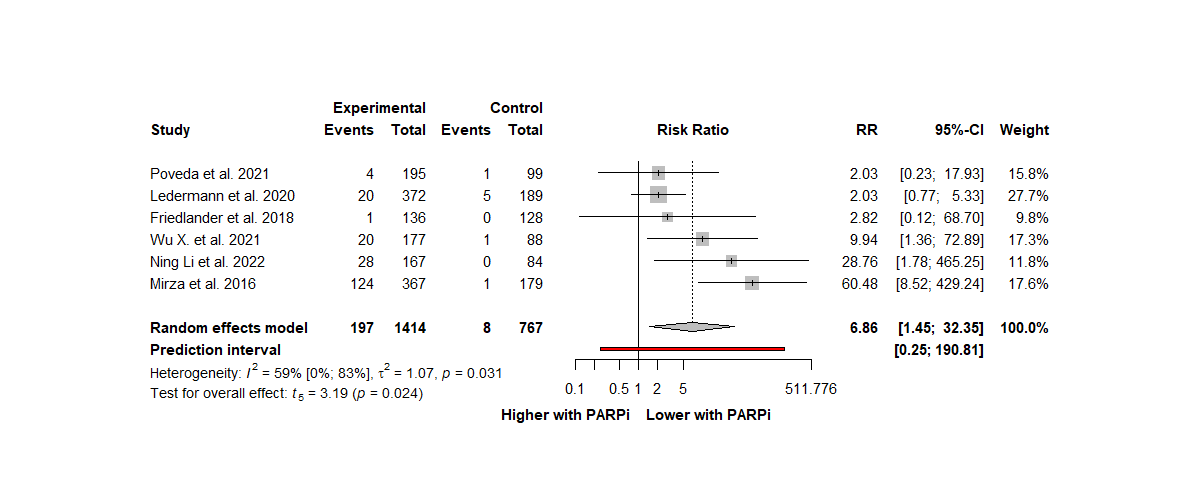


**Figure S16** Forest plot representing that the PARPi maintenance therapy for recurrent ovarian cancer has little to no effect on the risk of leukopenia by any grade versus placebo


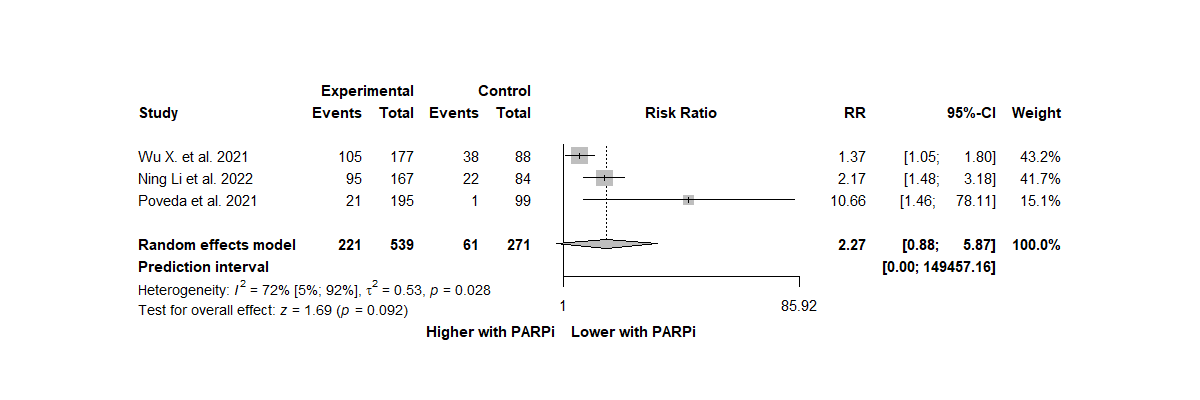


**Figure S17** Forest plot representing that the PARPi maintenance therapy for recurrent ovarian cancer increase the risk of grade 3≤ leukopenia versus placebo


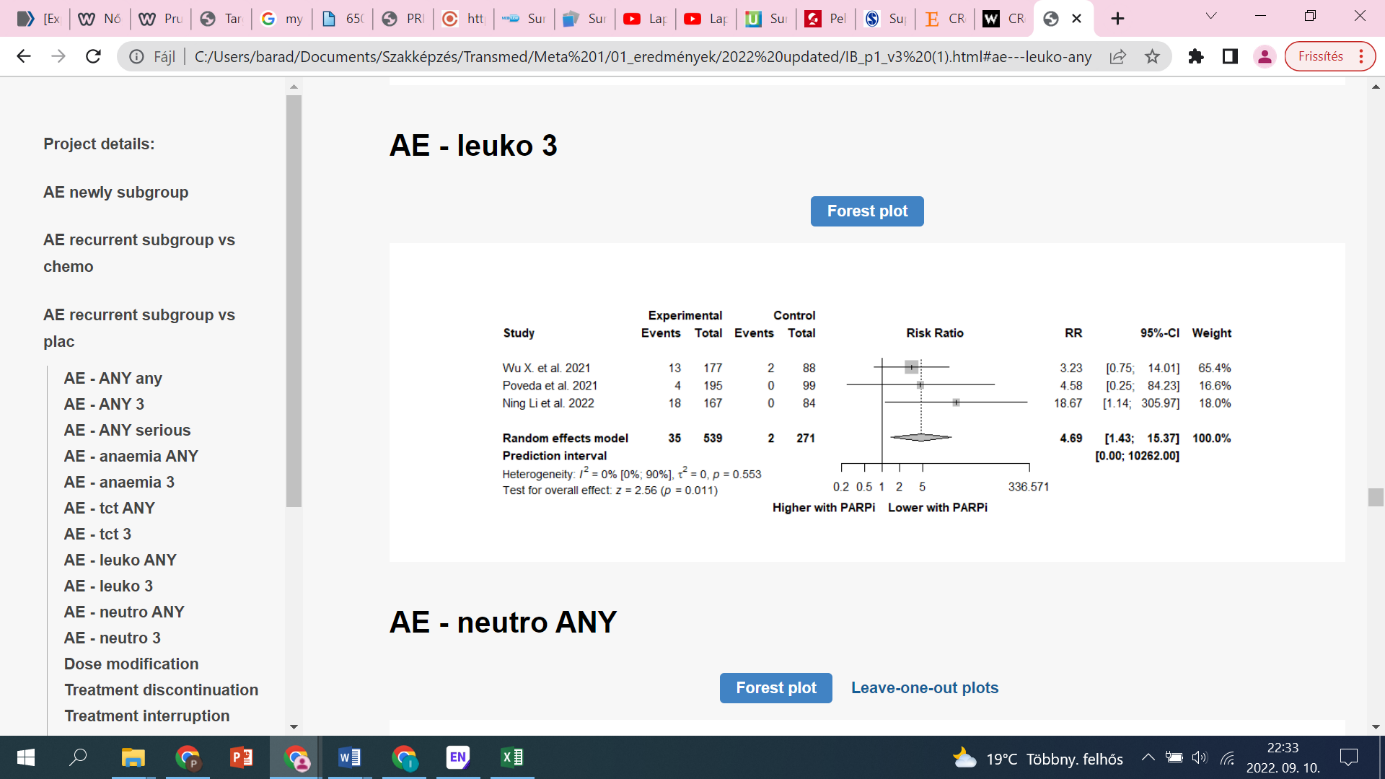


**Figure S18** Forest plot representing that the PARPi maintenance therapy for recurrent ovarian cancer increase the risk of neutropenia by any grade versus placebo


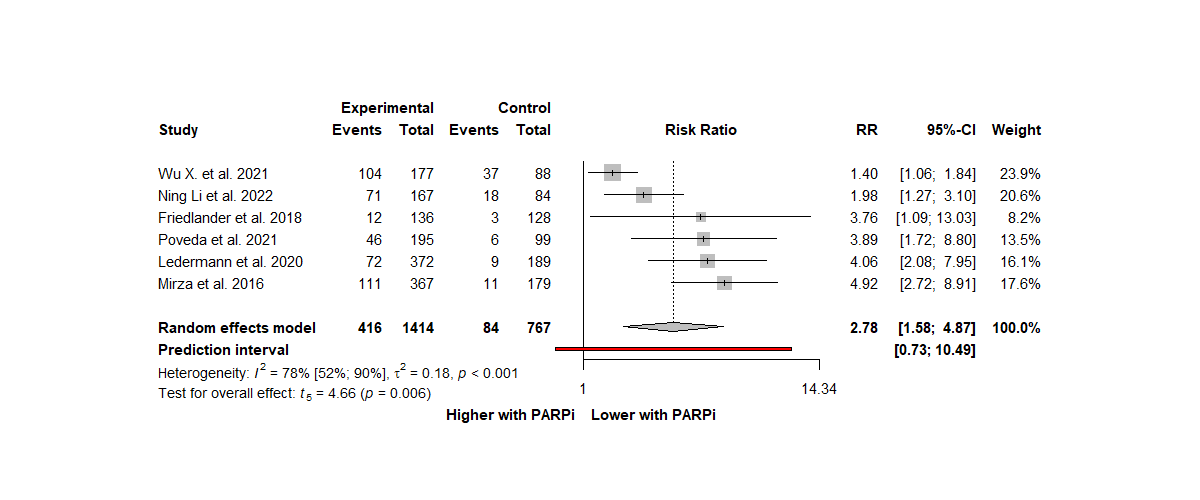


**Figure S19** Forest plot representing that the PARPi maintenance therapy for recurrent ovarian cancer increase the risk of grade 3≤ neutropenia versus placebo


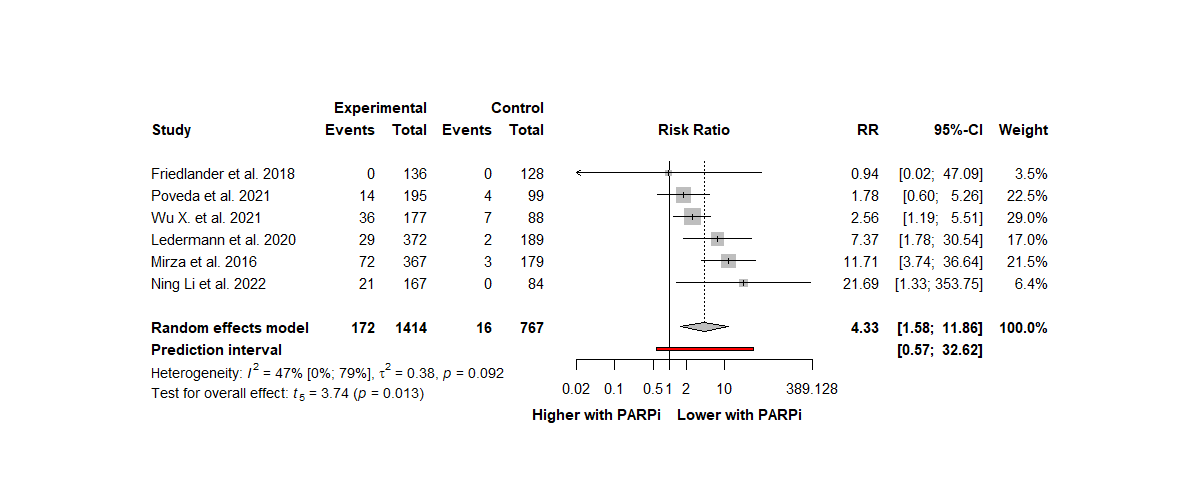


**Figure S20** Forest plot representing that the PARPi maintenance therapy for recurrent ovarian cancer increase the risk of nausea by any grade versus placebo


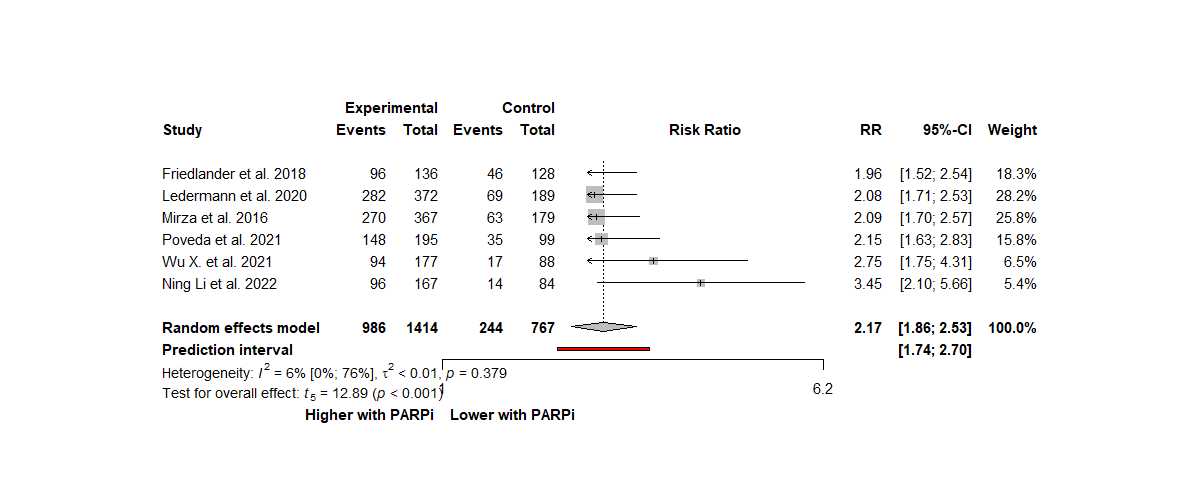


**Figure S21** Forest plot representing that the PARPi maintenance therapy for recurrent ovarian cancer increase the risk of grade 3≤ nausea versus placebo


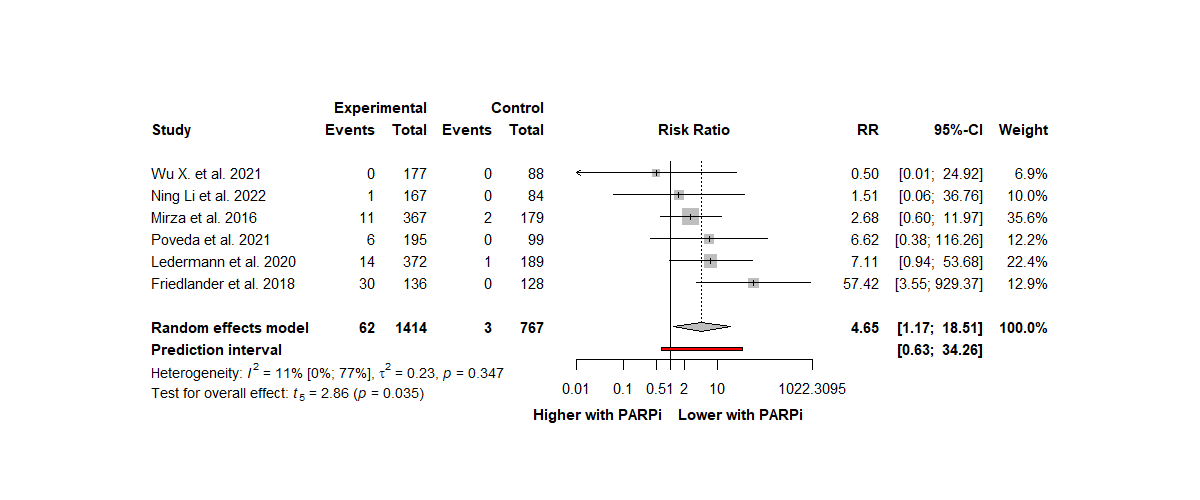


**Figure S22** Forest plot representing that the PARPi maintenance therapy for recurrent ovarian cancer increase the risk of fatigue by any grade versus placebo


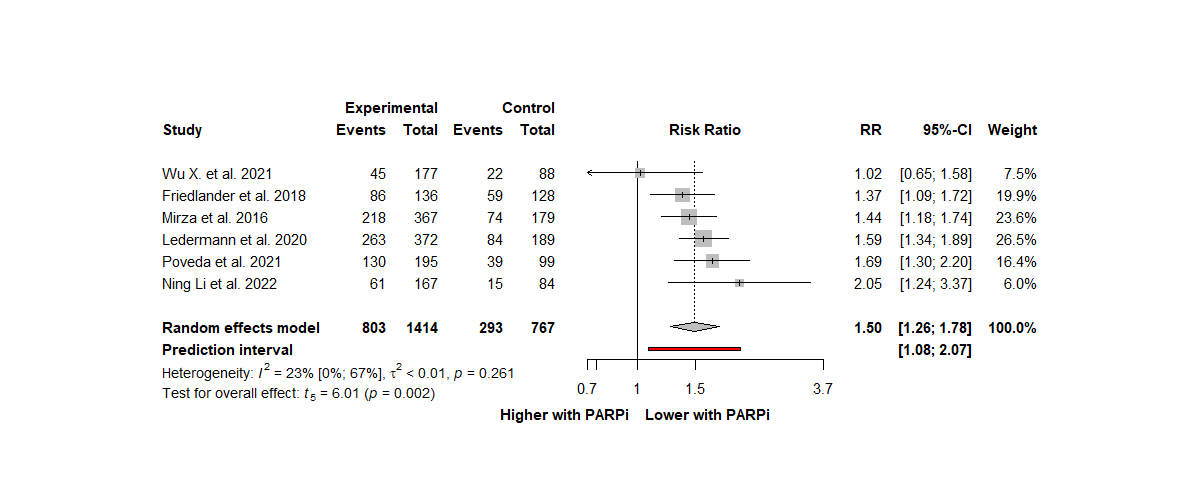
 **Figure S23** Forest plot representing that the PARPi maintenance therapy for recurrent ovarian cancer increase the risk of grade 3≤ fatigue versus placebo


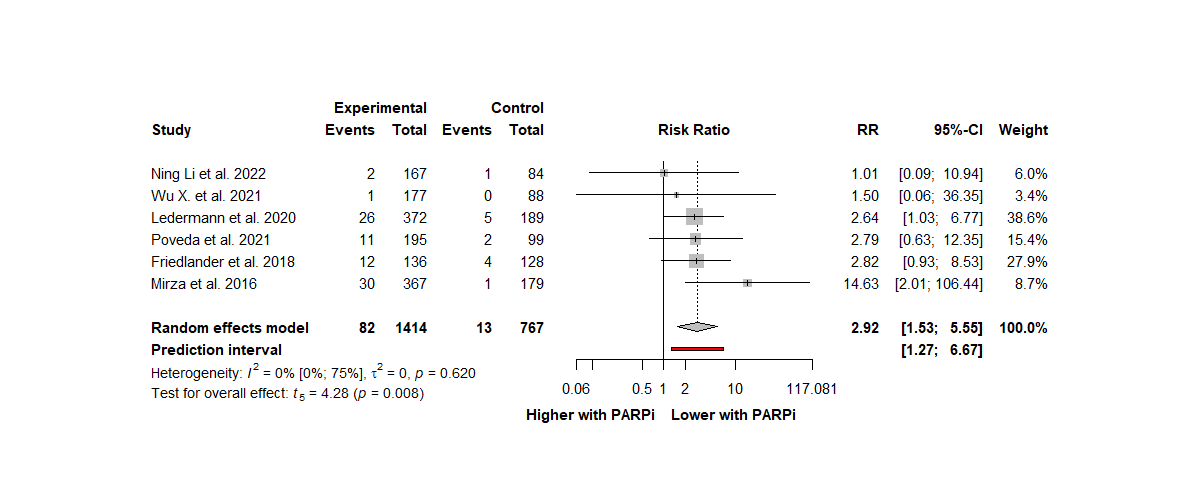


**Figure S24** Forest plot representing that the PARPi maintenance therapy for recurrent ovarian cancer increase the risk of vomiting by any grade versus placebo


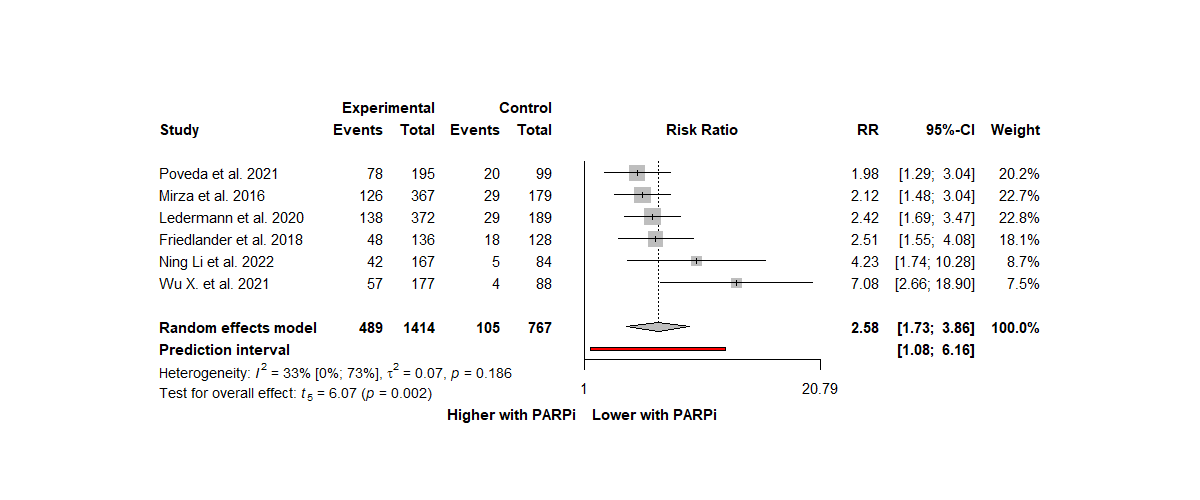
 **Figure S25** Forest plot representing that the PARPi maintenance therapy for recurrent ovarian cancer increase the risk of grade 3≤ vomiting versus placebo


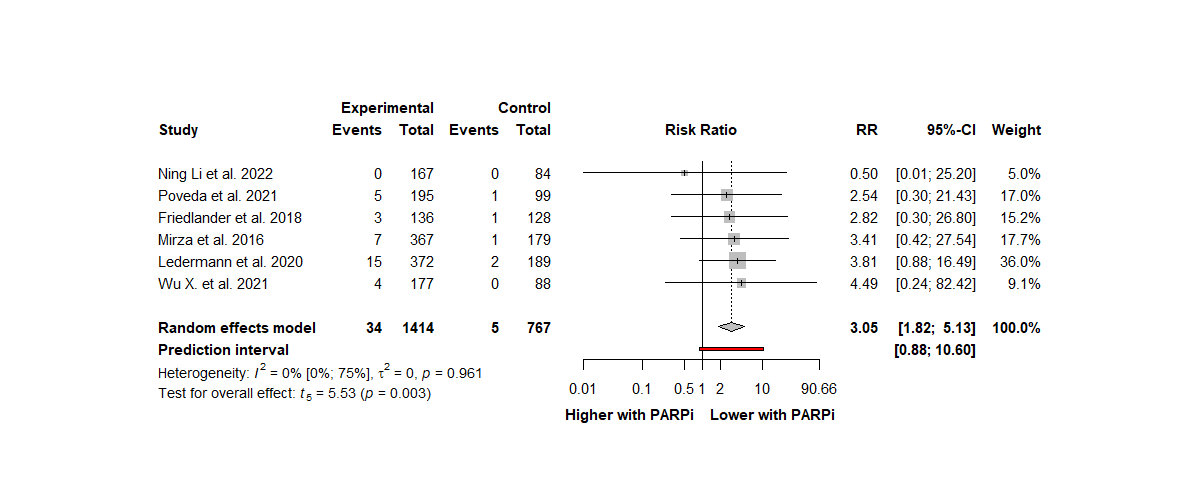


**Figure S26** Forest plot representing that the PARPi maintenance therapy for recurrent ovarian cancer increase the risk of dose modification versus placebo


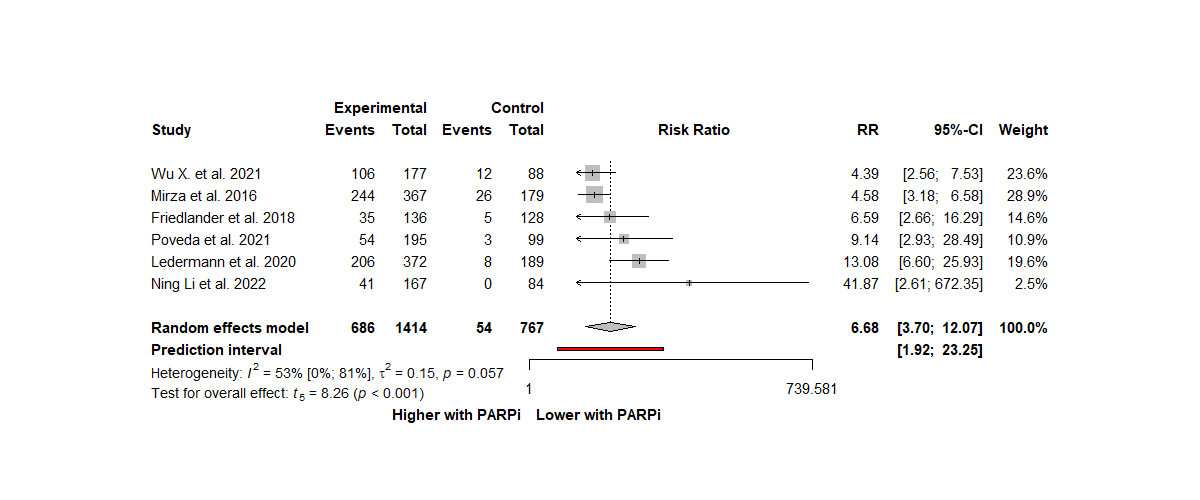


**Figure S27** Forest plot representing that the PARPi maintenance therapy for recurrent ovarian cancer increase the risk of treatment interruption versus placebo


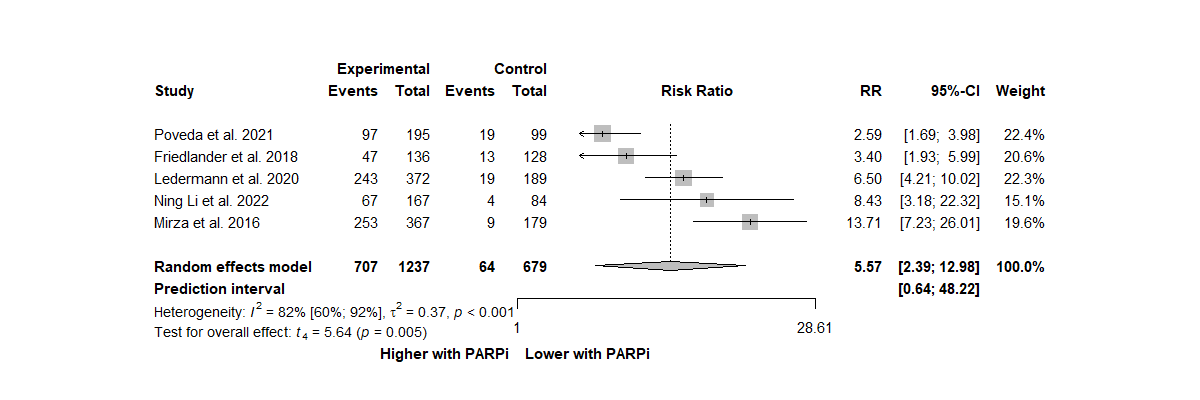


**Figure S28** Forest plot representing that the PARPi maintenance therapy for recurrent ovarian cancer increase the risk of treatment discontinuation versus placebo


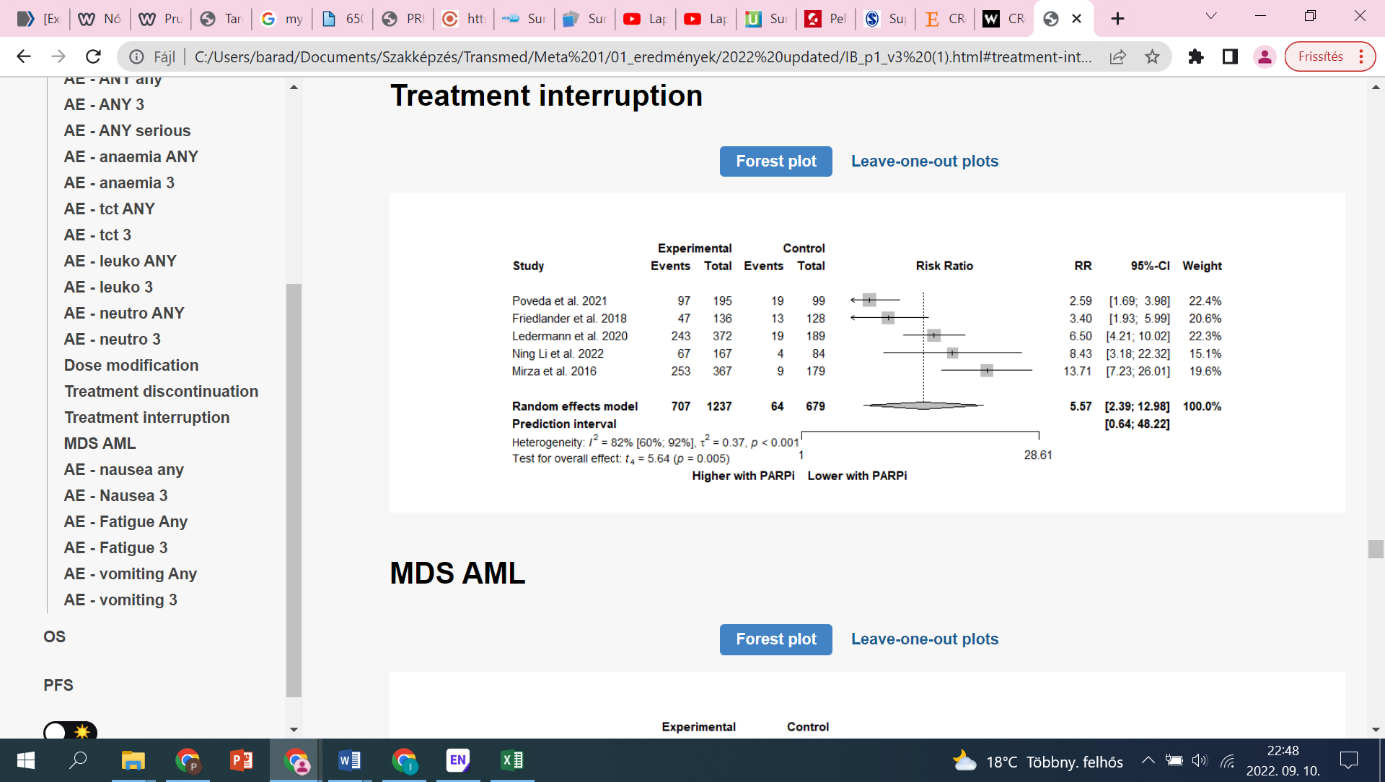


**Figure S29** Forest plot representing that the PARPi maintenance therapy for recurrent ovarian cancer increase the risk of MDS / AML versus placebo


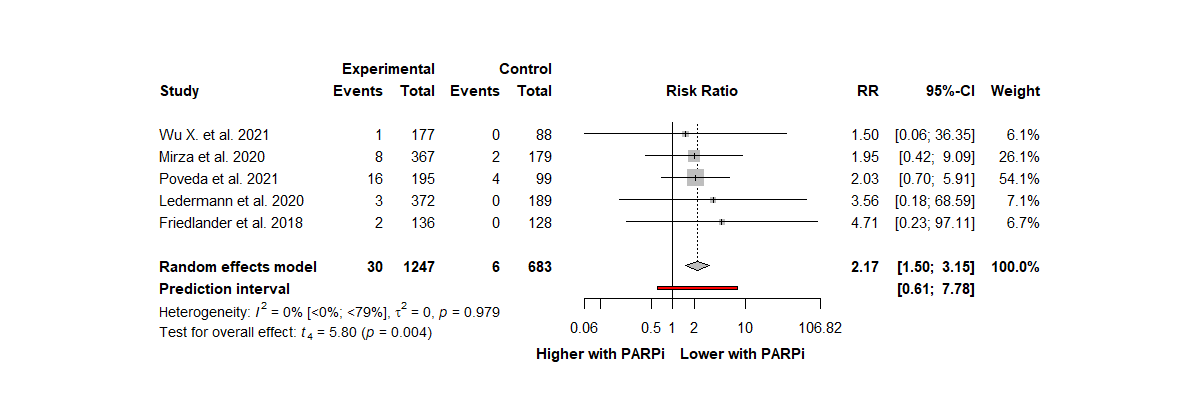
 **MDS / AML**: myelodysplastic syndrome or acute myeloid leukemia

**Figure S30** Forest plot representing that the PARPi monotherapy for recurrent ovarian cancer has little to no effect on the risk of anaemia by any grade versus chemotherapy


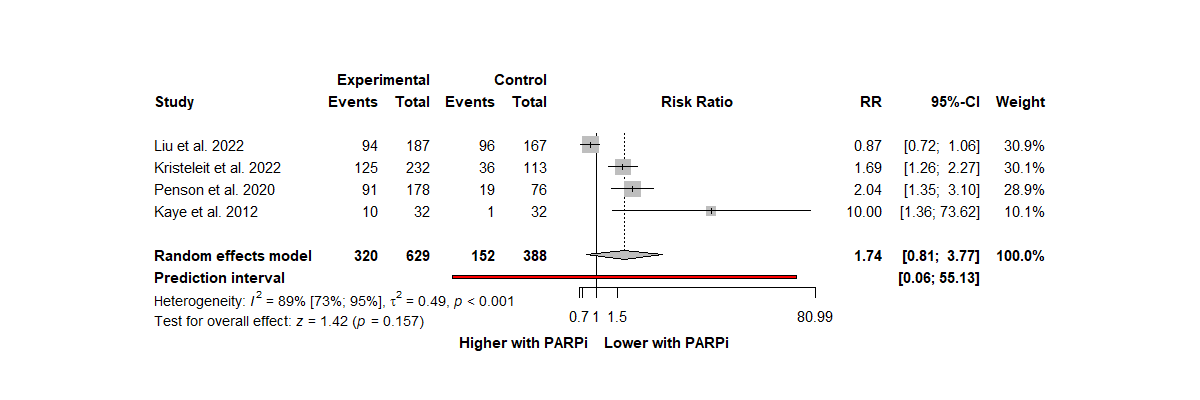


**Figure S31** Forest plot representing that the PARPi monotherapy for recurrent ovarian cancer increase the risk of grade 3≤ anaemia versus placebo


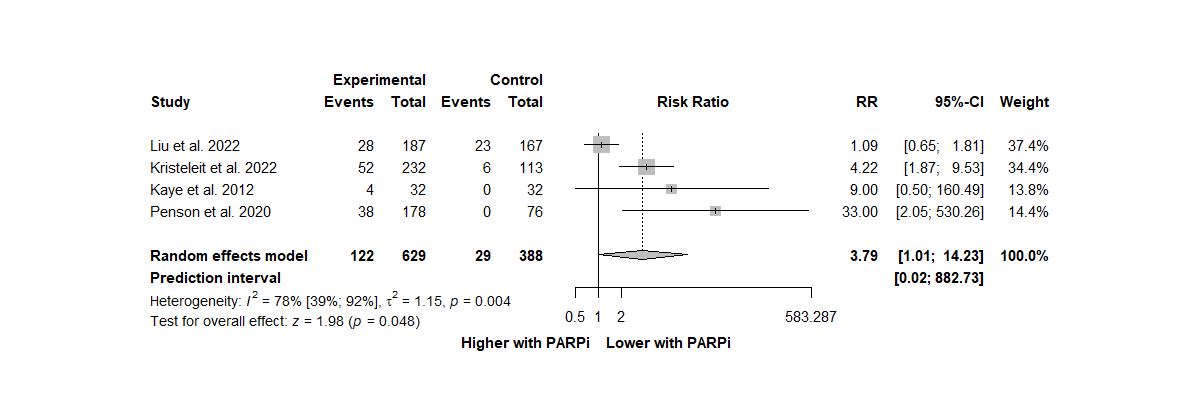


**Figure S32** Forest plot representing that the PARPi monotherapy for recurrent ovarian cancer has little to no effect on the risk of thrombocytopenia by any grade versus chemotherapy


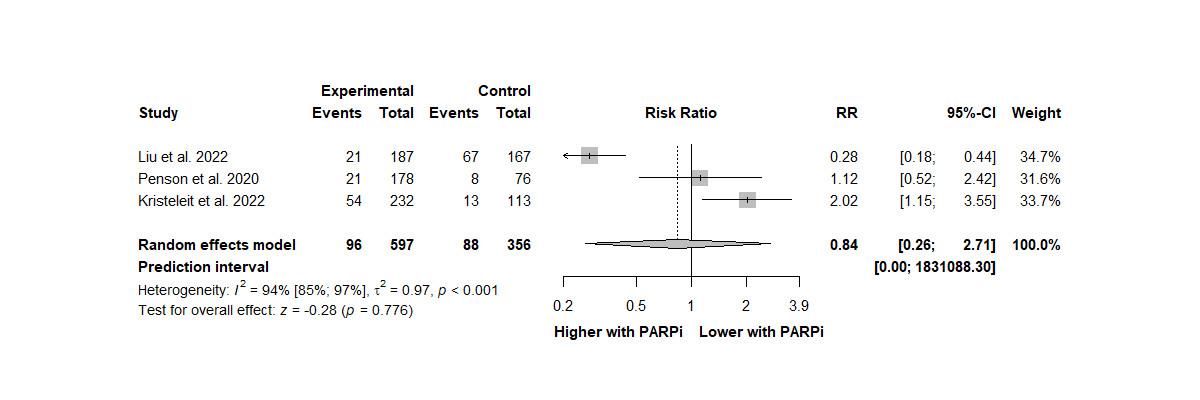


**Figure S33** Forest plot representing that the PARPi monotherapy for recurrent ovarian cancer has little to no effect on the risk of grade 3≤ thrombocytopenia versus chemotherapy


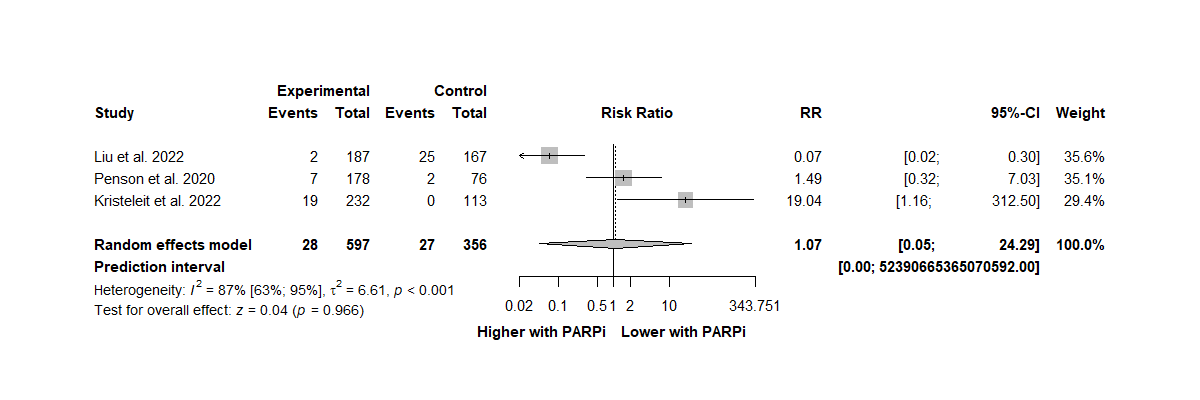


**Figure S34** Forest plot representing that the PARPi monotherapy for recurrent ovarian cancer decrease the risk of neutropenia by any grade versus chemotherapy


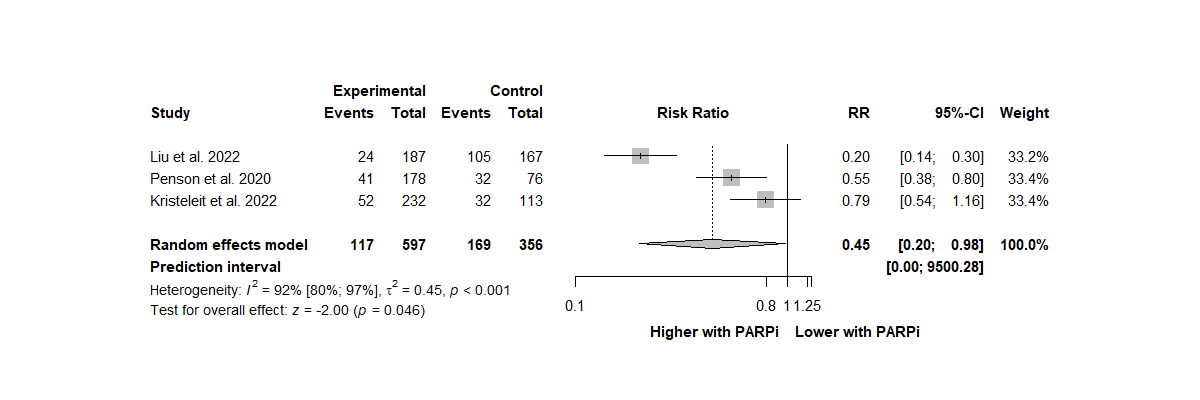


**Figure S35** Forest plot representing that the PARPi monotherapy for recurrent ovarian cancer has little to no effect on the risk of grade 3≤ neutropenia versus chemotherapy


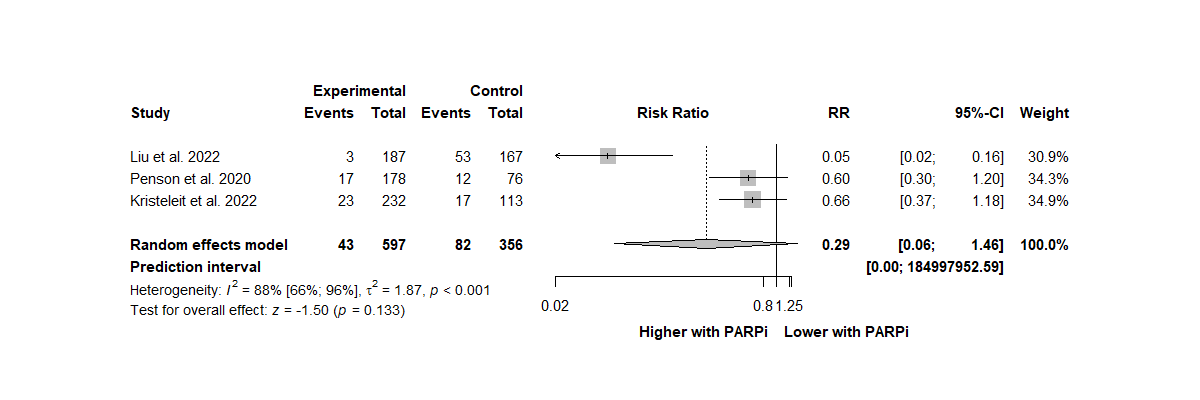


**Figure S36** Forest plot representing that the PARPi monotherapy for recurrent ovarian cancer increase the risk of nausea by any grade versus chemotherapy

**
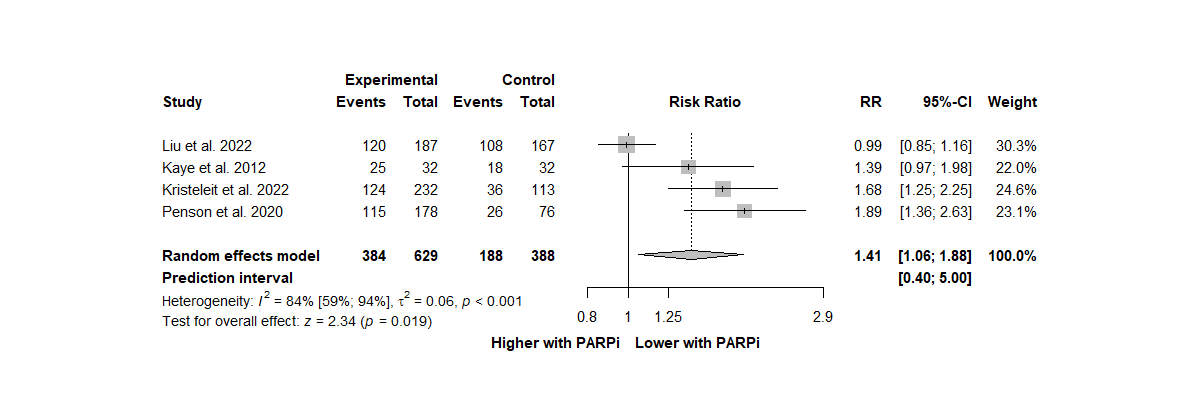
**

**Figure S37** Forest plot representing that the PARPi monotherapy for recurrent ovarian cancer has little to no effect on the risk of grade 3≤ nausea versus chemotherapy


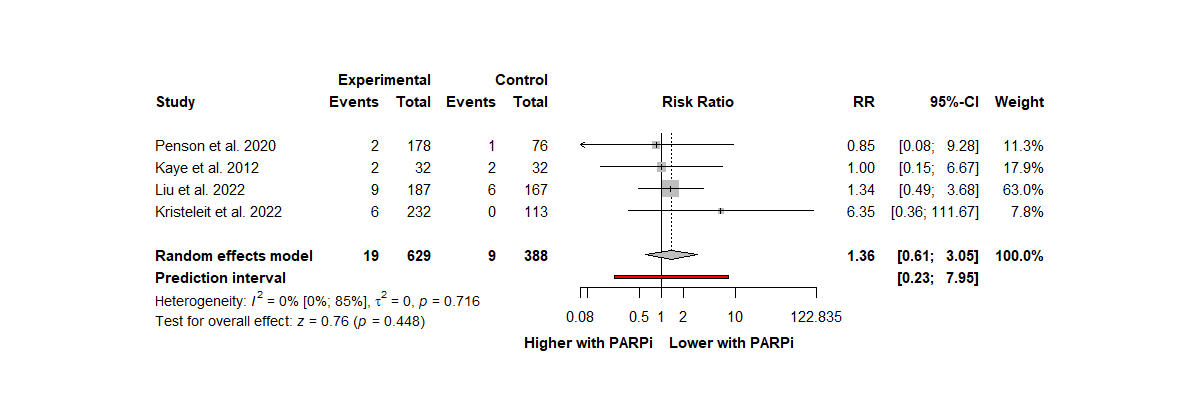


**Figure S38** Forest plot representing that the PARPi monotherapy for recurrent ovarian cancer increase the risk of fatigue by any grade versus chemotherapy


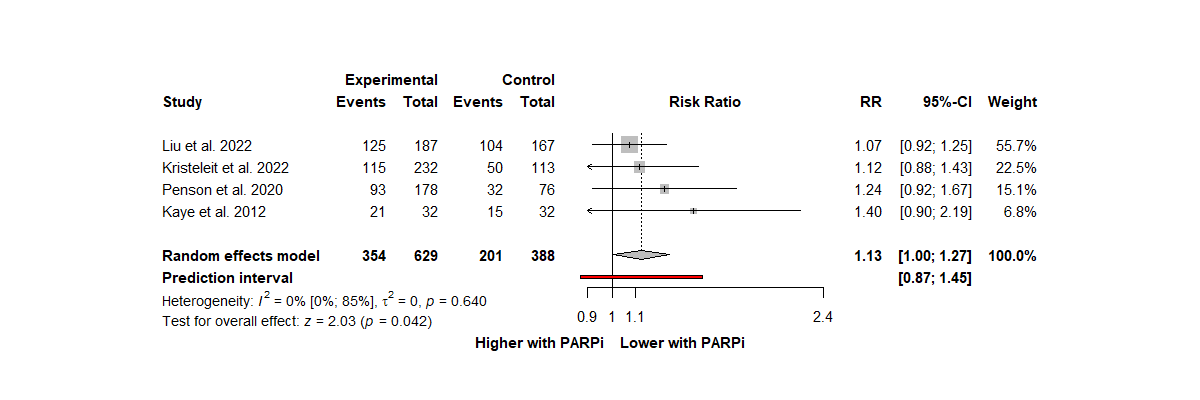


**Figure S39** Forest plot representing that the PARPi monotherapy for recurrent ovarian cancer increase the risk of grade 3≤ fatigue versus chemotherapy


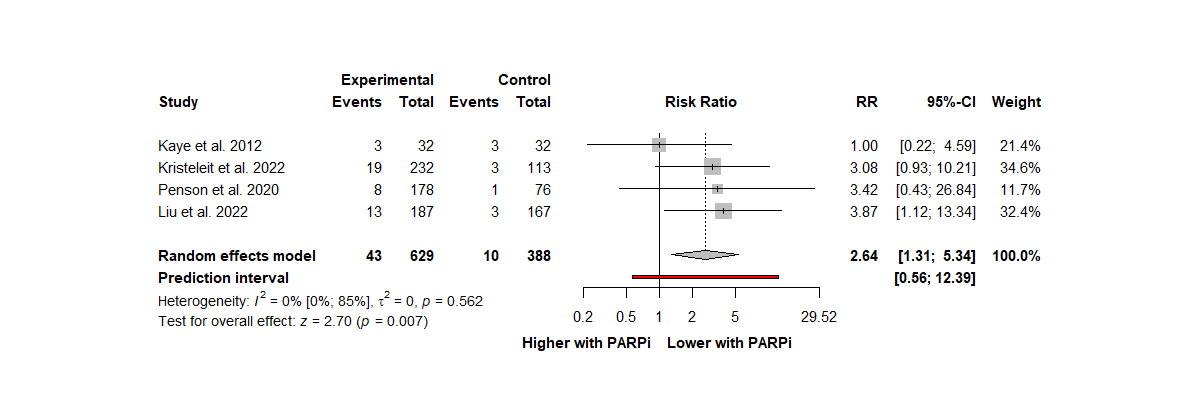


**Figure S40** Forest plot representing that the PARPi monotherapy for recurrent ovarian cancer increase the risk of vomiting by any grade versus chemotherapy

**
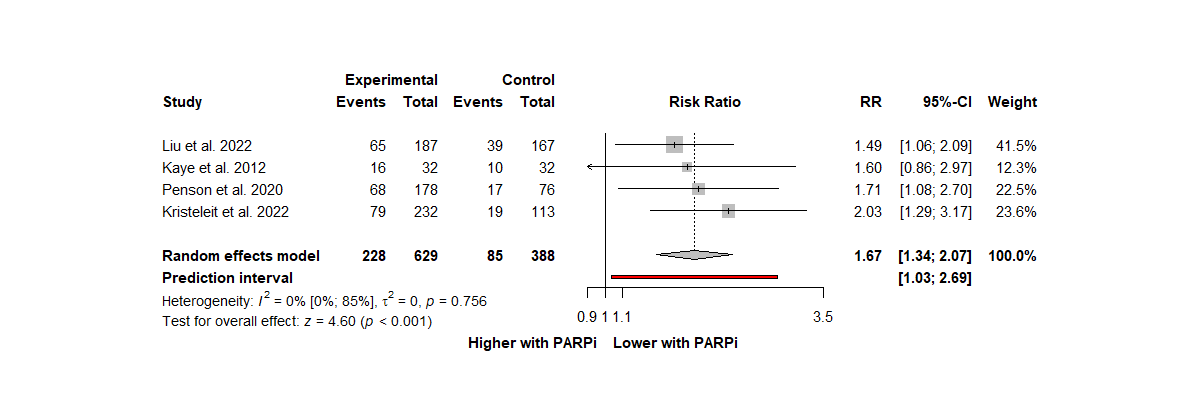
**

**Figure S41** Forest plot representing that the PARPi monotherapy for recurrent ovarian cancer has little to no effect on the risk of grade 3≤ vomiting versus chemotherapy


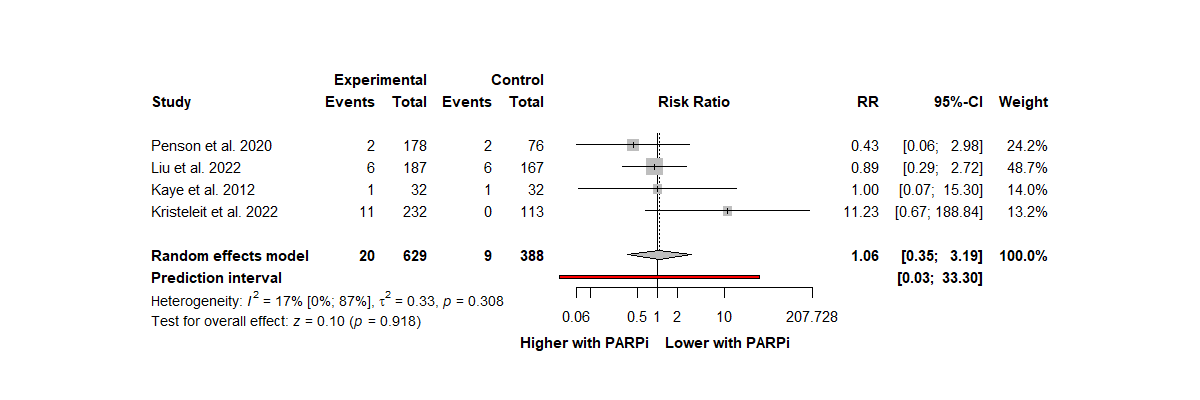


**Figure S42** Forest plot representing that the PARPi monotherapy for recurrent ovarian cancer has little to no effect on the risk of dose modification versus chemotherapy

**
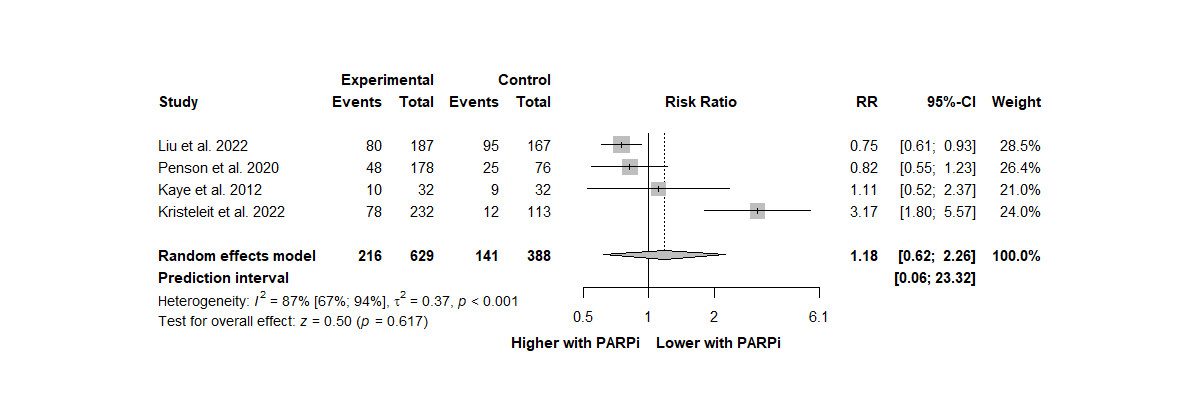
**

**Figure S43** Forest plot representing that the PARPi monotherapy for recurrent ovarian cancer has little to no effect on the risk of MDS / AML versus chemotherapy


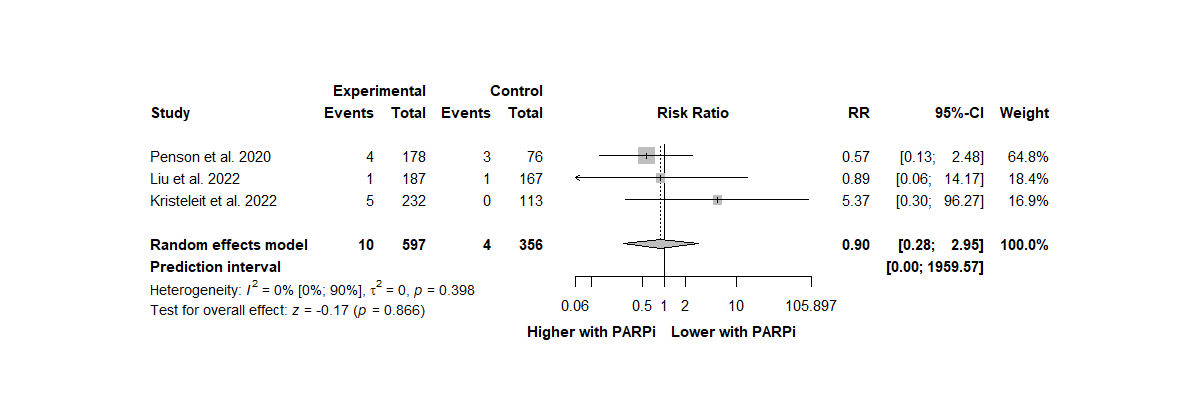
 **MDS / AML**: myelodysplastic syndrome or acute myeloid leukemia

**Figure S44** Forest plot representing that the PARPi maintenance therapy for newly-diagnosed ovarian cancer has little to no effect on the risk of adverse event by any grade versus placebo


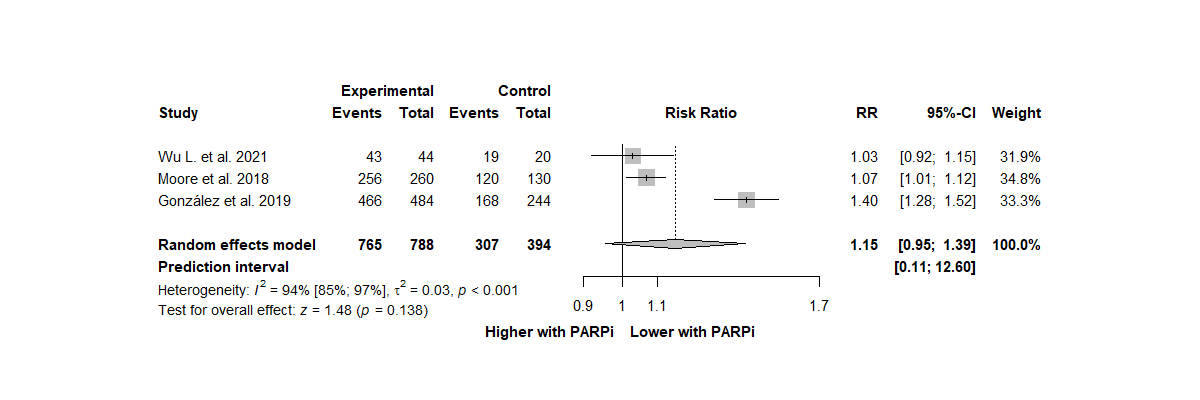


**Figure S45** Forest plot representing that the PARPi maintenance therapy for newly-diagnosed ovarian cancer increase the risk of grade 3≤ adverse events versus placebo


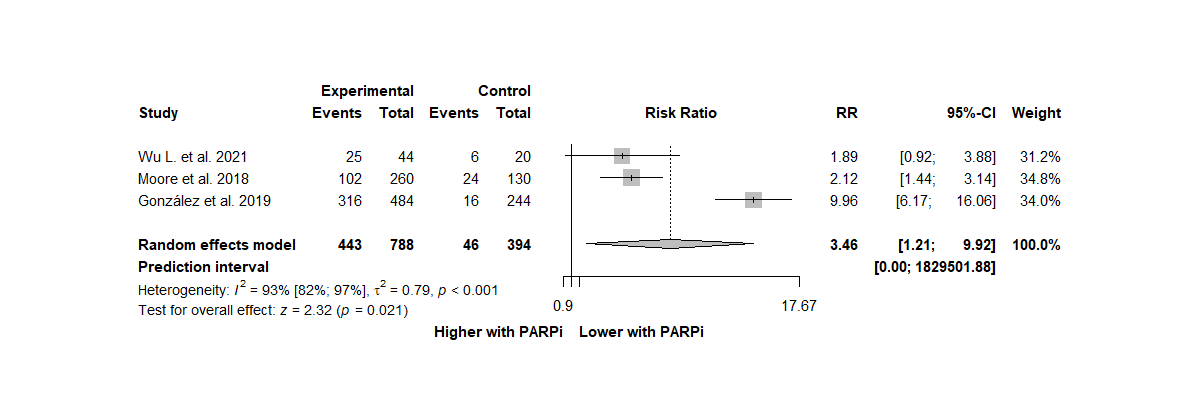


**Figure S46** Forest plot representing that the PARPi maintenance therapy for newly-diagnosed ovarian cancer increase the risk of serious adverse events versus placebo


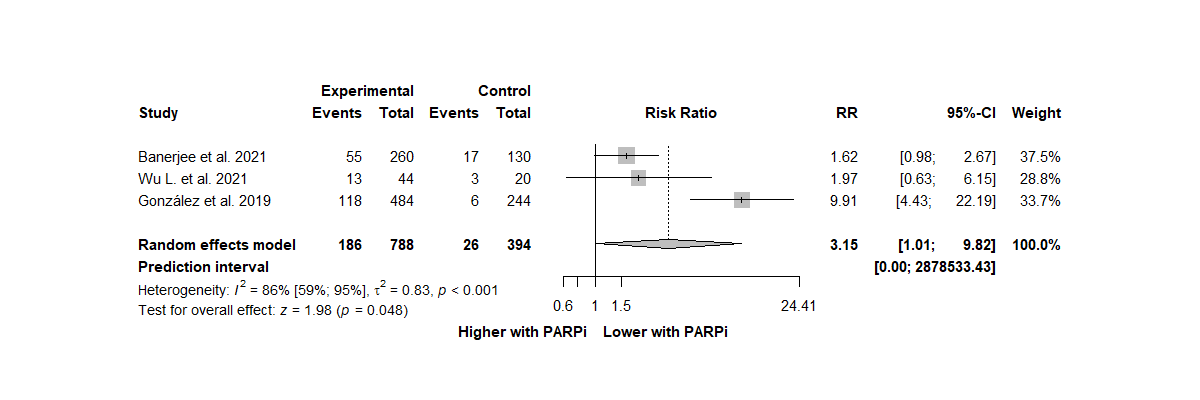
 **Figure S47** Forest plot representing that the PARPi maintenance therapy for newly-diagnosed ovarian cancer increase the risk of anaemia by any grade versus placebo


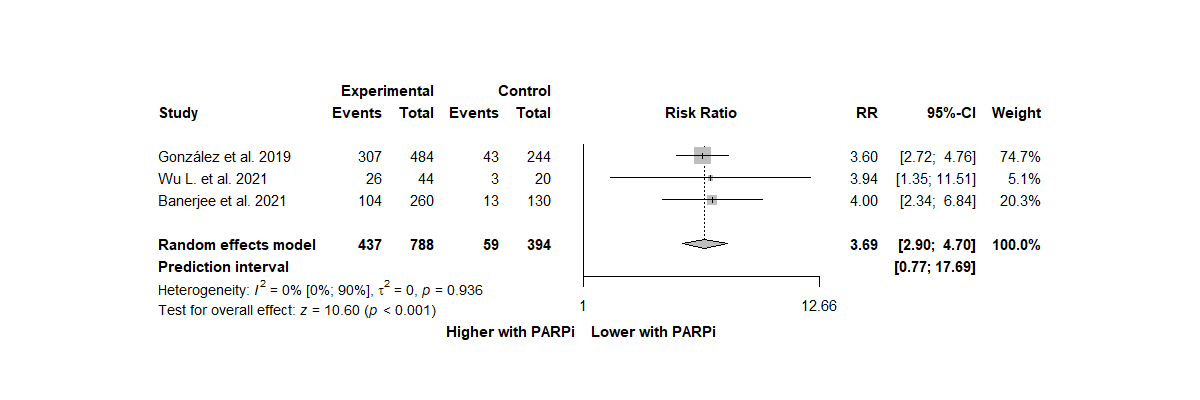


**Figure S48** Forest plot representing that the PARPi maintenance therapy for newly-diagnosed ovarian cancer increase the risk of grade 3≤ anaemia versus placebo


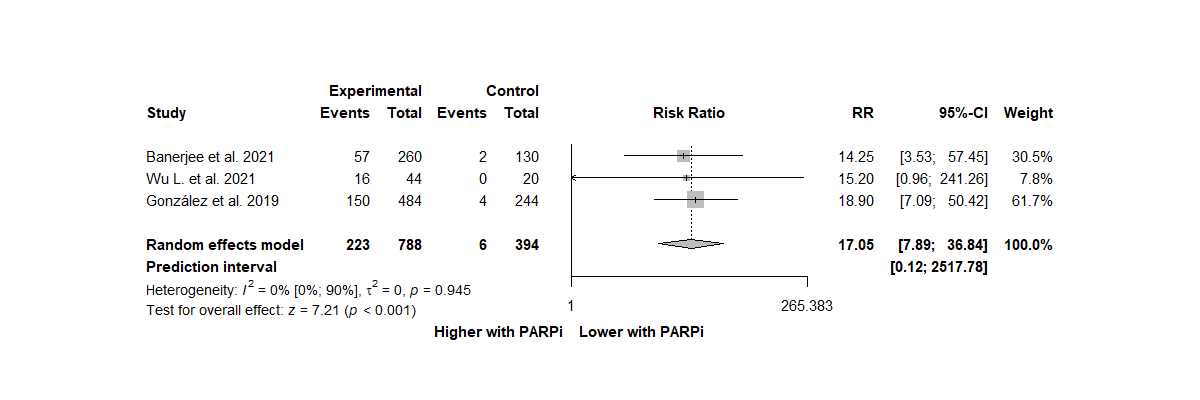


**Figure S49** Forest plot representing that the PARPi maintenance therapy for newly-diagnosed ovarian cancer increase the risk of thrombocytopenia by any grade versus placebo


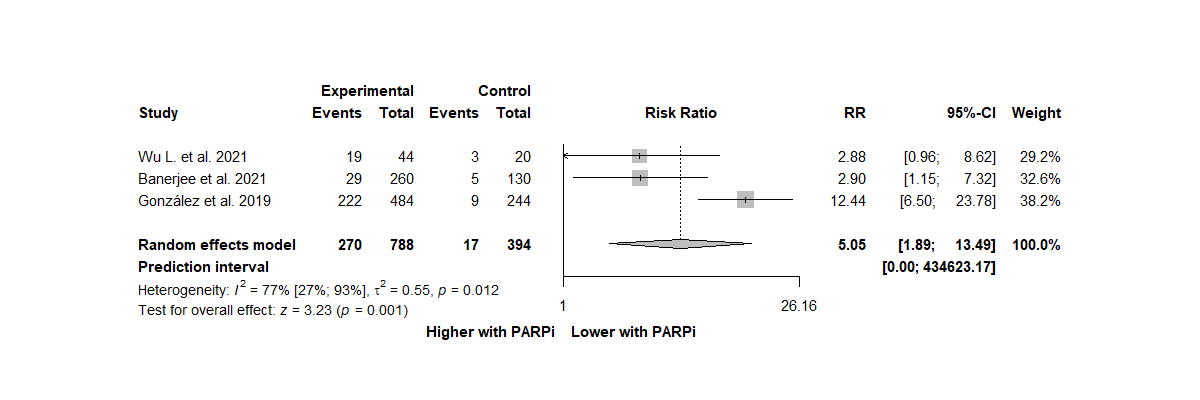


**Figure S50** Forest plot representing that the PARPi maintenance therapy for newly-diagnosed ovarian cancer has little to no effect on the risk of grade 3≤ thrombocytopenia versus placebo


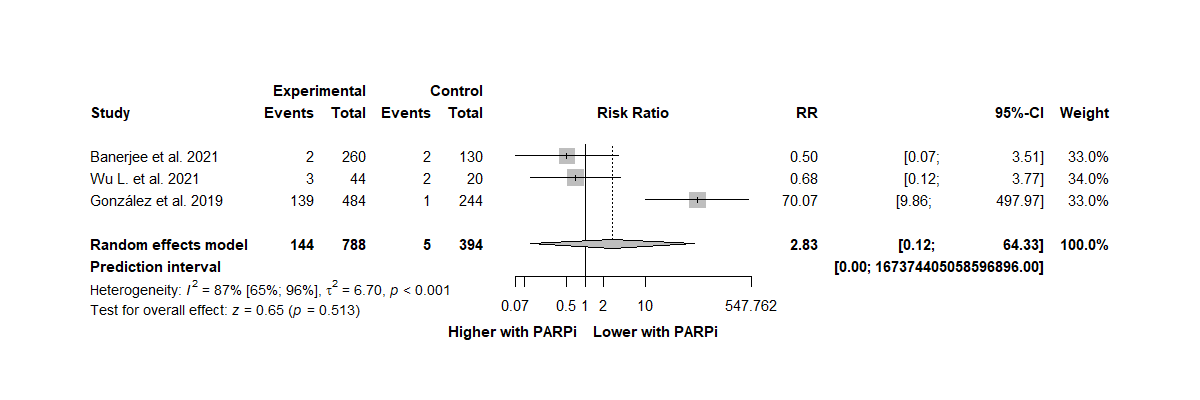


**Figure S51** Forest plot representing that the PARPi maintenance therapy for newly-diagnosed ovarian cancer increase the risk of neutropenia by any grade versus placebo


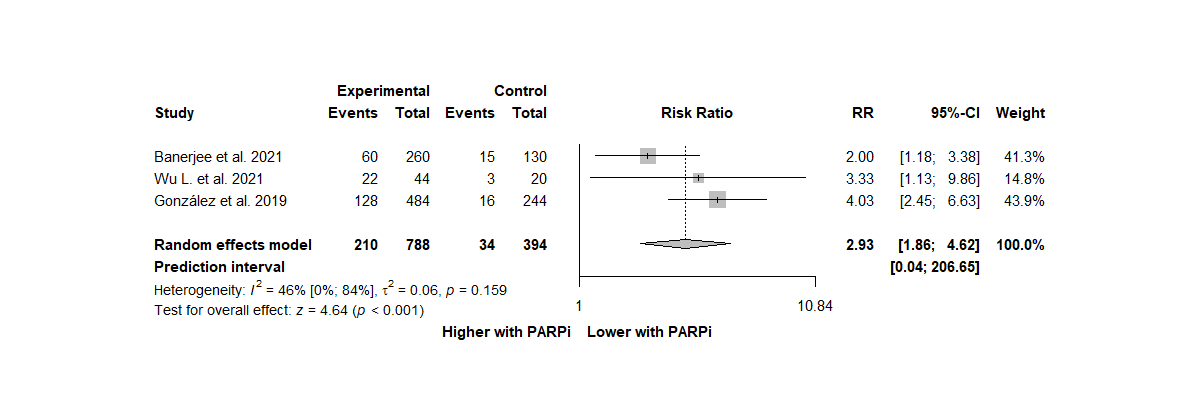


**Figure S52** Forest plot representing that the PARPi maintenance therapy for newly-diagnosed ovarian cancer increase the risk of grade 3≤ neutropenia versus placebo


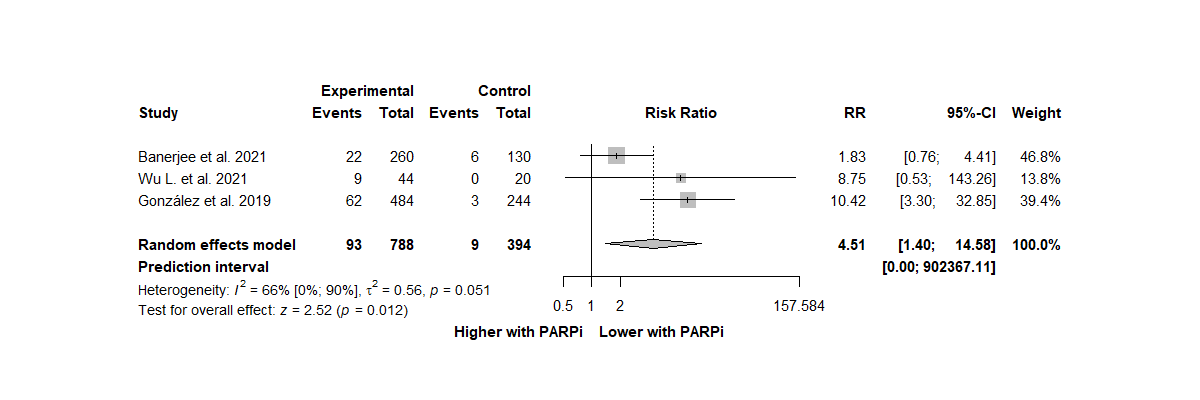


**Figure S53** Forest plot representing that the PARPi maintenance therapy for newly-diagnosed ovarian cancer increase the risk of nausea by any grade versus placebo


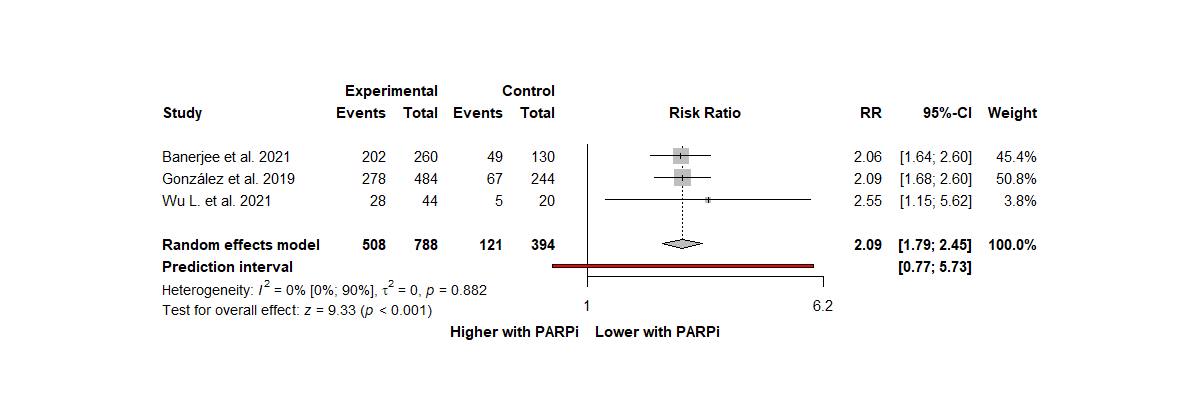


**Figure S54** Forest plot representing that the PARPi maintenance therapy for newly-diagnosed ovarian cancer has little to no effect on the risk of grade 3≤ nausea versus placebo


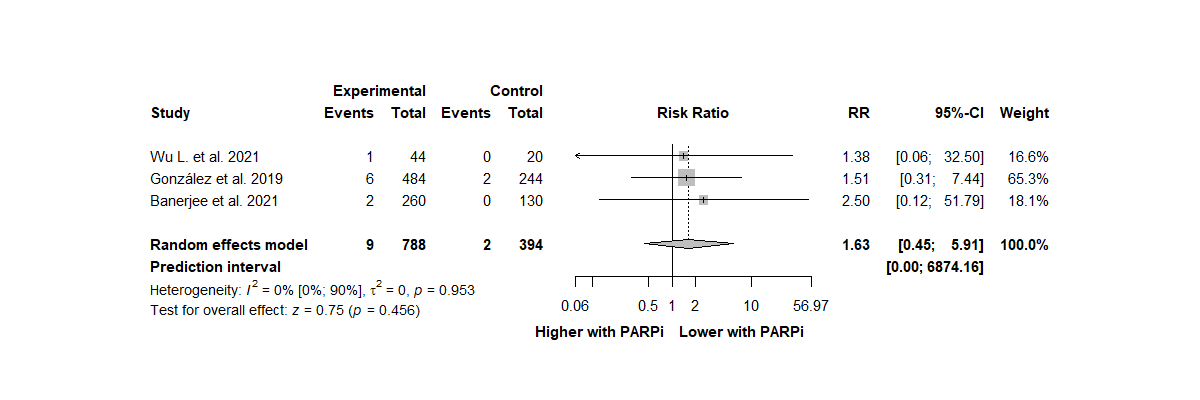


**Figure S55** Forest plot representing that the PARPi maintenance therapy for newly-diagnosed ovarian cancer increase the risk of fatigue by any grade versus placebo


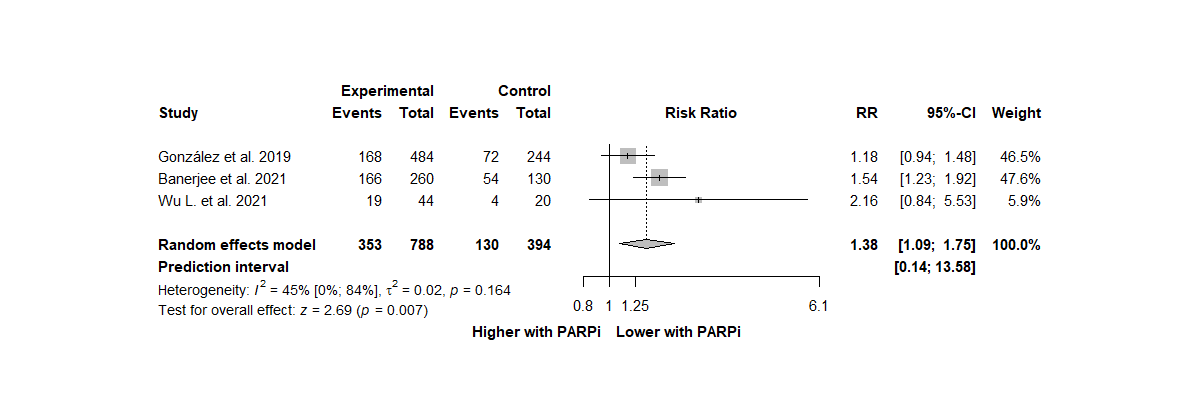


**Figure S56** Forest plot representing that the PARPi maintenance therapy for newly-diagnosed ovarian cancer has little to no effect on the risk of grade 3≤ fatigue versus placebo


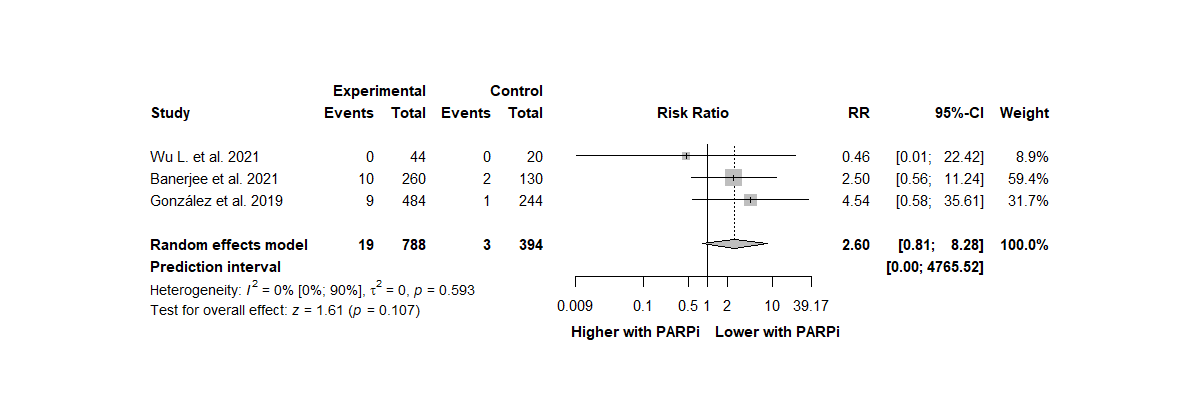


**Figure S57** Forest plot representing that the PARPi maintenance therapy for newly-diagnosed ovarian cancer increase the risk of vomiting by any grade versus placebo


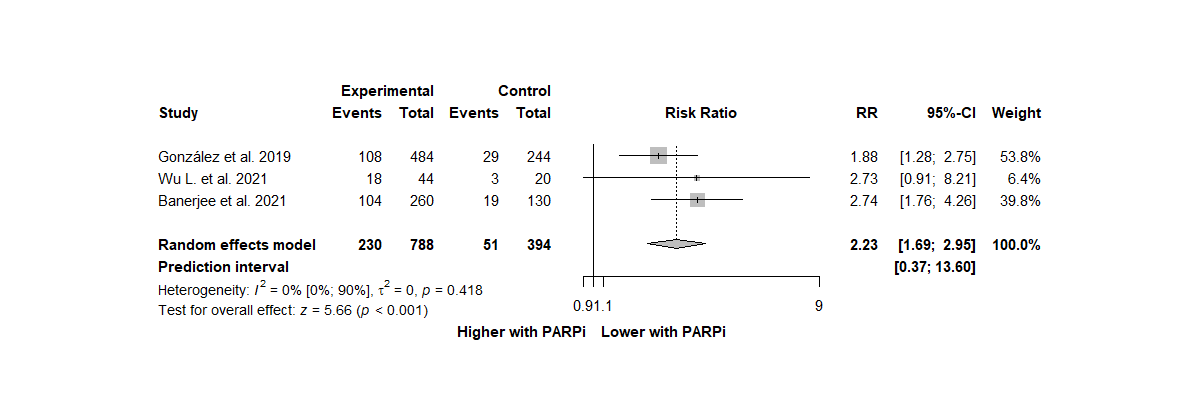


**Figure S58** Forest plot representing that the PARPi maintenance therapy for newly-diagnosed ovarian cancer has little to no effect on the risk of grade 3≤ vomiting versus placebo


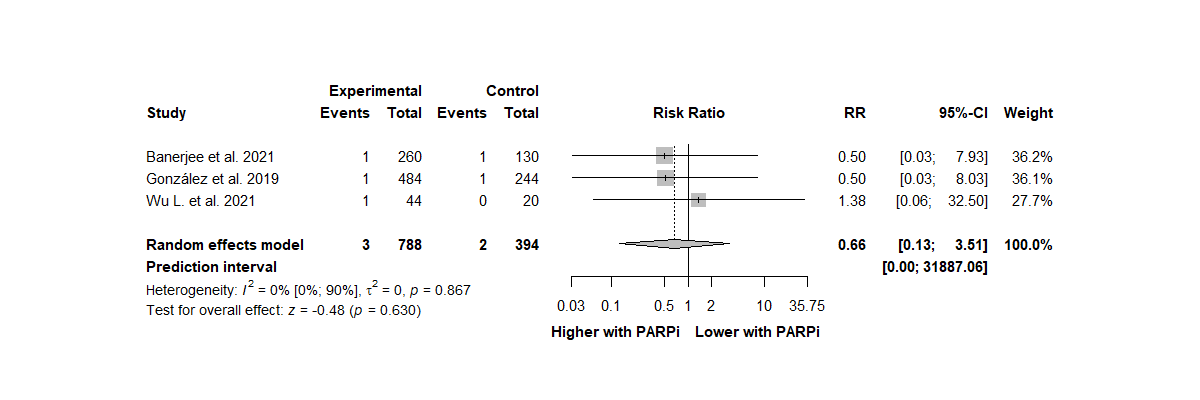


**Figure S59** Forest plot representing that the PARPi maintenance therapy for newly-diagnosed ovarian cancer increase the risk of dose modificaion versus placebo


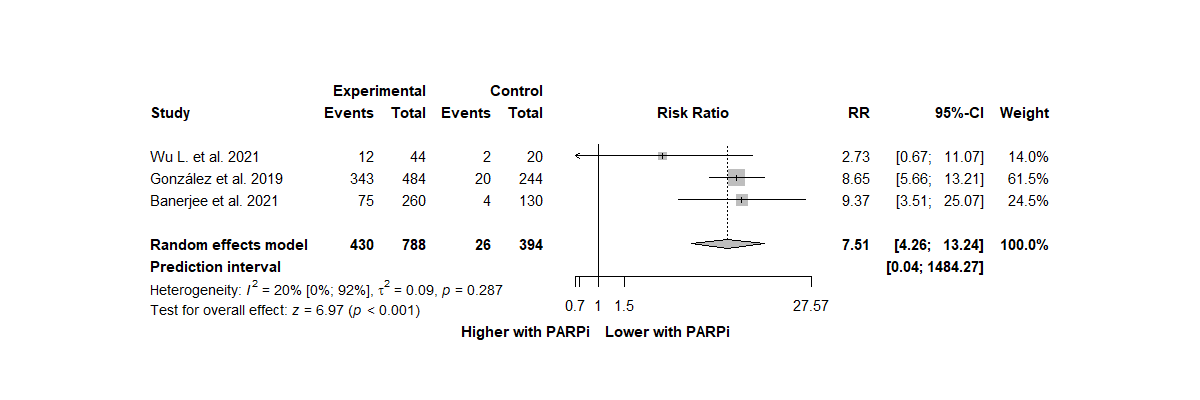


**Figure S60** Forest plot representing that the PARPi maintenance therapy for newly-diagnosed ovarian cancer increase the risk of treatmenet interruption versus placebo


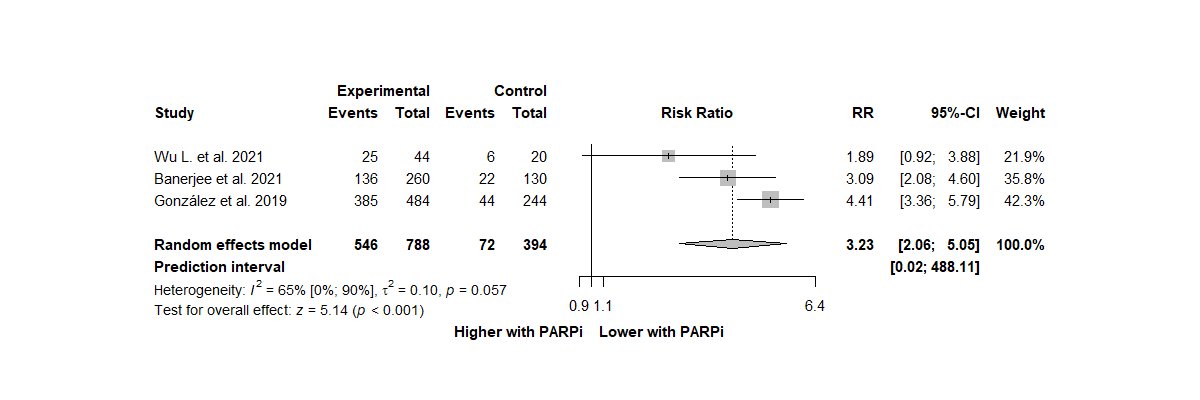


**Figure S61** Forest plot representing that the PARPi maintenance therapy for newly-diagnosed ovarian cancer increase the risk of treatmenet discontinuation versus placebo


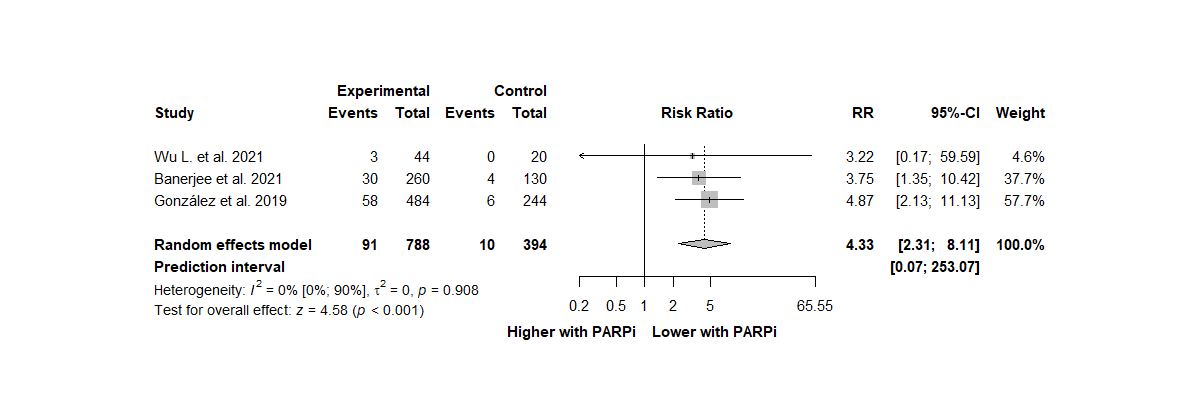


**Figure S62** Forest plot representing that the PARPi maintenance therapy for newly-diagnosed ovarian cancer has little to no effect on the risk of MDS / AML versus placebo


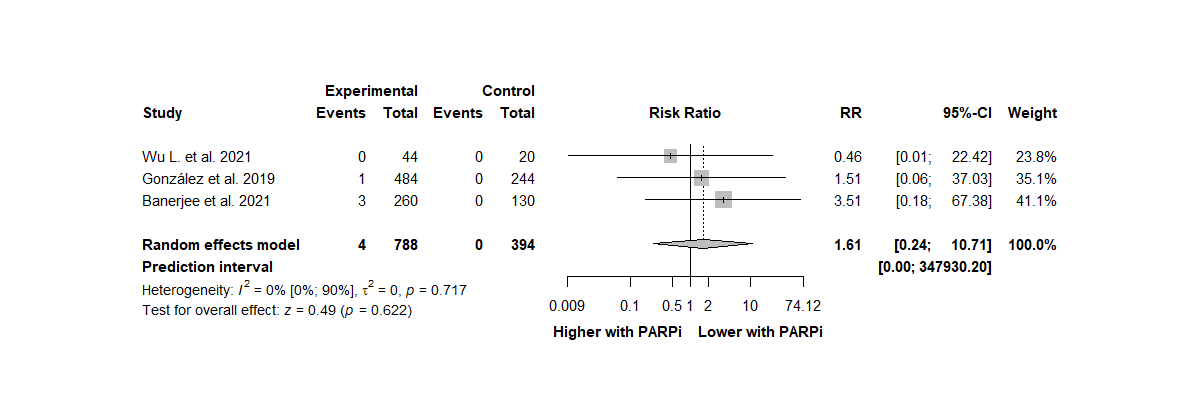
 **MDS / AML**: myelodysplastic syndrome or acute myeloid leukemia

**Figure S63** **Risk of bias summary at study level: for each included trial**

| **Table S2** Summary of findings & quality of evidence – PFS in recurrent OC: PARPi maintenance vs. placebo | | | | | | | | | | | |
| --- | --- | --- | --- | --- | --- | --- | --- | --- | --- | --- | --- |
| **Certainty assessment** | | | | | | | **Summary of findings** | | | | |
| **Participants (studies) Follow-up** | **Risk of bias** | **Inconsistency** | **Indirectness** | **Imprecision** | **Publication bias** | **Overall certainty of evidence** | **Study event rates (%)** | | **Relative effect (95% CI)** | **Anticipated absolute effects** | |
|  |  |  |  |  |  |  | **With placebo** | **With PARPi** |  | **Risk with placebo** | **Risk difference with PARPi** |
| **Total population** | | | | | | | | | | | |
| 2194 (7 RCTs) | not serious | not serious | serious | not serious | none | ⨁⨁⨁◯ Moderate | 771 participants | 1423 participants | **HR 0.34** (0.29 to 0.40) [PFS all pts] | **Low** | |
|  |  |  |  |  |  |  |  |  |  | 0 per 1 000 | **-- per 1 000** (from -- to --) |
| **BRCAw** | | | | | | | | | | | |
| 394 (3 RCTs) | not serious | not serious | not serious | not serious | none | ⨁⨁⨁⨁ High | 159 participants | 235 participants | **HR 0.50** (0.39 to 0.65) [PFS BRCAw pts] | **Low** | |
|  |  |  |  |  |  |  |  |  |  | 0 per 1 000 | **-- per 1 000** (from -- to --) |
| **BRCAm** | | | | | | | | | | | |
| 1030 (6 RCTs) | not serious | not serious | not serious | not serious | none | ⨁⨁⨁⨁ High | 361 participants | 669 participants | **HR 0.24** (0.18 to 0.31) [PFS BRCAm pts] | **Low** | |
|  |  |  |  |  |  |  |  |  |  | 0 per 1 000 | **-- per 1 000** (from -- to --) |
| **gBRACm** | | | | | | | | | | | |
| 533 (4 RCTs) | not serious | serious | not serious | not serious | none | ⨁⨁⨁◯ Moderate | 182 participants | 351 participants | **HR 0.23** (0.18 to 0.30) [PFS gBRACm pts] | **Low** | |
|  |  |  |  |  |  |  |  |  |  | 0 per 1 000 | **-- per 1 000** (from -- to --) |

**CI:** confidence interval; **HR:** hazard Ratio; **BRCAw**: BRCA wild-type; **BRCAm**: BRCA mutated; **gBRCAm**: germline BRCA mutated

| **Table S3** Summary of findings & quality of evidence – PFS in recurrent OC: PARPi monotherapy vs. chemotherapy | | | | | | | | | | | |
| --- | --- | --- | --- | --- | --- | --- | --- | --- | --- | --- | --- |
| **Certainty assessment** | | | | | | | **Summary of findings** | | | | |
| **Participants (studies) Follow-up** | **Risk of bias** | **Inconsistency** | **Indirectness** | **Imprecision** | **Publication bias** | **Overall certainty of evidence** | **Study event rates (%)** | | **Relative effect (95% CI)** | **Anticipated absolute effects** | |
|  |  |  |  |  |  |  | **With chemo** | **With PARPi** |  | **Risk with chemo** | **Risk difference with PARPi** |
| **Total population** | | | | | | | | | | | |
| 1055 (4 RCTs) | not serious | serious | serious | serious | none | ⨁◯◯◯ Very low | 424 participants | 631 participants | **HR 0.76** (0.51 to 1.14) [PFS all pts] | **Low** | |
|  |  |  |  |  |  |  |  |  |  | 0 per 1 000 | **-- per 1 000** (from -- to --) |
| **BRCAm** | | | | | | | | | | | |
| 769 (4 RCTs) | not serious | serious | not serious | serious | none | ⨁⨁◯◯ Low | 281 participants | 488 participants | **HR 0.62** (0.51 to 0.76) [PFS BRCAm pts] | **Low** | |
|  |  |  |  |  |  |  |  |  |  | 0 per 1 000 | **-- per 1 000** (from -- to --) |

**CI:** confidence interval; **HR:** hazard Ratio; **BRCAm**: BRCA mutated

**Table S4** Summary of findings & quality of evidence – PFS in newly-diagnosed OC: PARPi maintenance vs. placebo

|  | | | | | | | | | | | |
| --- | --- | --- | --- | --- | --- | --- | --- | --- | --- | --- | --- |
| **Certainty assessment** | | | | | | | **Summary of findings** | | | | |
| **Participants (studies) Follow-up** | **Risk of bias** | **Inconsistency** | **Indirectness** | **Imprecision** | **Publication bias** | **Overall certainty of evidence** | **Study event rates (%)** | | **Relative effect (95% CI)** | **Anticipated absolute effects** | |
|  |  |  |  |  |  |  | **With placebo** | **With PARPi** |  | **Risk with placebo** | **Risk difference with PARPi** |
| **Total population** | | | | | | | | | | | |
| 1188 (3 RCTs) | not serious | serious | serious | not serious | none | ⨁⨁◯◯ Low | 397 participants | 791 participants | **HR 0.46** (0.30 to 0.71) [PFS all pts] | **Low** | |
|  |  |  |  |  |  |  |  |  |  | 0 per 1 000 | **-- per 1 000** (from -- to --) |
| **BRCAm** | | | | | | | | | | | |
| 678 (3 RCTs) | not serious | not serious | not serious | not serious | none | ⨁⨁⨁⨁ High | 222 participants | 456 participants | **HR 0.36** (0.29 to 0.44) [PFS BRCAm pts] | **Low** | |
|  |  |  |  |  |  |  |  |  |  | 0 per 1 000 | **-- per 1 000** (from -- to --) |

**CI:** confidence interval; **HR:** hazard Ratio; **BRCAm**: BRCA mutated

**Table S5** Summary of findings & quality of evidence – AEs in recurrent OC: PARPi maintenance vs. placebo

| **Certainty assessment** | | | | | | | **Summary of findings** | | | | |
| --- | --- | --- | --- | --- | --- | --- | --- | --- | --- | --- | --- |
| **Participants (studies) Follow-up** | **Risk of bias** | **Inconsistency** | **Indirectness** | **Imprecision** | **Publication bias** | **Overall certainty of evidence** | **Study event rates (%)** | | **Relative effect (95% CI)** | **Anticipated absolute effects** | |
|  |  |  |  |  |  |  | **With placebo** | **With PARPi** |  | **Risk with placebo** | **Risk difference with PARPi** |
| **Any adverse event** | | | | | | | | | | | |
| 2181 (6 RCTs) | not serious | not serious | serious | not serious | none | ⨁⨁⨁◯ Moderate | 729/767 (95.0%) | 1404/1414 (99.3%) | **RR 1.04** (1.04 to 1.05) | 950 per 1 000 | **38 more per 1 000** (from 38 more to 48 more) |
| **Any grade 3 & grade 4 adverse event** | | | | | | | | | | | |
| 1635 (5 RCTs) | not serious | serious | serious | not serious | none | ⨁⨁◯◯ Low | 94/588 (16.0%) | 519/1047 (49.6%) | **RR 2.98** (1.82 to 4.87) | 160 per 1 000 | **317 more per 1 000** (from 131 more to 619 more) |
| **Serious adverse events** | | | | | | | | | | | |
| 1917 (5 RCTs) | not serious | serious | serious | not serious | none | ⨁⨁◯◯ Low | 37/639 (5.8%) | 236/1278 (18.5%) | **RR 3.27** (1.45 to 7.36) | 58 per 1 000 | **131 more per 1 000** (from 26 more to 368 more) |
| **Anaemia** | | | | | | | | | | | |
| 2181 (6 RCTs) | not serious | serious | serious | serious | none | ⨁◯◯◯ Very low | 78/767 (10.2%) | 641/1414 (45.3%) | **RR 4.18** (2.36 to 7.40) | 102 per 1 000 | **323 more per 1 000** (from 138 more to 651 more) |
| **Anaemia grade 3 & grade 4** | | | | | | | | | | | |
| 2181 (6 RCTs) | not serious | very serious | serious | very serious | none | ⨁◯◯◯ Very low | 6/767 (0.8%) | 292/1414 (20.7%) | **RR 14.26** (5.33 to 38.12) | 8 per 1 000 | **104 more per 1 000** (from 34 more to 290 more) |
| **Thrombocytopenia** | | | | | | | | | | | |
| 2181 (6 RCTs) | not serious | very serious | serious | serious | none | ⨁◯◯◯ Very low | 57/767 (7.4%) | 552/1414 (39.0%) | **RR 4.36** (1.90 to 10.02) | 74 per 1 000 | **250 more per 1 000** (from 67 more to 670 more) |
| **Thrombocytopenia grade 3 & grade 4** | | | | | | | | | | | |
| 2181 (6 RCTs) | not serious | very serious | serious | very serious | none | ⨁◯◯◯ Very low | 8/767 (1.0%) | 197/1414 (13.9%) | **RR 6.86** (1.45 to 32.35) | 10 per 1 000 | **61 more per 1 000** (from 5 more to 327 more) |
| **Leukopenia** | | | | | | | | | | | |
| 810 (3 RCTs) | not serious | very serious | serious | serious | none | ⨁◯◯◯ Very low | 61/271 (22.5%) | 221/539 (41.0%) | **RR 2.27** (0.88 to 5.87) | 225 per 1 000 | **286 more per 1 000** (from 27 fewer to 1 000 more) |
| **Leukopenia grade 3 & grade 4** | | | | | | | | | | | |
| 810 (3 RCTs) | not serious | very serious | serious | very serious | none | ⨁◯◯◯ Very low | 2/271 (0.7%) | 35/539 (6.5%) | **RR 4.69** (1.43 to 15.37) | 7 per 1 000 | **27 more per 1 000** (from 3 more to 106 more) |
| **Neutropenia** | | | | | | | | | | | |
| 2181 (6 RCTs) | not serious | serious | serious | not serious | none | ⨁⨁◯◯ Low | 84/767 (11.0%) | 416/1414 (29.4%) | **RR 2.78** (1.58 to 4.87) | 110 per 1 000 | **195 more per 1 000** (from 64 more to 424 more) |
| **Neutropenia grade 3 & grade 4** | | | | | | | | | | | |
| 2181 (6 RCTs) | not serious | very serious | serious | very serious | none | ⨁◯◯◯ Very low | 16/767 (2.1%) | 172/1414 (12.2%) | **RR 4.33** (1.58 to 11.86) | 21 per 1 000 | **69 more per 1 000** (from 12 more to 227 more) |
| **Dose modification** | | | | | | | | | | | |
| 2181 (6 RCTs) | not serious | very serious | serious | serious | none | ⨁◯◯◯ Very low | 56/767 (7.3%) | 686/1414 (48.5%) | **RR 6.68** (3.70 to 12.07) | 73 per 1 000 | **415 more per 1 000** (from 197 more to 808 more) |
| **Treatment discontinuation** | | | | | | | | | | | |
| 2181 (6 RCTs) | not serious | very serious | serious | serious | none | ⨁◯◯◯ Very low | 33/767 (4.3%) | 225/1414 (15.9%) | **RR 3.24** (1.20 to 8.77) | 43 per 1 000 | **96 more per 1 000** (from 9 more to 334 more) |
| **Treatment interruption** | | | | | | | | | | | |
| 1916 (5 RCTs) | not serious | very serious | serious | very serious | none | ⨁◯◯◯ Very low | 64/679 (9.4%) | 707/1237 (57.2%) | **RR 5.57** (2.39 to 12.98) | 94 per 1 000 | **431 more per 1 000** (from 131 more to 1 000 more) |
| **MDS / AML** | | | | | | | | | | | |
| 1930 (5 RCTs) | not serious | not serious | serious | serious | none | ⨁⨁◯◯ Low | 6/683 (0.9%) | 30/1247 (2.4%) | **RR 2.17** (1.50 to 3.15) | 9 per 1 000 | **10 more per 1 000** (from 4 more to 19 more) |
| **Nausea** | | | | | | | | | | | |
| 2181 (6 RCTs) | not serious | serious | serious | serious | none | ⨁◯◯◯ Very low | 244/767 (31.8%) | 986/1414 (69.7%) | **RR 2.17** (1.86 to 2.53) | 318 per 1 000 | **372 more per 1 000** (from 274 more to 487 more) |
| **Nausea grade 3 & grade 4** | | | | | | | | | | | |
| 2181 (6 RCTs) | not serious | serious | serious | very serious | none | ⨁◯◯◯ Very low | 3/767 (0.4%) | 62/1414 (4.4%) | **RR 1.36** (0.61 to 3.05) | 4 per 1 000 | **1 more per 1 000** (from 2 fewer to 8 more) |
| **Fatigue** | | | | | | | | | | | |
| 2181 (6 RCTs) | not serious | serious | serious | serious | none | ⨁◯◯◯ Very low | 293/767 (38.2%) | 803/1414 (56.8%) | **RR 1.50** (1.26 to 1.78) | 382 per 1 000 | **191 more per 1 000** (from 99 more to 298 more) |
| **Fatigue grade 3 & grade 4** | | | | | | | | | | | |
| 2181 (6 RCTs) | not serious | serious | serious | very serious | none | ⨁◯◯◯ Very low | 13/767 (1.7%) | 82/1414 (5.8%) | **RR 2.92** (1.53 to 5.55) | 17 per 1 000 | **33 more per 1 000** (from 9 more to 77 more) |
| **Vomiting** | | | | | | | | | | | |
| 2181 (6 RCTs) | not serious | serious | serious | not serious | none | ⨁⨁◯◯ Low | 105/767 (13.7%) | 489/1414 (34.6%) | **RR 2.58** (1.73 to 3.86) | 137 per 1 000 | **216 more per 1 000** (from 100 more to 392 more) |
| **Vomiting grade 3 & grade 4** | | | | | | | | | | | |
| 2181 (6 RCTs) | not serious | not serious | serious | serious | none | ⨁⨁◯◯ Low | 5/767 (0.7%) | 34/1414 (2.4%) | **RR 3.05** (1.82 to 5.13) | 7 per 1 000 | **13 more per 1 000** (from 5 more to 27 more) |

**CI:** confidence interval; **RR:** risk ratio; **MDS / AML**: myelodysplastic syndrome or acute myeloid leukemia

**Table S6** Summary of findings & quality of evidence – AEs in recurrent OC: PARPi monotherapy vs. chemotherapy

|  | | | | | | | | | | | |
| --- | --- | --- | --- | --- | --- | --- | --- | --- | --- | --- | --- |
| **Certainty assessment** | | | | | | | **Summary of findings** | | | | |
| **Participants (studies) Follow-up** | **Risk of bias** | **Inconsistency** | **Indirectness** | **Imprecision** | **Publication bias** | **Overall certainty of evidence** | **Study event rates (%)** | | **Relative effect (95% CI)** | **Anticipated absolute effects** | |
|  |  |  |  |  |  |  | **With chemo** | **With PARPi** |  | **Risk with chemo** | **Risk difference with PARPi** |
| **Anaemia** | | | | | | | | | | | |
| 1017 (4 RCTs) | not serious | serious | serious | serious | none | ⨁◯◯◯ Very low | 320/629 (50.9%) | 152/388 (39.2%) | **RR 1.74** (0.81 to 3.77) | 509 per 1 000 | **376 more per 1 000** (from 97 fewer to 1 000 more) |
| **Anaemia grade 3 & grade 4** | | | | | | | | | | | |
| 1017 (4 RCTs) | not serious | serious | serious | very serious | none | ⨁◯◯◯ Very low | 29/388 (7.5%) | 122/629 (19.4%) | **RR 3.79** (1.01 to 14.23) | 75 per 1 000 | **209 more per 1 000** (from 1 more to 989 more) |
| **Thrombocytopenia** | | | | | | | | | | | |
| 953 (4 RCTs) | not serious | serious | serious | very serious | none | ⨁◯◯◯ Very low | 88/356 (24.7%) | 96/597 (16.1%) | **RR 0.84** (0.26 to 2.71) | 247 per 1 000 | **40 fewer per 1 000** (from 183 fewer to 423 more) |
| **Thrombocytopenia grade 3 & grade 4** | | | | | | | | | | | |
| 953 (3 RCTs) | not serious | very serious | serious | extremely serious | none | ⨁◯◯◯ Very low | 27/356 (7.6%) | 28/597 (4.7%) | **RR 1.07** (0.05 to 24.29) | 76 per 1 000 | **5 more per 1 000** (from 72 fewer to 1 000 more) |
| **Neutropenia** | | | | | | | | | | | |
| 953 (3 RCTs) | not serious | serious | serious | serious | none | ⨁◯◯◯ Very low | 169/356 (47.5%) | 117/597 (19.6%) | **RR 0.45** (0.20 to 0.98) | 475 per 1 000 | **261 fewer per 1 000** (from 380 fewer to 9 fewer) |
| **Neutropenia grade 3 & grade 4** | | | | | | | | | | | |
| 953 (3 RCTs) | not serious | serious | serious | serious | none | ⨁◯◯◯ Very low | 82/356 (23.0%) | 43/597 (7.2%) | **RR 0.29** (0.06 to 1.46) | 230 per 1 000 | **164 fewer per 1 000** (from 217 fewer to 106 more) |
| **Dose modification** | | | | | | | | | | | |
| 1017 (4 RCTs) | not serious | serious | serious | serious | none | ⨁◯◯◯ Very low | 141/388 (36.3%) | 216/629 (34.3%) | **RR 1.18** (0.62 to 2.26) | 363 per 1 000 | **65 more per 1 000** (from 138 fewer to 458 more) |
| **MDS / AML** | | | | | | | | | | | |
| 953 (4 RCTs) | not serious | serious | serious | serious | none | ⨁◯◯◯ Very low | 4/356 (1.1%) | 10/597 (1.7%) | **RR 0.90** (0.28 to 2.95) | 11 per 1 000 | **1 fewer per 1 000** (from 8 fewer to 22 more) |
| **Nausea** | | | | | | | | | | | |
| 1017 (4 RCTs) | not serious | not serious | serious | serious | none | ⨁⨁◯◯ Low | 188/388 (48.5%) | 384/629 (61.0%) | **RR 1.41** (1.06 to 1.88) | 485 per 1 000 | **199 more per 1 000** (from 29 more to 426 more) |
| **Nausea grade 3 & grade 4** | | | | | | | | | | | |
| 1017 (4 RCTs) | not serious | not serious | serious | serious | none | ⨁⨁◯◯ Low | 9/388 (2.3%) | 19/629 (3.0%) | **RR 1.36** (0.61 to 3.05) | 23 per 1 000 | **8 more per 1 000** (from 9 fewer to 48 more) |
| **Fatigue** | | | | | | | | | | | |
| 1017 (4 RCTs) | not serious | serious | serious | very serious | none | ⨁◯◯◯ Very low | 201/388 (51.8%) | 354/629 (56.3%) | **RR 1.13** (1.00 to 1.27) | 518 per 1 000 | **67 more per 1 000** (from 0 fewer to 140 more) |
| **Vomiting grade 3 & grade 4** | | | | | | | | | | | |
| 1017 (4 RCTs) | not serious | not serious | serious | not serious | none | ⨁⨁⨁◯ Moderate | 85/388 (21.9%) | 228/629 (36.2%) | **RR 1.67** (1.34 to 2.07) | 219 per 1 000 | **147 more per 1 000** (from 74 more to 234 more) |
| **Vomiting grade 3 & grade 4** | | | | | | | | | | | |
| 1017 (4 RCTs) | not serious | serious | serious | serious | none | ⨁◯◯◯ Very low | 9/388 (2.3%) | 20/629 (3.2%) | **RR 1.06** (0.35 to 3.19) | 23 per 1 000 | **1 more per 1 000** (from 15 fewer to 51 more) |

**CI:** confidence interval; **RR:** risk ratio; **MDS / AML**: myelodysplastic syndrome or acute myeloid leukemia

**Table S7** Summary of findings & quality of evidence – AEs in newly-diagnosed OC: PARPi maintenance vs. placebo

|  | | | | | | | | | | | |
| --- | --- | --- | --- | --- | --- | --- | --- | --- | --- | --- | --- |
| **Certainty assessment** | | | | | | | **Summary of findings** | | | | |
| **Participants (studies) Follow-up** | **Risk of bias** | **Inconsistency** | **Indirectness** | **Imprecision** | **Publication bias** | **Overall certainty of evidence** | **Study event rates (%)** | | **Relative effect (95% CI)** | **Anticipated absolute effects** | |
|  |  |  |  |  |  |  | **With placebo** | **With PARPi** |  | **Risk with placebo** | **Risk difference with PARPi** |
| **Any adverse event** | | | | | | | | | | | |
| 1182 (3 RCTs) | not serious | serious | serious | serious | none | ⨁◯◯◯ Very low | 307/394 (77.9%) | 765/788 (97.1%) | **RR 1.15** (0.95 to 1.39) | 779 per 1 000 | **117 more per 1 000** (from 39 fewer to 304 more) |
| **Any grade 3 & grade 4 adverse event** | | | | | | | | | | | |
| 1182 (3 RCTs) | not serious | serious | serious | very serious | none | ⨁◯◯◯ Very low | 46/394 (11.7%) | 443/788 (56.2%) | **RR 3.46** (1.12 to 9.92) | 117 per 1 000 | **287 more per 1 000** (from 14 more to 1 000 more) |
| **Serious adverse events** | | | | | | | | | | | |
| 1182 (3 RCTs) | not serious | serious | serious | very serious | none | ⨁◯◯◯ Very low | 26/394 (6.6%) | 186/788 (23.6%) | **RR 3.15** (1.01 to 9.82) | 66 per 1 000 | **142 more per 1 000** (from 1 more to 582 more) |
| **Anaemia** | | | | | | | | | | | |
| 1182 (3 RCTs) | not serious | not serious | serious | not serious | none | ⨁⨁⨁◯ Moderate | 59/394 (15.0%) | 437/788 (55.5%) | **RR 3.69** (2.90 to 4.70) | 150 per 1 000 | **403 more per 1 000** (from 285 more to 554 more) |
| **Anaemia grade 3 & grade 4** | | | | | | | | | | | |
| 1182 (3 RCTs) | not serious | not serious | serious | serious | none | ⨁⨁◯◯ Low | 6/394 (1.5%) | 223/788 (28.3%) | **RR 17.05** (7.89 to 36.84) | 15 per 1 000 | **244 more per 1 000** (from 105 more to 546 more) |
| **Thrombocytopenia** | | | | | | | | | | | |
| 1182 (3 RCTs) | not serious | serious | serious | serious | none | ⨁◯◯◯ Very low | 17/394 (4.3%) | 270/788 (34.3%) | **RR 5.05** (1.89 to 13.49) | 43 per 1 000 | **175 more per 1 000** (from 38 more to 539 more) |
| **Thrombocytopenia grade 3 & grade 4** | | | | | | | | | | | |
| 1182 (3 RCTs) | not serious | very serious | serious | very serious | none | ⨁◯◯◯ Very low | 5/394 (1.3%) | 144/788 (18.3%) | **RR 2.83** (0.12 to 64.33) | 13 per 1 000 | **23 more per 1 000** (from 11 fewer to 804 more) |
| **Neutropenia** | | | | | | | | | | | |
| 1182 (3 RCTs) | not serious | serious | serious | serious | none | ⨁◯◯◯ Very low | 34/394 (8.6%) | 210/788 (26.6%) | **RR 2.93** (1.86 to 4.62) | 86 per 1 000 | **167 more per 1 000** (from 74 more to 312 more) |
| **Neutropenia grade 3 & grade 4** | | | | | | | | | | | |
| 1182 (3 RCTs) | not serious | serious | serious | serious | none | ⨁◯◯◯ Very low | 9/394 (2.3%) | 93/788 (11.8%) | **RR 4.51** (1.40 to 14.58) | 23 per 1 000 | **80 more per 1 000** (from 9 more to 310 more) |
| **Dose modification** | | | | | | | | | | | |
| 1182 (3 RCTs) | not serious | serious | serious | not serious | none | ⨁⨁◯◯ Low | 26/394 (6.6%) | 430/788 (54.6%) | **RR 7.51** (4.26 to 13.24) | 66 per 1 000 | **430 more per 1 000** (from 215 more to 808 more) |
| **Treatment discontinuation** | | | | | | | | | | | |
| 1182 (3 RCTs) | not serious | not serious | serious | serious | none | ⨁⨁◯◯ Low | 10/394 (2.5%) | 91/788 (11.5%) | **RR 4.33** (2.31 to 8.11) | 25 per 1 000 | **85 more per 1 000** (from 33 more to 180 more) |
| **Treatment interruption** | | | | | | | | | | | |
| 1182 (3 RCTs) | not serious | serious | serious | not serious | none | ⨁⨁◯◯ Low | 72/394 (18.3%) | 546/788 (69.3%) | **RR 3.23** (2.06 to 5.05) | 183 per 1 000 | **408 more per 1 000** (from 194 more to 740 more) |
| **MDS / AML** | | | | | | | | | | | |
| 1182 (3 RCTs) | not serious | serious | serious | very serious | none | ⨁◯◯◯ Very low | 0/394 (0.0%) | 4/788 (0.5%) | **RR 1.61** (0.24 to 10.71) | 0 per 1 000 | **0 fewer per 1 000** (from 0 fewer to 0 fewer) |
| **Nausea** | | | | | | | | | | | |
| 1182 (3 RCTs) | not serious | not serious | serious | not serious | none | ⨁⨁⨁◯ Moderate | 121/394 (30.7%) | 508/788 (64.5%) | **RR 2.09** (1.79 to 2.45) | 307 per 1 000 | **335 more per 1 000** (from 243 more to 445 more) |
| **Nausea grade 3 & grade 4** | | | | | | | | | | | |
| 1182 (3 RCTs) | not serious | not serious | serious | very serious | none | ⨁◯◯◯ Very low | 2/394 (0.5%) | 9/788 (1.1%) | **RR 1.63** (0.45 to 5.91) | 5 per 1 000 | **3 more per 1 000** (from 3 fewer to 25 more) |
| **Fatigue** | | | | | | | | | | | |
| 1182 (3 RCTs) | not serious | not serious | serious | very serious | none | ⨁◯◯◯ Very low | 130/394 (33.0%) | 353/788 (44.8%) | **RR 1.63** (0.45 to 5.91) | 330 per 1 000 | **208 more per 1 000** (from 181 fewer to 1 000 more) |
| **Fatigue grade 3 & grade 4** | | | | | | | | | | | |
| 1182 (3 RCTs) | not serious | serious | serious | very serious | none | ⨁◯◯◯ Very low | 3/394 (0.8%) | 19/788 (2.4%) | **RR 2.60** (0.81 to 8.28) | 8 per 1 000 | **12 more per 1 000** (from 1 fewer to 55 more) |
| **Vomiting** | | | | | | | | | | | |
| 1182 (3 RCTs) | not serious | not serious | serious | not serious | none | ⨁⨁⨁◯ Moderate | 51/394 (12.9%) | 230/788 (29.2%) | **RR 2.23** (1.69 to 2.95) | 129 per 1 000 | **159 more per 1 000** (from 89 more to 252 more) |
| **Vomiting grade 3 & grade 4** | | | | | | | | | | | |
| 1182 (3 RCTs) | not serious | serious | serious | serious | none | ⨁◯◯◯ Very low | 2/394 (0.5%) | 3/788 (0.4%) | **RR 0.66** (0.13 to 3.51) | 5 per 1 000 | **2 fewer per 1 000** (from 4 fewer to 13 more) |

**CI:** confidence interval; **RR:** risk ratio; **MDS / AML**: myelodysplastic syndrome or acute myeloid leukemia
